# Correction: Radiomics analysis for the early diagnosis of common sexually transmitted infections and skin lesions

**DOI:** 10.1371/journal.pdig.0001079

**Published:** 2025-10-31

**Authors:** Jiajun Sun, Zhen Yu, Yingping Li, Janet M. Towns, Lin Zhang, Jason J. Ong, Zongyuan Ge, Christopher K. Fairley, Lei Zhang

The second author, Zhen You’s email address appear incorrectly. The correct email address is: Zhen.Yu1@monash.edu; lei.zhang1@monash.edu.

The Funding statement for this article is incorrect. The correct Funding statement is as follows: This work was supported by the National Key R&D Program of China (2022YFC2304900); Outstanding Young Scholars Support Program (3111500001 [LZ]); Xi’an Jiaotong University Basic Research and Profession Grant (xtr022019003 [LZ], xzy032020032 [LZ]); and Xi’an Jiaotong University Young Scholar Support Grant (YX6J004 [LZ]).
