# Supplementary figures and images for: Radiomics analysis for the early diagnosis of common sexually transmitted infections and skin lesions
Source: PLOS Digit Health. 2025 Jul 23;4(7):e0000926. doi: 10.1371/journal.pdig.0000926 (PMC12286352; doi:10.1371/journal.pdig.0000926)

S1 Fig. The structure of the typical STI prediction models.


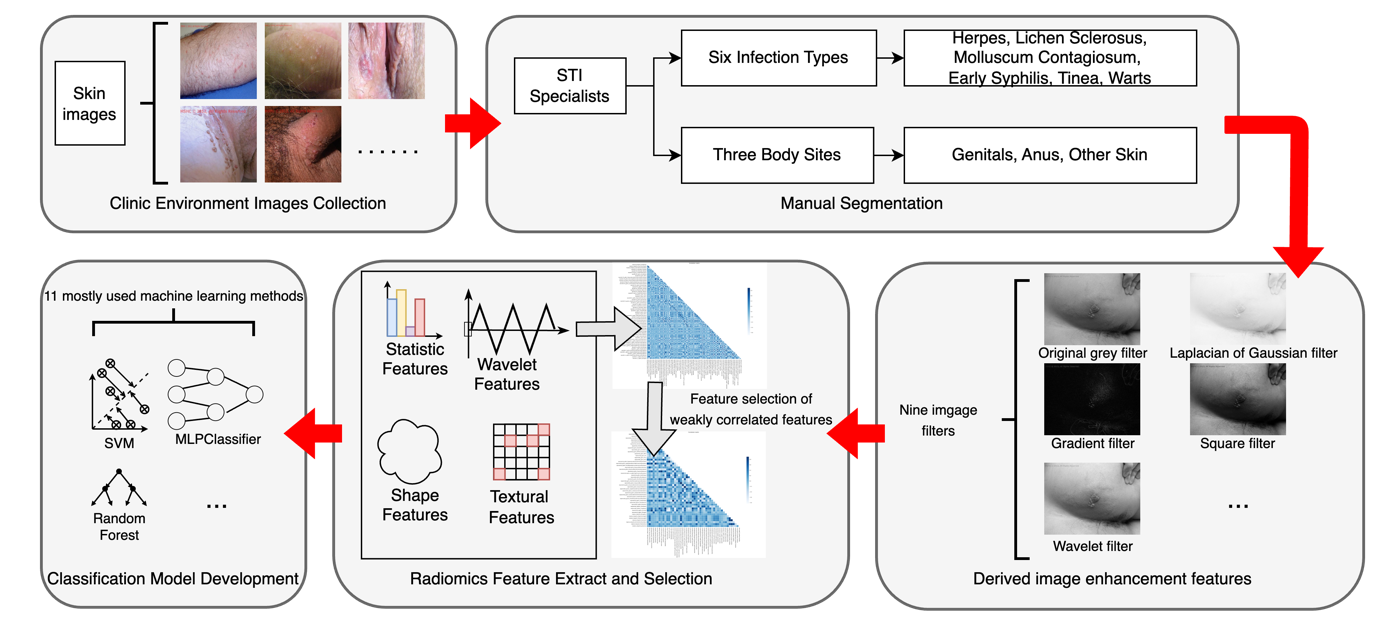

Supplement: S1 Fig — (DOCX) [file pdig.0000926.s001.docx]

S3 Fig. Methods of dividing images into training and testing data.


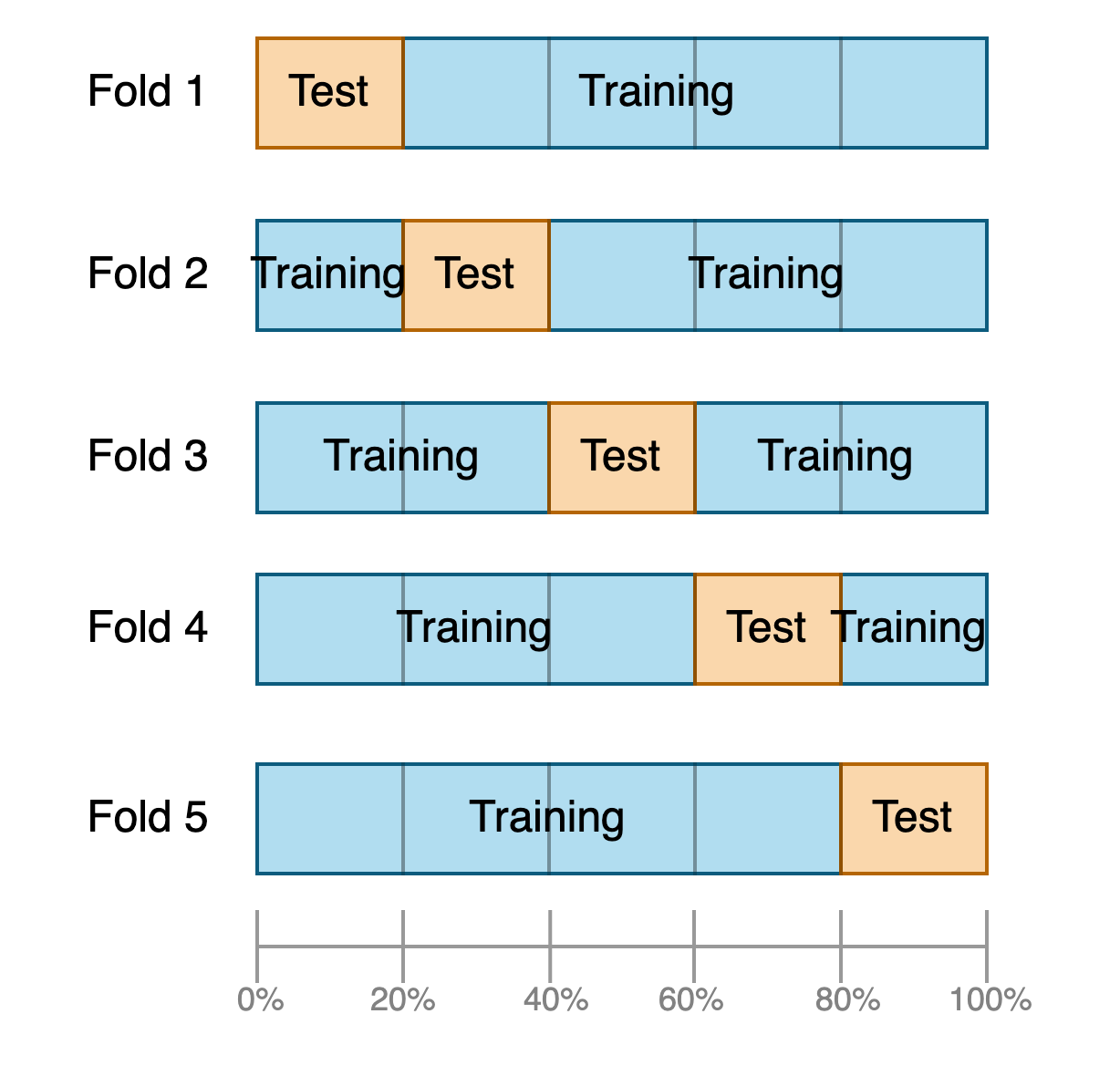

Supplement: S3 Fig — (DOCX) [file pdig.0000926.s003.docx]
