# Supplementary material for: Radiomics analysis for the early diagnosis of common sexually transmitted infections and skin lesions
Source: PLOS Digit Health. 2025 Jul 23;4(7):e0000926. doi: 10.1371/journal.pdig.0000926 (PMC12286352; doi:10.1371/journal.pdig.0000926)

S2 Fig. Nine different filters. (A) Original grey filter, (B) Gaussian Laplace (LoG) filter, (C) gradient filter, (D) square filter, (E) square root filter, (F) logarithm filter, (G) exponential filter,(H) two dimension local binary pattern (LBP2D), (I) HH Wavelet filter (extract diagonal features), (J) HL Wavelet filter (extract vertical features), (K) LH Wavelet filter (extract horizontal features), (L) LL Wavelet filter (the approximate image).


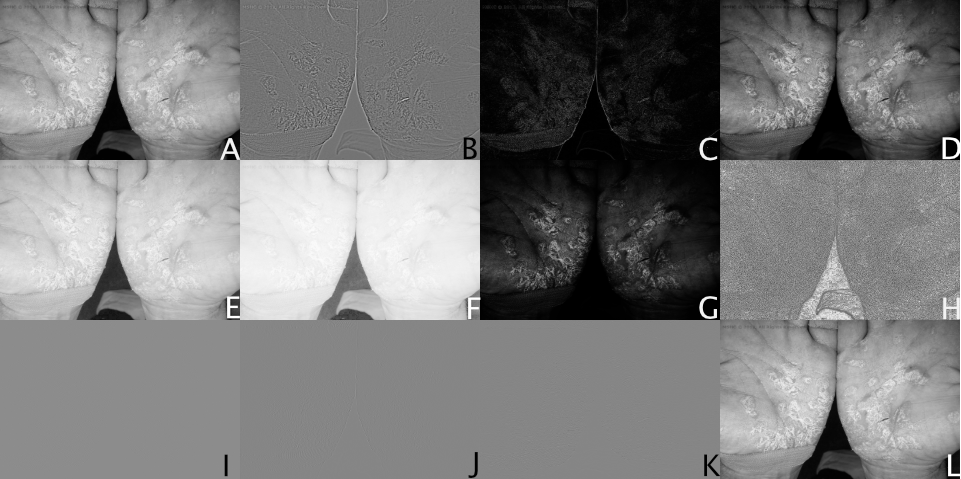

Supplement: S2 Fig — (A) Original grey filter, (B) Gaussian Laplace (LoG) filter, (C) gradient filter, (D) square filter, (E) square root filter, (F) logarithm filter, (G) exponential filter,(H) two dimension local binary pattern (LBP2D), (I) HH Wavelet filter (extract diagonal features), (J) HL Wavelet filter (extract vertical features), (K) LH Wavelet filter (extract horizontal features), (L) LL Wavelet filter (the approximate image). (DOCX) [file pdig.0000926.s002.docx]
