# Supplementary material for: Radiomics analysis for the early diagnosis of common sexually transmitted infections and skin lesions
Source: PLOS Digit Health. 2025 Jul 23;4(7):e0000926. doi: 10.1371/journal.pdig.0000926 (PMC12286352; doi:10.1371/journal.pdig.0000926)
Supplement: S1 Table — (DOCX) [file pdig.0000926.s004.docx]

S1 Table. General information about the STIs images dataset.

| **Infection class** | **Infection type** | **Infection site** | **Images num** |
| --- | --- | --- | --- |
| Sexually transmitted infections caused skin lesions | Herpes | Genitals | 36 |
|  | (n=61) | Other skin | 25 |
|  | Molluscum Contagiosum | Genitals | 12 |
|  | (n=66) | Other skin | 54 |
|  | Early Syphilis | Genitals | 45 |
|  | (n=143) | Anus | 12 |
|  |  | Other skin | 86 |
|  | Warts | Genitals | 54 |
|  | (n=121) | Anus | 15 |
|  |  | Other skin | 52 |
| General skin lesions | Lichen Sclerosus | Genitals | 127 |
|  | (n=145) | Other skin | 18 |
|  | Tinea (n=61) | Other skin | 61 |
