# Supplementary material for: Radiomics analysis for the early diagnosis of common sexually transmitted infections and skin lesions
Source: PLOS Digit Health. 2025 Jul 23;4(7):e0000926. doi: 10.1371/journal.pdig.0000926 (PMC12286352; doi:10.1371/journal.pdig.0000926)
Supplement: S2 Table — (DOCX) [file pdig.0000926.s005.docx]

S2 Table. AUC results for ten classifiers with nine filters on unspecific sites.

| Model Name | Herpes | Lichen Sclerosus | Molluscum Contagiosum | Early  Syphilis | Tinea | Warts | Total Average |
| --- | --- | --- | --- | --- | --- | --- | --- |
| LogisticRegression with Original filter | 0.530±0.070 | 0.708±0.047 | 0.730±0.036 | 0.506±0.041 | 0.642±0.087 | 0.592±0.033 | 0.618±0.052 |
| LogisticRegression with LoG filter | 0.566±0.084 | 0.771±0.029 | 0.740±0.102 | 0.592±0.054 | 0.719±0.057 | 0.620±0.059 | 0.668±0.064 |
| LogisticRegression with Gradient filter | 0.532±0.058 | 0.714±0.055 | 0.624±0.055 | 0.530±0.049 | 0.615±0.071 | 0.656±0.038 | 0.612±0.054 |
| LogisticRegression with Square filter | 0.547±0.071 | 0.714±0.065 | 0.693±0.069 | 0.526±0.039 | 0.621±0.039 | 0.601±0.073 | 0.617±0.059 |
| LogisticRegression with SquareRoot filter | 0.531±0.069 | 0.700±0.051 | 0.729±0.063 | 0.512±0.014 | 0.619±0.070 | 0.581±0.050 | 0.612±0.053 |
| LogisticRegression with Logarithm filter | 0.542±0.082 | 0.715±0.029 | 0.685±0.052 | 0.546±0.042 | 0.598±0.092 | 0.613±0.066 | 0.616±0.060 |
| LogisticRegression with Exponential filter | 0.567±0.059 | 0.750±0.045 | 0.716±0.088 | 0.548±0.035 | 0.656±0.090 | 0.641±0.059 | 0.646±0.063 |
| LogisticRegression with LBP2D filter | 0.533±0.080 | 0.725±0.048 | 0.609±0.046 | 0.577±0.049 | 0.560±0.039 | 0.534±0.079 | 0.590±0.057 |
| LogisticRegression with Wavelet filter | 0.583±0.059 | 0.796±0.047 | 0.677±0.064 | 0.589±0.084 | 0.686±0.088 | 0.626±0.085 | 0.660±0.071 |
| GBDT with Original filter | 0.548±0.031 | 0.714±0.033 | 0.668±0.075 | 0.573±0.035 | 0.619±0.064 | 0.639±0.076 | 0.627±0.052 |
| GBDT with LoG filter | 0.612±0.068 | 0.768±0.028 | 0.751±0.050 | 0.641±0.058 | 0.622±0.077 | 0.691±0.035 | 0.681±0.053 |
| GBDT with Gradient filter | 0.526±0.061 | 0.737±0.024 | 0.648±0.072 | 0.618±0.058 | 0.604±0.058 | 0.634±0.058 | 0.628±0.055 |
| GBDT with Square filter | 0.593±0.050 | 0.770±0.081 | 0.679±0.045 | 0.600±0.056 | 0.603±0.123 | 0.645±0.058 | 0.648±0.069 |
| GBDT with SquareRoot filter | 0.570±0.087 | 0.697±0.072 | 0.666±0.084 | 0.576±0.058 | 0.621±0.079 | 0.682±0.048 | 0.636±0.071 |
| GBDT with Logarithm filter | 0.581±0.071 | 0.719±0.056 | 0.699±0.079 | 0.567±0.062 | 0.581±0.063 | 0.626±0.043 | 0.629±0.062 |
| GBDT with Exponential filter | 0.575±0.047 | 0.696±0.036 | 0.640±0.122 | 0.527±0.054 | 0.631±0.080 | 0.643±0.059 | 0.619±0.066 |
| GBDT with LBP2D filter | 0.539±0.072 | 0.695±0.071 | 0.617±0.051 | 0.568±0.072 | 0.605±0.072 | 0.616±0.031 | 0.607±0.062 |
| GBDT with Wavelet filter | 0.570±0.100 | 0.787±0.040 | 0.674±0.048 | 0.608±0.033 | 0.705±0.120 | 0.635±0.040 | 0.663±0.064 |
| RidgeClassifier with Original filter | 0.529±0.053 | 0.694±0.086 | 0.698±0.039 | 0.515±0.038 | 0.551±0.020 | 0.600±0.068 | 0.598±0.051 |
| RidgeClassifier with LoG filter | 0.570±0.094 | 0.769±0.042 | 0.739±0.085 | 0.606±0.051 | 0.715±0.063 | 0.657±0.051 | 0.676±0.064 |
| RidgeClassifier with Gradient filter | 0.515±0.031 | 0.685±0.062 | 0.633±0.067 | 0.516±0.034 | 0.596±0.076 | 0.658±0.046 | 0.600±0.053 |
| RidgeClassifier with Square filter | 0.519±0.046 | 0.693±0.075 | 0.686±0.048 | 0.522±0.034 | 0.560±0.046 | 0.577±0.084 | 0.593±0.055 |
| RidgeClassifier with SquareRoot filter | 0.534±0.055 | 0.700±0.044 | 0.714±0.086 | 0.514±0.037 | 0.603±0.053 | 0.616±0.054 | 0.613±0.055 |
| RidgeClassifier with Logarithm filter | 0.541±0.069 | 0.708±0.022 | 0.669±0.040 | 0.533±0.045 | 0.559±0.066 | 0.625±0.065 | 0.606±0.051 |
| RidgeClassifier with Exponential filter | 0.569±0.069 | 0.722±0.094 | 0.694±0.073 | 0.576±0.039 | 0.578±0.032 | 0.644±0.101 | 0.630±0.068 |
| RidgeClassifier with LBP2D filter | 0.540±0.064 | 0.744±0.053 | 0.593±0.051 | 0.557±0.061 | 0.512±0.047 | 0.559±0.025 | 0.584±0.050 |
| RidgeClassifier with Wavelet filter | 0.556±0.047 | 0.810±0.050 | 0.727±0.059 | 0.573±0.070 | 0.656±0.060 | 0.645±0.046 | 0.661±0.055 |
| SVM with Original filter | 0.531±0.043 | 0.726±0.044 | 0.664±0.022 | 0.542±0.050 | 0.589±0.041 | 0.661±0.085 | 0.619±0.048 |
| SVM with LoG filter | 0.511±0.032 | 0.760±0.045 | 0.691±0.112 | 0.589±0.032 | 0.633±0.122 | 0.670±0.069 | 0.642±0.069 |
| SVM with Gradient filter | 0.507±0.021 | 0.697±0.052 | 0.630±0.053 | 0.532±0.053 | 0.560±0.047 | 0.650±0.059 | 0.596±0.047 |
| SVM with Square filter | 0.514±0.024 | 0.726±0.061 | 0.634±0.068 | 0.562±0.064 | 0.575±0.063 | 0.649±0.085 | 0.610±0.061 |
| SVM with SquareRoot filter | 0.515±0.028 | 0.742±0.063 | 0.643±0.051 | 0.551±0.053 | 0.617±0.059 | 0.686±0.067 | 0.626±0.053 |
| SVM with Logarithm filter | 0.523±0.029 | 0.743±0.054 | 0.621±0.045 | 0.522±0.041 | 0.538±0.037 | 0.658±0.056 | 0.601±0.044 |
| SVM with Exponential filter | 0.522±0.028 | 0.702±0.048 | 0.626±0.073 | 0.584±0.063 | 0.560±0.080 | 0.645±0.053 | 0.606±0.057 |
| SVM with LBP2D filter | 0.538±0.052 | 0.745±0.050 | 0.612±0.062 | 0.558±0.082 | 0.553±0.062 | 0.623±0.037 | 0.605±0.058 |
| SVM with Wavelet filter | 0.507±0.027 | 0.774±0.064 | 0.656±0.027 | 0.612±0.029 | 0.655±0.110 | 0.681±0.058 | 0.648±0.052 |
| KNN with Original filter | 0.569±0.052 | 0.727±0.060 | 0.626±0.093 | 0.566±0.020 | 0.641±0.054 | 0.652±0.025 | 0.630±0.051 |
| KNN with LoG filter | 0.566±0.069 | 0.703±0.047 | 0.658±0.094 | 0.599±0.053 | 0.591±0.045 | 0.657±0.055 | 0.629±0.060 |
| KNN with Gradient filter | 0.588±0.065 | 0.667±0.045 | 0.712±0.077 | 0.536±0.065 | 0.536±0.056 | 0.583±0.053 | 0.604±0.060 |
| KNN with Square filter | 0.531±0.046 | 0.676±0.069 | 0.652±0.088 | 0.528±0.040 | 0.658±0.119 | 0.620±0.045 | 0.611±0.068 |
| KNN with SquareRoot filter | 0.519±0.046 | 0.722±0.066 | 0.694±0.066 | 0.569±0.062 | 0.643±0.045 | 0.630±0.038 | 0.630±0.054 |
| KNN with Logarithm filter | 0.476±0.057 | 0.661±0.054 | 0.642±0.056 | 0.537±0.027 | 0.572±0.036 | 0.592±0.042 | 0.580±0.045 |
| KNN with Exponential filter | 0.566±0.039 | 0.693±0.089 | 0.687±0.091 | 0.589±0.064 | 0.581±0.107 | 0.635±0.057 | 0.625±0.075 |
| KNN with LBP2D filter | 0.593±0.112 | 0.705±0.030 | 0.598±0.094 | 0.546±0.024 | 0.618±0.064 | 0.608±0.034 | 0.611±0.060 |
| KNN with Wavelet filter | 0.627±0.046 | 0.713±0.072 | 0.668±0.090 | 0.545±0.049 | 0.571±0.040 | 0.623±0.045 | 0.625±0.057 |
| GaussianProcessClassifier with Original filter | 0.539±0.049 | 0.673±0.122 | 0.701±0.150 | 0.545±0.042 | 0.584±0.151 | 0.679±0.131 | 0.620±0.107 |
| GaussianProcessClassifier with LoG filter | 0.531±0.083 | 0.767±0.029 | 0.710±0.104 | 0.596±0.046 | 0.687±0.052 | 0.673±0.068 | 0.661±0.064 |
| GaussianProcessClassifier with Gradient filter | 0.499±0.003 | 0.716±0.044 | 0.672±0.047 | 0.519±0.042 | 0.605±0.079 | 0.662±0.082 | 0.612±0.049 |
| GaussianProcessClassifier with Square filter | 0.540±0.058 | 0.696±0.139 | 0.679±0.141 | 0.515±0.037 | 0.664±0.129 | 0.628±0.114 | 0.620±0.103 |
| GaussianProcessClassifier with SquareRoot filter | 0.555±0.083 | 0.732±0.052 | 0.714±0.050 | 0.542±0.034 | 0.606±0.091 | 0.723±0.064 | 0.645±0.062 |
| GaussianProcessClassifier with Logarithm filter | 0.527±0.045 | 0.733±0.043 | 0.710±0.030 | 0.553±0.055 | 0.526±0.052 | 0.698±0.059 | 0.624±0.047 |
| GaussianProcessClassifier with Exponential filter | 0.563±0.069 | 0.743±0.057 | 0.671±0.051 | 0.567±0.047 | 0.609±0.110 | 0.657±0.073 | 0.635±0.068 |
| GaussianProcessClassifier with LBP2D filter | 0.534±0.051 | 0.755±0.064 | 0.664±0.051 | 0.551±0.025 | 0.569±0.029 | 0.591±0.078 | 0.611±0.050 |
| GaussianProcessClassifier with Wavelet filter | 0.568±0.069 | 0.808±0.046 | 0.717±0.076 | 0.572±0.012 | 0.681±0.155 | 0.643±0.054 | 0.665±0.069 |
| DecisionTreeClassifier with Original filter | 0.552±0.081 | 0.676±0.060 | 0.646±0.088 | 0.568±0.070 | 0.543±0.060 | 0.635±0.050 | 0.603±0.068 |
| DecisionTreeClassifier with LoG filter | 0.541±0.054 | 0.705±0.058 | 0.633±0.113 | 0.562±0.042 | 0.594±0.100 | 0.631±0.072 | 0.611±0.073 |
| DecisionTreeClassifier with Gradient filter | 0.507±0.034 | 0.678±0.062 | 0.680±0.054 | 0.524±0.027 | 0.524±0.061 | 0.655±0.030 | 0.595±0.045 |
| DecisionTreeClassifier with Square filter | 0.535±0.035 | 0.687±0.100 | 0.555±0.057 | 0.538±0.097 | 0.533±0.041 | 0.594±0.095 | 0.574±0.071 |
| DecisionTreeClassifier with SquareRoot filter | 0.519±0.028 | 0.666±0.077 | 0.617±0.084 | 0.524±0.051 | 0.520±0.031 | 0.638±0.060 | 0.581±0.055 |
| DecisionTreeClassifier with Logarithm filter | 0.494±0.046 | 0.635±0.043 | 0.592±0.070 | 0.547±0.077 | 0.554±0.074 | 0.553±0.070 | 0.562±0.063 |
| DecisionTreeClassifier with Exponential filter | 0.517±0.051 | 0.672±0.077 | 0.568±0.035 | 0.536±0.052 | 0.576±0.079 | 0.583±0.071 | 0.575±0.061 |
| DecisionTreeClassifier with LBP2D filter | 0.531±0.074 | 0.709±0.062 | 0.680±0.046 | 0.558±0.092 | 0.550±0.067 | 0.615±0.048 | 0.607±0.065 |
| DecisionTreeClassifier with Wavelet filter | 0.532±0.038 | 0.675±0.081 | 0.603±0.093 | 0.559±0.072 | 0.564±0.094 | 0.595±0.042 | 0.588±0.070 |
| RandomForestClassifier with Original filter | 0.516±0.038 | 0.682±0.051 | 0.656±0.067 | 0.569±0.027 | 0.517±0.041 | 0.617±0.082 | 0.593±0.051 |
| RandomForestClassifier with LoG filter | 0.506±0.035 | 0.740±0.040 | 0.602±0.105 | 0.559±0.041 | 0.544±0.095 | 0.649±0.046 | 0.600±0.060 |
| RandomForestClassifier with Gradient filter | 0.494±0.006 | 0.699±0.098 | 0.647±0.080 | 0.534±0.094 | 0.506±0.047 | 0.656±0.023 | 0.590±0.058 |
| RandomForestClassifier with Square filter | 0.524±0.045 | 0.688±0.090 | 0.603±0.100 | 0.559±0.117 | 0.534±0.061 | 0.622±0.055 | 0.588±0.078 |
| RandomForestClassifier with SquareRoot filter | 0.506±0.038 | 0.714±0.049 | 0.633±0.056 | 0.588±0.060 | 0.542±0.038 | 0.635±0.097 | 0.603±0.056 |
| RandomForestClassifier with Logarithm filter | 0.507±0.040 | 0.707±0.063 | 0.622±0.122 | 0.567±0.071 | 0.495±0.007 | 0.638±0.053 | 0.589±0.059 |
| RandomForestClassifier with Exponential filter | 0.522±0.043 | 0.694±0.070 | 0.564±0.041 | 0.531±0.082 | 0.509±0.029 | 0.604±0.083 | 0.571±0.058 |
| RandomForestClassifier with LBP2D filter | 0.546±0.056 | 0.735±0.054 | 0.604±0.037 | 0.531±0.045 | 0.518±0.022 | 0.619±0.099 | 0.592±0.052 |
| RandomForestClassifier with Wavelet filter | 0.504±0.031 | 0.754±0.104 | 0.623±0.064 | 0.562±0.037 | 0.575±0.057 | 0.600±0.114 | 0.603±0.068 |
| MLPClassifier with Original filter | 0.560±0.078 | 0.717±0.014 | 0.713±0.105 | 0.590±0.045 | 0.633±0.114 | 0.695±0.076 | 0.651±0.072 |
| MLPClassifier with LoG filter | 0.569±0.099 | 0.761±0.051 | 0.697±0.047 | 0.637±0.073 | 0.690±0.042 | 0.660±0.063 | 0.669±0.063 |
| MLPClassifier with Gradient filter | 0.483±0.042 | 0.681±0.063 | 0.680±0.039 | 0.543±0.071 | 0.604±0.086 | 0.659±0.060 | 0.608±0.060 |
| MLPClassifier with Square filter | 0.549±0.066 | 0.710±0.059 | 0.685±0.076 | 0.578±0.066 | 0.627±0.132 | 0.654±0.072 | 0.634±0.079 |
| MLPClassifier with SquareRoot filter | 0.498±0.081 | 0.693±0.063 | 0.641±0.083 | 0.567±0.047 | 0.634±0.068 | 0.673±0.058 | 0.618±0.067 |
| MLPClassifier with Logarithm filter | 0.511±0.065 | 0.709±0.043 | 0.666±0.072 | 0.576±0.042 | 0.577±0.087 | 0.623±0.072 | 0.610±0.063 |
| MLPClassifier with Exponential filter | 0.577±0.062 | 0.718±0.054 | 0.671±0.079 | 0.542±0.063 | 0.620±0.075 | 0.635±0.038 | 0.627±0.062 |
| MLPClassifier with LBP2D filter | 0.567±0.114 | 0.694±0.090 | 0.652±0.041 | 0.544±0.046 | 0.596±0.078 | 0.567±0.056 | 0.603±0.071 |
| MLPClassifier with Wavelet filter | 0.536±0.076 | 0.778±0.084 | 0.664±0.083 | 0.603±0.055 | 0.683±0.120 | 0.662±0.093 | 0.654±0.085 |
| AdaBoostClassifier with Original filter | 0.552±0.113 | 0.638±0.096 | 0.645±0.044 | 0.527±0.071 | 0.573±0.090 | 0.516±0.071 | 0.575±0.081 |
| AdaBoostClassifier with LoG filter | 0.572±0.118 | 0.674±0.114 | 0.637±0.108 | 0.521±0.077 | 0.569±0.114 | 0.592±0.067 | 0.594±0.100 |
| AdaBoostClassifier with Gradient filter | 0.581±0.054 | 0.659±0.085 | 0.657±0.141 | 0.508±0.044 | 0.592±0.110 | 0.549±0.040 | 0.591±0.079 |
| AdaBoostClassifier with Square filter | 0.535±0.075 | 0.673±0.060 | 0.641±0.140 | 0.577±0.125 | 0.530±0.050 | 0.585±0.041 | 0.590±0.082 |
| AdaBoostClassifier with SquareRoot filter | 0.510±0.046 | 0.634±0.105 | 0.649±0.080 | 0.495±0.054 | 0.546±0.094 | 0.551±0.035 | 0.564±0.069 |
| AdaBoostClassifier with Logarithm filter | 0.510±0.097 | 0.622±0.022 | 0.593±0.076 | 0.518±0.025 | 0.531±0.097 | 0.569±0.064 | 0.557±0.063 |
| AdaBoostClassifier with Exponential filter | 0.512±0.060 | 0.641±0.024 | 0.653±0.070 | 0.524±0.098 | 0.602±0.061 | 0.620±0.059 | 0.592±0.062 |
| AdaBoostClassifier with LBP2D filter | 0.564±0.058 | 0.640±0.106 | 0.676±0.161 | 0.523±0.050 | 0.535±0.043 | 0.544±0.048 | 0.580±0.078 |
| AdaBoostClassifier with Wavelet filter | 0.585±0.075 | 0.665±0.078 | 0.648±0.107 | 0.570±0.038 | 0.619±0.141 | 0.566±0.053 | 0.609±0.082 |
| GaussianNB with Original filter | 0.598±0.074 | 0.700±0.077 | 0.690±0.048 | 0.531±0.034 | 0.618±0.093 | 0.529±0.008 | 0.611±0.056 |
| GaussianNB with LoG filter | 0.632±0.083 | 0.710±0.059 | 0.737±0.112 | 0.541±0.035 | 0.664±0.042 | 0.596±0.041 | 0.647±0.062 |
| GaussianNB with Gradient filter | 0.608±0.064 | 0.696±0.078 | 0.727±0.111 | 0.533±0.044 | 0.536±0.056 | 0.522±0.026 | 0.604±0.063 |
| GaussianNB with Square filter | 0.588±0.081 | 0.717±0.050 | 0.739±0.091 | 0.544±0.022 | 0.496±0.011 | 0.510±0.057 | 0.599±0.052 |
| GaussianNB with SquareRoot filter | 0.661±0.096 | 0.738±0.082 | 0.688±0.099 | 0.517±0.028 | 0.631±0.053 | 0.553±0.059 | 0.631±0.069 |
| GaussianNB with Logarithm filter | 0.572±0.066 | 0.713±0.068 | 0.642±0.049 | 0.520±0.027 | 0.590±0.076 | 0.520±0.030 | 0.593±0.052 |
| GaussianNB with Exponential filter | 0.631±0.081 | 0.557±0.058 | 0.675±0.065 | 0.536±0.017 | 0.639±0.049 | 0.546±0.042 | 0.597±0.052 |
| GaussianNB with LBP2D filter | 0.617±0.116 | 0.734±0.053 | 0.711±0.073 | 0.537±0.031 | 0.573±0.059 | 0.497±0.014 | 0.611±0.058 |
| GaussianNB with Wavelet filter | 0.558±0.055 | 0.716±0.034 | 0.712±0.087 | 0.529±0.022 | 0.731±0.068 | 0.560±0.037 | 0.634±0.051 |
