# Supplementary material for: Radiomics analysis for the early diagnosis of common sexually transmitted infections and skin lesions
Source: PLOS Digit Health. 2025 Jul 23;4(7):e0000926. doi: 10.1371/journal.pdig.0000926 (PMC12286352; doi:10.1371/journal.pdig.0000926)
Supplement: S3 Table — (DOCX) [file pdig.0000926.s006.docx]

S3 Table. Accuracy results for ten classifiers with nine filters on unspecific sites.

| **Model Name** | **Herpes** | **Lichen Sclerosus** | **Molluscum Contagiosum** | **Early Syphilis** | **Tinea** | **Warts** | **Total Average** |
| --- | --- | --- | --- | --- | --- | --- | --- |
| LogisticRegression with Original filter | 0.133±0.157 | 0.628±0.126 | 0.523±0.080 | 0.228±0.099 | 0.333±0.179 | 0.344±0.044 | 0.365±0.114 |
| LogisticRegression with LoG filter | 0.200±0.157 | 0.717±0.056 | 0.554±0.184 | 0.366±0.123 | 0.500±0.127 | 0.344±0.114 | 0.447±0.127 |
| LogisticRegression with Gradient filter | 0.100±0.113 | 0.669±0.116 | 0.308±0.117 | 0.255±0.089 | 0.267±0.153 | 0.504±0.067 | 0.350±0.109 |
| LogisticRegression with Square filter | 0.167±0.127 | 0.634±0.141 | 0.462±0.135 | 0.241±0.068 | 0.300±0.093 | 0.368±0.142 | 0.362±0.117 |
| LogisticRegression with SquareRoot filter | 0.117±0.139 | 0.628±0.126 | 0.523±0.105 | 0.248±0.082 | 0.283±0.139 | 0.328±0.065 | 0.354±0.109 |
| LogisticRegression with Logarithm filter | 0.133±0.173 | 0.634±0.099 | 0.431±0.109 | 0.331±0.123 | 0.250±0.146 | 0.376±0.103 | 0.359±0.126 |
| LogisticRegression with Exponential filter | 0.200±0.157 | 0.683±0.119 | 0.492±0.186 | 0.290±0.049 | 0.367±0.173 | 0.432±0.113 | 0.411±0.133 |
| LogisticRegression with LBP2D filter | 0.100±0.170 | 0.676±0.103 | 0.277±0.085 | 0.400±0.072 | 0.167±0.073 | 0.256±0.120 | 0.313±0.104 |
| LogisticRegression with Wavelet filter | 0.250±0.103 | 0.731±0.111 | 0.431±0.160 | 0.379±0.151 | 0.417±0.194 | 0.376±0.174 | 0.431±0.149 |
| GBDT with Original filter | 0.150±0.087 | 0.621±0.100 | 0.415±0.174 | 0.352±0.056 | 0.300±0.118 | 0.416±0.103 | 0.376±0.106 |
| GBDT with LoG filter | 0.267±0.135 | 0.710±0.072 | 0.554±0.105 | 0.448±0.096 | 0.300±0.157 | 0.512±0.054 | 0.465±0.103 |
| GBDT with Gradient filter | 0.133±0.118 | 0.648±0.082 | 0.354±0.128 | 0.462±0.141 | 0.250±0.146 | 0.400±0.116 | 0.375±0.122 |
| GBDT with Square filter | 0.250±0.103 | 0.683±0.170 | 0.446±0.105 | 0.400±0.137 | 0.267±0.247 | 0.416±0.090 | 0.410±0.142 |
| GBDT with SquareRoot filter | 0.200±0.188 | 0.586±0.171 | 0.400±0.157 | 0.379±0.096 | 0.283±0.157 | 0.496±0.057 | 0.391±0.138 |
| GBDT with Logarithm filter | 0.217±0.139 | 0.621±0.135 | 0.477±0.157 | 0.366±0.089 | 0.200±0.118 | 0.400±0.061 | 0.380±0.116 |
| GBDT with Exponential filter | 0.200±0.118 | 0.579±0.111 | 0.354±0.220 | 0.290±0.123 | 0.333±0.164 | 0.432±0.133 | 0.365±0.145 |
| GBDT with LBP2D filter | 0.133±0.139 | 0.566±0.195 | 0.292±0.105 | 0.400±0.181 | 0.267±0.153 | 0.392±0.108 | 0.342±0.147 |
| GBDT with Wavelet filter | 0.217±0.215 | 0.697±0.088 | 0.415±0.085 | 0.414±0.030 | 0.467±0.238 | 0.408±0.042 | 0.436±0.116 |
| RidgeClassifier with Original filter | 0.083±0.103 | 0.676±0.137 | 0.446±0.080 | 0.241±0.068 | 0.133±0.057 | 0.392±0.108 | 0.329±0.092 |
| RidgeClassifier with LoG filter | 0.183±0.170 | 0.786±0.077 | 0.538±0.135 | 0.359±0.072 | 0.467±0.118 | 0.424±0.090 | 0.460±0.110 |
| RidgeClassifier with Gradient filter | 0.033±0.057 | 0.676±0.141 | 0.323±0.125 | 0.214±0.047 | 0.217±0.157 | 0.528±0.065 | 0.332±0.098 |
| RidgeClassifier with Square filter | 0.083±0.103 | 0.690±0.148 | 0.446±0.080 | 0.193±0.065 | 0.150±0.113 | 0.360±0.161 | 0.320±0.112 |
| RidgeClassifier with SquareRoot filter | 0.083±0.103 | 0.655±0.100 | 0.508±0.160 | 0.248±0.036 | 0.233±0.113 | 0.408±0.054 | 0.356±0.095 |
| RidgeClassifier with Logarithm filter | 0.100±0.135 | 0.683±0.107 | 0.400±0.080 | 0.297±0.099 | 0.150±0.113 | 0.416±0.090 | 0.341±0.104 |
| RidgeClassifier with Exponential filter | 0.167±0.164 | 0.710±0.181 | 0.446±0.125 | 0.303±0.019 | 0.183±0.046 | 0.480±0.145 | 0.382±0.113 |
| RidgeClassifier with LBP2D filter | 0.100±0.135 | 0.766±0.064 | 0.246±0.105 | 0.345±0.100 | 0.033±0.093 | 0.320±0.000 | 0.302±0.083 |
| RidgeClassifier with Wavelet filter | 0.167±0.103 | 0.800±0.077 | 0.523±0.125 | 0.331±0.123 | 0.350±0.135 | 0.424±0.083 | 0.432±0.108 |
| SVM with Original filter | 0.067±0.087 | 0.655±0.100 | 0.369±0.043 | 0.359±0.123 | 0.200±0.093 | 0.520±0.079 | 0.362±0.087 |
| SVM with LoG filter | 0.050±0.057 | 0.752±0.111 | 0.415±0.230 | 0.386±0.098 | 0.300±0.260 | 0.496±0.143 | 0.400±0.150 |
| SVM with Gradient filter | 0.017±0.046 | 0.634±0.144 | 0.308±0.117 | 0.303±0.070 | 0.133±0.093 | 0.544±0.103 | 0.323±0.096 |
| SVM with Square filter | 0.033±0.057 | 0.669±0.137 | 0.323±0.125 | 0.393±0.165 | 0.167±0.127 | 0.488±0.129 | 0.346±0.123 |
| SVM with SquareRoot filter | 0.033±0.057 | 0.669±0.144 | 0.338±0.085 | 0.372±0.150 | 0.250±0.127 | 0.568±0.065 | 0.372±0.105 |
| SVM with Logarithm filter | 0.050±0.057 | 0.690±0.109 | 0.292±0.080 | 0.359±0.116 | 0.083±0.073 | 0.504±0.083 | 0.330±0.086 |
| SVM with Exponential filter | 0.050±0.057 | 0.655±0.100 | 0.292±0.171 | 0.400±0.141 | 0.133±0.157 | 0.504±0.067 | 0.339±0.115 |
| SVM with LBP2D filter | 0.083±0.103 | 0.697±0.130 | 0.277±0.145 | 0.379±0.109 | 0.133±0.139 | 0.448±0.054 | 0.336±0.113 |
| SVM with Wavelet filter | 0.033±0.057 | 0.683±0.126 | 0.354±0.052 | 0.503±0.078 | 0.333±0.219 | 0.536±0.057 | 0.407±0.098 |
| KNN with Original filter | 0.283±0.118 | 0.731±0.070 | 0.323±0.184 | 0.248±0.047 | 0.317±0.113 | 0.384±0.027 | 0.381±0.093 |
| KNN with LoG filter | 0.283±0.139 | 0.676±0.099 | 0.369±0.171 | 0.317±0.098 | 0.233±0.087 | 0.392±0.108 | 0.379±0.117 |
| KNN with Gradient filter | 0.333±0.164 | 0.621±0.068 | 0.508±0.145 | 0.200±0.111 | 0.100±0.113 | 0.280±0.127 | 0.340±0.121 |
| KNN with Square filter | 0.217±0.057 | 0.662±0.082 | 0.354±0.160 | 0.193±0.089 | 0.350±0.224 | 0.336±0.090 | 0.352±0.117 |
| KNN with SquareRoot filter | 0.200±0.057 | 0.710±0.083 | 0.462±0.135 | 0.262±0.116 | 0.317±0.087 | 0.336±0.083 | 0.381±0.093 |
| KNN with Logarithm filter | 0.150±0.087 | 0.607±0.083 | 0.369±0.080 | 0.228±0.057 | 0.167±0.073 | 0.264±0.057 | 0.297±0.073 |
| KNN with Exponential filter | 0.267±0.087 | 0.669±0.150 | 0.431±0.209 | 0.290±0.131 | 0.200±0.202 | 0.384±0.114 | 0.373±0.149 |
| KNN with LBP2D filter | 0.333±0.231 | 0.621±0.105 | 0.277±0.198 | 0.255±0.065 | 0.300±0.118 | 0.320±0.061 | 0.351±0.130 |
| KNN with Wavelet filter | 0.450±0.093 | 0.676±0.089 | 0.415±0.160 | 0.214±0.107 | 0.183±0.087 | 0.304±0.083 | 0.374±0.103 |
| GaussianProcessClassifier with Original filter | 0.083±0.103 | 0.531±0.370 | 0.462±0.338 | 0.207±0.169 | 0.383±0.526 | 0.528±0.374 | 0.366±0.313 |
| GaussianProcessClassifier with LoG filter | 0.100±0.170 | 0.786±0.093 | 0.492±0.186 | 0.324±0.072 | 0.417±0.127 | 0.472±0.129 | 0.432±0.129 |
| GaussianProcessClassifier with Gradient filter | 0.000±0.000 | 0.697±0.070 | 0.415±0.109 | 0.186±0.038 | 0.250±0.164 | 0.560±0.140 | 0.351±0.087 |
| GaussianProcessClassifier with Square filter | 0.100±0.135 | 0.572±0.405 | 0.431±0.328 | 0.131±0.119 | 0.567±0.338 | 0.408±0.305 | 0.368±0.272 |
| GaussianProcessClassifier with SquareRoot filter | 0.117±0.173 | 0.710±0.141 | 0.508±0.109 | 0.207±0.068 | 0.250±0.219 | 0.648±0.096 | 0.407±0.134 |
| GaussianProcessClassifier with Logarithm filter | 0.067±0.087 | 0.731±0.102 | 0.492±0.052 | 0.241±0.105 | 0.083±0.127 | 0.600±0.111 | 0.369±0.097 |
| GaussianProcessClassifier with Exponential filter | 0.150±0.153 | 0.745±0.134 | 0.400±0.125 | 0.276±0.109 | 0.250±0.219 | 0.512±0.102 | 0.389±0.140 |
| GaussianProcessClassifier with LBP2D filter | 0.083±0.103 | 0.745±0.137 | 0.400±0.105 | 0.276±0.030 | 0.167±0.073 | 0.416±0.139 | 0.348±0.098 |
| GaussianProcessClassifier with Wavelet filter | 0.167±0.146 | 0.814±0.103 | 0.492±0.160 | 0.283±0.064 | 0.417±0.310 | 0.464±0.130 | 0.439±0.152 |
| DecisionTreeClassifier with Original filter | 0.167±0.164 | 0.517±0.096 | 0.338±0.174 | 0.379±0.184 | 0.100±0.135 | 0.512±0.133 | 0.336±0.148 |
| DecisionTreeClassifier with LoG filter | 0.117±0.118 | 0.586±0.052 | 0.338±0.209 | 0.434±0.083 | 0.233±0.224 | 0.384±0.188 | 0.349±0.146 |
| DecisionTreeClassifier with Gradient filter | 0.033±0.093 | 0.517±0.151 | 0.431±0.109 | 0.241±0.174 | 0.083±0.103 | 0.624±0.167 | 0.322±0.133 |
| DecisionTreeClassifier with Square filter | 0.150±0.135 | 0.566±0.191 | 0.169±0.184 | 0.317±0.153 | 0.117±0.093 | 0.392±0.301 | 0.285±0.176 |
| DecisionTreeClassifier with SquareRoot filter | 0.100±0.087 | 0.572±0.144 | 0.292±0.157 | 0.324±0.065 | 0.050±0.057 | 0.448±0.151 | 0.298±0.110 |
| DecisionTreeClassifier with Logarithm filter | 0.033±0.093 | 0.448±0.151 | 0.246±0.171 | 0.421±0.206 | 0.150±0.135 | 0.312±0.173 | 0.268±0.155 |
| DecisionTreeClassifier with Exponential filter | 0.083±0.103 | 0.503±0.153 | 0.185±0.085 | 0.290±0.127 | 0.233±0.185 | 0.440±0.186 | 0.289±0.140 |
| DecisionTreeClassifier with LBP2D filter | 0.117±0.118 | 0.614±0.133 | 0.415±0.109 | 0.324±0.200 | 0.167±0.164 | 0.416±0.151 | 0.342±0.146 |
| DecisionTreeClassifier with Wavelet filter | 0.100±0.087 | 0.510±0.130 | 0.262±0.220 | 0.414±0.179 | 0.200±0.227 | 0.376±0.134 | 0.310±0.163 |
| RandomForestClassifier with Original filter | 0.050±0.093 | 0.628±0.126 | 0.369±0.125 | 0.372±0.082 | 0.050±0.093 | 0.432±0.119 | 0.317±0.106 |
| RandomForestClassifier with LoG filter | 0.033±0.057 | 0.779±0.103 | 0.231±0.214 | 0.331±0.038 | 0.117±0.215 | 0.472±0.082 | 0.327±0.118 |
| RandomForestClassifier with Gradient filter | 0.000±0.000 | 0.655±0.184 | 0.338±0.145 | 0.262±0.137 | 0.033±0.093 | 0.576±0.027 | 0.311±0.098 |
| RandomForestClassifier with Square filter | 0.067±0.087 | 0.655±0.151 | 0.246±0.218 | 0.366±0.220 | 0.083±0.103 | 0.440±0.050 | 0.309±0.138 |
| RandomForestClassifier with SquareRoot filter | 0.033±0.093 | 0.683±0.082 | 0.308±0.135 | 0.400±0.094 | 0.100±0.087 | 0.472±0.170 | 0.333±0.110 |
| RandomForestClassifier with Logarithm filter | 0.033±0.093 | 0.648±0.123 | 0.292±0.238 | 0.441±0.146 | 0.000±0.000 | 0.440±0.127 | 0.309±0.121 |
| RandomForestClassifier with Exponential filter | 0.050±0.093 | 0.655±0.151 | 0.169±0.105 | 0.303±0.137 | 0.033±0.057 | 0.464±0.130 | 0.279±0.112 |
| RandomForestClassifier with LBP2D filter | 0.117±0.118 | 0.710±0.103 | 0.262±0.109 | 0.290±0.089 | 0.050±0.057 | 0.464±0.210 | 0.315±0.114 |
| RandomForestClassifier with Wavelet filter | 0.033±0.057 | 0.759±0.160 | 0.277±0.128 | 0.379±0.091 | 0.167±0.127 | 0.384±0.159 | 0.333±0.120 |
| MLPClassifier with Original filter | 0.217±0.173 | 0.559±0.047 | 0.492±0.220 | 0.379±0.061 | 0.333±0.264 | 0.520±0.157 | 0.417±0.154 |
| MLPClassifier with LoG filter | 0.217±0.173 | 0.655±0.091 | 0.462±0.096 | 0.441±0.161 | 0.450±0.057 | 0.448±0.124 | 0.445±0.117 |
| MLPClassifier with Gradient filter | 0.067±0.087 | 0.517±0.135 | 0.431±0.085 | 0.297±0.141 | 0.300±0.173 | 0.456±0.134 | 0.345±0.126 |
| MLPClassifier with Square filter | 0.200±0.139 | 0.566±0.134 | 0.446±0.184 | 0.359±0.162 | 0.317±0.278 | 0.440±0.149 | 0.388±0.174 |
| MLPClassifier with SquareRoot filter | 0.117±0.157 | 0.517±0.135 | 0.369±0.171 | 0.317±0.082 | 0.350±0.170 | 0.488±0.082 | 0.360±0.133 |
| MLPClassifier with Logarithm filter | 0.100±0.113 | 0.572±0.083 | 0.415±0.145 | 0.352±0.098 | 0.233±0.153 | 0.408±0.108 | 0.347±0.117 |
| MLPClassifier with Exponential filter | 0.267±0.135 | 0.572±0.137 | 0.431±0.145 | 0.290±0.094 | 0.317±0.170 | 0.392±0.065 | 0.378±0.124 |
| MLPClassifier with LBP2D filter | 0.200±0.238 | 0.559±0.190 | 0.400±0.080 | 0.310±0.086 | 0.283±0.157 | 0.280±0.086 | 0.339±0.139 |
| MLPClassifier with Wavelet filter | 0.167±0.164 | 0.655±0.163 | 0.415±0.160 | 0.379±0.125 | 0.433±0.236 | 0.472±0.166 | 0.420±0.169 |
| AdaBoostClassifier with Original filter | 0.233±0.236 | 0.483±0.199 | 0.354±0.109 | 0.290±0.116 | 0.217±0.202 | 0.184±0.103 | 0.293±0.161 |
| AdaBoostClassifier with LoG filter | 0.250±0.219 | 0.552±0.205 | 0.308±0.224 | 0.303±0.161 | 0.200±0.215 | 0.328±0.108 | 0.323±0.189 |
| AdaBoostClassifier with Gradient filter | 0.267±0.087 | 0.497±0.123 | 0.369±0.290 | 0.283±0.070 | 0.250±0.243 | 0.256±0.075 | 0.320±0.148 |
| AdaBoostClassifier with Square filter | 0.167±0.164 | 0.531±0.183 | 0.338±0.291 | 0.393±0.226 | 0.117±0.139 | 0.336±0.109 | 0.314±0.185 |
| AdaBoostClassifier with SquareRoot filter | 0.133±0.093 | 0.455±0.199 | 0.431±0.145 | 0.234±0.170 | 0.183±0.199 | 0.208±0.054 | 0.274±0.143 |
| AdaBoostClassifier with Logarithm filter | 0.150±0.212 | 0.455±0.077 | 0.246±0.171 | 0.297±0.094 | 0.133±0.157 | 0.280±0.136 | 0.260±0.141 |
| AdaBoostClassifier with Exponential filter | 0.117±0.118 | 0.476±0.077 | 0.415±0.145 | 0.262±0.181 | 0.267±0.153 | 0.384±0.139 | 0.320±0.135 |
| AdaBoostClassifier with LBP2D filter | 0.200±0.093 | 0.483±0.199 | 0.446±0.347 | 0.234±0.070 | 0.150±0.046 | 0.296±0.109 | 0.302±0.144 |
| AdaBoostClassifier with Wavelet filter | 0.250±0.146 | 0.497±0.147 | 0.354±0.198 | 0.386±0.088 | 0.300±0.298 | 0.304±0.067 | 0.348±0.157 |
| GaussianNB with Original filter | 0.417±0.103 | 0.669±0.144 | 0.492±0.085 | 0.110±0.070 | 0.300±0.215 | 0.136±0.027 | 0.354±0.107 |
| GaussianNB with LoG filter | 0.433±0.135 | 0.662±0.102 | 0.631±0.238 | 0.097±0.064 | 0.417±0.073 | 0.248±0.082 | 0.415±0.116 |
| GaussianNB with Gradient filter | 0.533±0.139 | 0.559±0.153 | 0.662±0.230 | 0.097±0.093 | 0.100±0.135 | 0.096±0.075 | 0.341±0.137 |
| GaussianNB with Square filter | 0.417±0.179 | 0.621±0.117 | 0.646±0.198 | 0.145±0.036 | 0.017±0.046 | 0.144±0.075 | 0.332±0.109 |
| GaussianNB with SquareRoot filter | 0.600±0.153 | 0.607±0.173 | 0.523±0.171 | 0.083±0.038 | 0.350±0.135 | 0.168±0.113 | 0.388±0.131 |
| GaussianNB with Logarithm filter | 0.400±0.185 | 0.669±0.181 | 0.431±0.128 | 0.076±0.047 | 0.267±0.170 | 0.088±0.065 | 0.322±0.129 |
| GaussianNB with Exponential filter | 0.550±0.215 | 0.152±0.162 | 0.415±0.145 | 0.103±0.030 | 0.567±0.087 | 0.224±0.097 | 0.335±0.122 |
| GaussianNB with LBP2D filter | 0.450±0.227 | 0.752±0.082 | 0.554±0.196 | 0.145±0.047 | 0.200±0.157 | 0.024±0.027 | 0.354±0.123 |
| GaussianNB with Wavelet filter | 0.233±0.135 | 0.559±0.093 | 0.646±0.209 | 0.090±0.023 | 0.550±0.173 | 0.288±0.065 | 0.394±0.116 |
