# Supplementary material for: Radiomics analysis for the early diagnosis of common sexually transmitted infections and skin lesions
Source: PLOS Digit Health. 2025 Jul 23;4(7):e0000926. doi: 10.1371/journal.pdig.0000926 (PMC12286352; doi:10.1371/journal.pdig.0000926)
Supplement: S4 Table — (DOCX) [file pdig.0000926.s007.docx]

S4 Table. Specificity results for ten classifiers with nine filters on unspecific sites.

| **Model Name** | **Herpes** | **Lichen Sclerosus** | **Molluscum Contagiosum** | **Early Syphilis** | **Tinea** | **Warts** | **Total Average** |
| --- | --- | --- | --- | --- | --- | --- | --- |
| LogisticRegression with Original filter | 0.737±0.513 | 0.789±0.057 | 0.936±0.017 | 0.785±0.047 | 0.950±0.021 | 0.840±0.041 | 0.840±0.116 |
| LogisticRegression with LoG filter | 0.931±0.022 | 0.824±0.059 | 0.927±0.034 | 0.818±0.058 | 0.937±0.031 | 0.897±0.031 | 0.889±0.039 |
| LogisticRegression with Gradient filter | 0.580±0.657 | 0.758±0.088 | 0.940±0.027 | 0.804±0.074 | 0.963±0.022 | 0.808±0.028 | 0.809±0.149 |
| LogisticRegression with Square filter | 0.741±0.516 | 0.793±0.015 | 0.925±0.022 | 0.811±0.054 | 0.943±0.035 | 0.834±0.039 | 0.841±0.113 |
| LogisticRegression with SquareRoot filter | 0.569±0.645 | 0.771±0.074 | 0.935±0.025 | 0.776±0.062 | 0.954±0.026 | 0.834±0.052 | 0.806±0.147 |
| LogisticRegression with Logarithm filter | 0.569±0.645 | 0.796±0.056 | 0.938±0.010 | 0.760±0.049 | 0.946±0.051 | 0.851±0.036 | 0.810±0.141 |
| LogisticRegression with Exponential filter | 0.739±0.515 | 0.818±0.067 | 0.940±0.034 | 0.807±0.057 | 0.946±0.022 | 0.851±0.052 | 0.850±0.125 |
| LogisticRegression with LBP2D filter | 0.581±0.659 | 0.774±0.050 | 0.942±0.019 | 0.754±0.043 | 0.954±0.044 | 0.813±0.057 | 0.803±0.145 |
| LogisticRegression with Wavelet filter | 0.917±0.036 | 0.862±0.053 | 0.923±0.044 | 0.798±0.049 | 0.956±0.032 | 0.876±0.036 | 0.888±0.042 |
| GBDT with Original filter | 0.946±0.039 | 0.807±0.037 | 0.920±0.027 | 0.793±0.026 | 0.939±0.017 | 0.861±0.072 | 0.878±0.036 |
| GBDT with LoG filter | 0.957±0.021 | 0.826±0.024 | 0.948±0.023 | 0.833±0.024 | 0.944±0.035 | 0.869±0.038 | 0.896±0.028 |
| GBDT with Gradient filter | 0.739±0.515 | 0.826±0.052 | 0.942±0.025 | 0.774±0.052 | 0.957±0.033 | 0.867±0.007 | 0.851±0.114 |
| GBDT with Square filter | 0.935±0.033 | 0.857±0.049 | 0.912±0.028 | 0.800±0.054 | 0.939±0.046 | 0.874±0.035 | 0.886±0.041 |
| GBDT with SquareRoot filter | 0.752±0.522 | 0.809±0.053 | 0.933±0.015 | 0.774±0.068 | 0.959±0.024 | 0.867±0.057 | 0.849±0.123 |
| GBDT with Logarithm filter | 0.944±0.011 | 0.818±0.036 | 0.921±0.028 | 0.769±0.045 | 0.961±0.019 | 0.853±0.038 | 0.878±0.030 |
| GBDT with Exponential filter | 0.950±0.032 | 0.813±0.054 | 0.927±0.034 | 0.765±0.034 | 0.930±0.039 | 0.855±0.023 | 0.873±0.036 |
| GBDT with LBP2D filter | 0.756±0.525 | 0.824±0.075 | 0.942±0.022 | 0.736±0.057 | 0.943±0.036 | 0.840±0.048 | 0.840±0.127 |
| GBDT with Wavelet filter | 0.737±0.512 | 0.877±0.035 | 0.933±0.054 | 0.802±0.066 | 0.943±0.026 | 0.861±0.048 | 0.859±0.124 |
| RidgeClassifier with Original filter | 0.585±0.663 | 0.712±0.071 | 0.950±0.023 | 0.789±0.044 | 0.969±0.026 | 0.808±0.044 | 0.802±0.145 |
| RidgeClassifier with LoG filter | 0.957±0.031 | 0.752±0.052 | 0.940±0.039 | 0.853±0.049 | 0.963±0.026 | 0.891±0.040 | 0.893±0.039 |
| RidgeClassifier with Gradient filter | 0.400±0.680 | 0.695±0.086 | 0.942±0.040 | 0.818±0.058 | 0.976±0.013 | 0.787±0.053 | 0.770±0.155 |
| RidgeClassifier with Square filter | 0.569±0.645 | 0.697±0.023 | 0.925±0.026 | 0.851±0.031 | 0.772±0.537 | 0.794±0.027 | 0.768±0.215 |
| RidgeClassifier with SquareRoot filter | 0.594±0.674 | 0.745±0.063 | 0.920±0.019 | 0.780±0.055 | 0.972±0.027 | 0.823±0.068 | 0.806±0.151 |
| RidgeClassifier with Logarithm filter | 0.591±0.670 | 0.734±0.082 | 0.938±0.016 | 0.769±0.035 | 0.778±0.541 | 0.834±0.040 | 0.774±0.230 |
| RidgeClassifier with Exponential filter | 0.772±0.537 | 0.734±0.033 | 0.942±0.041 | 0.848±0.061 | 0.972±0.027 | 0.808±0.069 | 0.846±0.128 |
| RidgeClassifier with LBP2D filter | 0.587±0.665 | 0.723±0.072 | 0.940±0.021 | 0.769±0.040 | 0.198±0.550 | 0.798±0.049 | 0.669±0.233 |
| RidgeClassifier with Wavelet filter | 0.944±0.018 | 0.820±0.052 | 0.931±0.013 | 0.815±0.051 | 0.961±0.021 | 0.865±0.058 | 0.889±0.035 |
| SVM with Original filter | 0.594±0.674 | 0.798±0.043 | 0.959±0.019 | 0.725±0.051 | 0.978±0.013 | 0.802±0.096 | 0.809±0.149 |
| SVM with LoG filter | 0.585±0.663 | 0.769±0.057 | 0.966±0.027 | 0.791±0.059 | 0.967±0.032 | 0.844±0.080 | 0.820±0.153 |
| SVM with Gradient filter | 0.198±0.550 | 0.760±0.047 | 0.953±0.014 | 0.760±0.074 | 0.987±0.013 | 0.756±0.047 | 0.736±0.124 |
| SVM with Square filter | 0.394±0.671 | 0.782±0.038 | 0.946±0.028 | 0.732±0.049 | 0.983±0.017 | 0.811±0.076 | 0.775±0.146 |
| SVM with SquareRoot filter | 0.398±0.677 | 0.815±0.049 | 0.948±0.028 | 0.730±0.061 | 0.983±0.013 | 0.804±0.102 | 0.780±0.155 |
| SVM with Logarithm filter | 0.598±0.678 | 0.796±0.064 | 0.950±0.021 | 0.686±0.059 | 0.794±0.551 | 0.813±0.085 | 0.773±0.243 |
| SVM with Exponential filter | 0.596±0.676 | 0.749±0.053 | 0.959±0.028 | 0.767±0.039 | 0.789±0.548 | 0.785±0.054 | 0.774±0.233 |
| SVM with LBP2D filter | 0.596±0.676 | 0.793±0.067 | 0.948±0.029 | 0.736±0.082 | 0.774±0.538 | 0.798±0.063 | 0.774±0.243 |
| SVM with Wavelet filter | 0.391±0.664 | 0.866±0.030 | 0.959±0.006 | 0.721±0.044 | 0.976±0.006 | 0.825±0.098 | 0.790±0.141 |
| KNN with Original filter | 0.854±0.055 | 0.723±0.057 | 0.929±0.010 | 0.884±0.055 | 0.965±0.019 | 0.920±0.029 | 0.879±0.037 |
| KNN with LoG filter | 0.848±0.017 | 0.730±0.051 | 0.948±0.034 | 0.881±0.038 | 0.948±0.015 | 0.922±0.022 | 0.880±0.030 |
| KNN with Gradient filter | 0.843±0.059 | 0.714±0.055 | 0.916±0.022 | 0.873±0.028 | 0.776±0.539 | 0.886±0.041 | 0.835±0.124 |
| KNN with Square filter | 0.846±0.053 | 0.690±0.061 | 0.950±0.019 | 0.864±0.025 | 0.967±0.015 | 0.903±0.047 | 0.870±0.037 |
| KNN with SquareRoot filter | 0.839±0.058 | 0.734±0.051 | 0.927±0.031 | 0.877±0.011 | 0.969±0.013 | 0.924±0.036 | 0.878±0.033 |
| KNN with Logarithm filter | 0.802±0.032 | 0.714±0.043 | 0.914±0.044 | 0.846±0.033 | 0.978±0.017 | 0.920±0.049 | 0.862±0.036 |
| KNN with Exponential filter | 0.865±0.024 | 0.716±0.087 | 0.944±0.031 | 0.888±0.018 | 0.774±0.538 | 0.886±0.047 | 0.846±0.124 |
| KNN with LBP2D filter | 0.852±0.035 | 0.789±0.058 | 0.920±0.023 | 0.837±0.076 | 0.935±0.027 | 0.897±0.044 | 0.872±0.044 |
| KNN with Wavelet filter | 0.804±0.032 | 0.749±0.077 | 0.921±0.033 | 0.877±0.011 | 0.959±0.019 | 0.941±0.012 | 0.875±0.031 |
| GaussianProcessClassifier with Original filter | 0.594±0.674 | 0.615±0.429 | 0.740±0.514 | 0.684±0.476 | 0.385±0.655 | 0.629±0.438 | 0.608±0.531 |
| GaussianProcessClassifier with LoG filter | 0.383±0.652 | 0.747±0.055 | 0.927±0.040 | 0.868±0.062 | 0.957±0.037 | 0.874±0.024 | 0.793±0.145 |
| GaussianProcessClassifier with Gradient filter | 0.000±0.000 | 0.736±0.066 | 0.929±0.025 | 0.853±0.073 | 0.959±0.013 | 0.764±0.030 | 0.707±0.035 |
| GaussianProcessClassifier with Square filter | 0.583±0.662 | 0.620±0.435 | 0.727±0.505 | 0.699±0.486 | 0.761±0.530 | 0.648±0.452 | 0.673±0.512 |
| GaussianProcessClassifier with SquareRoot filter | 0.593±0.672 | 0.754±0.050 | 0.920±0.025 | 0.877±0.030 | 0.763±0.530 | 0.798±0.058 | 0.784±0.227 |
| GaussianProcessClassifier with Logarithm filter | 0.591±0.670 | 0.734±0.056 | 0.927±0.033 | 0.864±0.012 | 0.570±0.647 | 0.796±0.020 | 0.747±0.240 |
| GaussianProcessClassifier with Exponential filter | 0.780±0.542 | 0.741±0.065 | 0.942±0.028 | 0.857±0.059 | 0.769±0.534 | 0.802±0.047 | 0.815±0.212 |
| GaussianProcessClassifier with LBP2D filter | 0.591±0.670 | 0.765±0.052 | 0.927±0.036 | 0.826±0.035 | 0.972±0.030 | 0.766±0.035 | 0.808±0.143 |
| GaussianProcessClassifier with Wavelet filter | 0.774±0.538 | 0.802±0.055 | 0.942±0.019 | 0.862±0.050 | 0.944±0.034 | 0.821±0.068 | 0.858±0.127 |
| DecisionTreeClassifier with Original filter | 0.741±0.515 | 0.835±0.061 | 0.953±0.018 | 0.756±0.132 | 0.585±0.663 | 0.758±0.095 | 0.771±0.247 |
| DecisionTreeClassifier with LoG filter | 0.767±0.532 | 0.824±0.068 | 0.927±0.025 | 0.690±0.065 | 0.956±0.048 | 0.878±0.051 | 0.840±0.132 |
| DecisionTreeClassifier with Gradient filter | 0.189±0.524 | 0.840±0.040 | 0.929±0.021 | 0.807±0.140 | 0.587±0.666 | 0.686±0.112 | 0.673±0.251 |
| DecisionTreeClassifier with Square filter | 0.920±0.078 | 0.809±0.044 | 0.742±0.519 | 0.758±0.136 | 0.757±0.527 | 0.796±0.165 | 0.797±0.245 |
| DecisionTreeClassifier with SquareRoot filter | 0.741±0.517 | 0.760±0.059 | 0.942±0.052 | 0.723±0.077 | 0.596±0.676 | 0.827±0.090 | 0.765±0.245 |
| DecisionTreeClassifier with Logarithm filter | 0.191±0.530 | 0.822±0.079 | 0.938±0.094 | 0.673±0.172 | 0.957±0.021 | 0.794±0.116 | 0.729±0.169 |
| DecisionTreeClassifier with Exponential filter | 0.567±0.642 | 0.840±0.066 | 0.951±0.053 | 0.782±0.074 | 0.919±0.075 | 0.726±0.132 | 0.797±0.174 |
| DecisionTreeClassifier with LBP2D filter | 0.770±0.536 | 0.804±0.022 | 0.944±0.030 | 0.791±0.066 | 0.739±0.514 | 0.815±0.070 | 0.811±0.206 |
| DecisionTreeClassifier with Wavelet filter | 0.770±0.535 | 0.840±0.037 | 0.748±0.521 | 0.703±0.076 | 0.543±0.615 | 0.815±0.108 | 0.736±0.315 |
| RandomForestClassifier with Original filter | 0.387±0.658 | 0.736±0.043 | 0.942±0.032 | 0.765±0.073 | 0.391±0.664 | 0.802±0.082 | 0.671±0.259 |
| RandomForestClassifier with LoG filter | 0.396±0.674 | 0.701±0.031 | 0.779±0.541 | 0.787±0.066 | 0.578±0.656 | 0.825±0.057 | 0.678±0.338 |
| RandomForestClassifier with Gradient filter | 0.000±0.000 | 0.743±0.028 | 0.955±0.019 | 0.807±0.084 | 0.196±0.545 | 0.737±0.032 | 0.573±0.118 |
| RandomForestClassifier with Square filter | 0.591±0.670 | 0.721±0.055 | 0.766±0.532 | 0.752±0.064 | 0.598±0.678 | 0.804±0.093 | 0.705±0.349 |
| RandomForestClassifier with SquareRoot filter | 0.191±0.530 | 0.745±0.078 | 0.959±0.029 | 0.776±0.052 | 0.783±0.544 | 0.798±0.040 | 0.709±0.212 |
| RandomForestClassifier with Logarithm filter | 0.193±0.535 | 0.765±0.086 | 0.951±0.032 | 0.692±0.055 | 0.000±0.000 | 0.836±0.035 | 0.573±0.124 |
| RandomForestClassifier with Exponential filter | 0.394±0.671 | 0.732±0.055 | 0.959±0.048 | 0.758±0.096 | 0.394±0.671 | 0.743±0.062 | 0.664±0.267 |
| RandomForestClassifier with LBP2D filter | 0.780±0.542 | 0.760±0.026 | 0.946±0.039 | 0.771±0.049 | 0.585±0.664 | 0.775±0.057 | 0.770±0.229 |
| RandomForestClassifier with Wavelet filter | 0.391±0.664 | 0.749±0.083 | 0.968±0.021 | 0.745±0.062 | 0.983±0.025 | 0.817±0.080 | 0.776±0.156 |
| MLPClassifier with Original filter | 0.715±0.497 | 0.875±0.037 | 0.933±0.019 | 0.800±0.055 | 0.933±0.060 | 0.869±0.034 | 0.854±0.117 |
| MLPClassifier with LoG filter | 0.739±0.514 | 0.866±0.022 | 0.933±0.052 | 0.833±0.056 | 0.930±0.044 | 0.872±0.017 | 0.862±0.118 |
| MLPClassifier with Gradient filter | 0.531±0.603 | 0.844±0.062 | 0.929±0.034 | 0.789±0.038 | 0.907±0.055 | 0.861±0.040 | 0.810±0.139 |
| MLPClassifier with Square filter | 0.898±0.031 | 0.855±0.050 | 0.923±0.035 | 0.798±0.048 | 0.937±0.048 | 0.867±0.020 | 0.880±0.039 |
| MLPClassifier with SquareRoot filter | 0.524±0.595 | 0.868±0.017 | 0.912±0.024 | 0.818±0.039 | 0.919±0.062 | 0.859±0.049 | 0.817±0.131 |
| MLPClassifier with Logarithm filter | 0.737±0.513 | 0.846±0.042 | 0.916±0.018 | 0.800±0.022 | 0.920±0.030 | 0.838±0.046 | 0.843±0.112 |
| MLPClassifier with Exponential filter | 0.887±0.062 | 0.864±0.081 | 0.912±0.033 | 0.793±0.046 | 0.924±0.034 | 0.878±0.052 | 0.876±0.051 |
| MLPClassifier with LBP2D filter | 0.744±0.518 | 0.829±0.069 | 0.905±0.010 | 0.778±0.064 | 0.909±0.025 | 0.855±0.046 | 0.837±0.122 |
| MLPClassifier with Wavelet filter | 0.717±0.498 | 0.901±0.048 | 0.912±0.038 | 0.826±0.057 | 0.933±0.036 | 0.853±0.067 | 0.857±0.124 |
| AdaBoostClassifier with Original filter | 0.689±0.484 | 0.793±0.061 | 0.936±0.048 | 0.765±0.066 | 0.746±0.523 | 0.848±0.049 | 0.796±0.205 |
| AdaBoostClassifier with LoG filter | 0.719±0.500 | 0.796±0.036 | 0.966±0.028 | 0.738±0.062 | 0.939±0.054 | 0.857±0.044 | 0.836±0.121 |
| AdaBoostClassifier with Gradient filter | 0.894±0.070 | 0.822±0.097 | 0.944±0.027 | 0.734±0.094 | 0.933±0.029 | 0.842±0.038 | 0.862±0.059 |
| AdaBoostClassifier with Square filter | 0.722±0.502 | 0.815±0.098 | 0.944±0.022 | 0.760±0.044 | 0.552±0.626 | 0.834±0.089 | 0.771±0.230 |
| AdaBoostClassifier with SquareRoot filter | 0.887±0.029 | 0.813±0.093 | 0.867±0.053 | 0.756±0.103 | 0.717±0.500 | 0.895±0.105 | 0.822±0.147 |
| AdaBoostClassifier with Logarithm filter | 0.513±0.581 | 0.789±0.040 | 0.940±0.037 | 0.738±0.066 | 0.748±0.520 | 0.859±0.038 | 0.765±0.214 |
| AdaBoostClassifier with Exponential filter | 0.730±0.508 | 0.807±0.046 | 0.892±0.070 | 0.787±0.066 | 0.937±0.046 | 0.857±0.061 | 0.835±0.133 |
| AdaBoostClassifier with LBP2D filter | 0.928±0.057 | 0.798±0.045 | 0.718±0.499 | 0.811±0.059 | 0.920±0.043 | 0.792±0.064 | 0.828±0.128 |
| AdaBoostClassifier with Wavelet filter | 0.920±0.064 | 0.833±0.047 | 0.942±0.031 | 0.754±0.080 | 0.748±0.520 | 0.827±0.067 | 0.837±0.135 |
| GaussianNB with Original filter | 0.780±0.069 | 0.732±0.034 | 0.888±0.054 | 0.952±0.040 | 0.935±0.056 | 0.922±0.030 | 0.868±0.047 |
| GaussianNB with LoG filter | 0.831±0.083 | 0.758±0.067 | 0.843±0.053 | 0.985±0.021 | 0.911±0.019 | 0.943±0.012 | 0.879±0.042 |
| GaussianNB with Gradient filter | 0.683±0.052 | 0.833±0.048 | 0.793±0.036 | 0.774±0.539 | 0.572±0.649 | 0.947±0.096 | 0.767±0.236 |
| GaussianNB with Square filter | 0.759±0.090 | 0.813±0.019 | 0.832±0.045 | 0.943±0.034 | 0.187±0.519 | 0.876±0.064 | 0.735±0.129 |
| GaussianNB with SquareRoot filter | 0.722±0.075 | 0.868±0.017 | 0.852±0.063 | 0.952±0.039 | 0.911±0.062 | 0.939±0.028 | 0.874±0.047 |
| GaussianNB with Logarithm filter | 0.744±0.084 | 0.756±0.073 | 0.854±0.063 | 0.965±0.038 | 0.913±0.075 | 0.760±0.528 | 0.832±0.143 |
| GaussianNB with Exponential filter | 0.711±0.090 | 0.963±0.046 | 0.935±0.023 | 0.969±0.022 | 0.711±0.077 | 0.867±0.035 | 0.859±0.049 |
| GaussianNB with LBP2D filter | 0.783±0.082 | 0.716±0.072 | 0.867±0.088 | 0.930±0.034 | 0.748±0.520 | 0.581±0.659 | 0.771±0.242 |
| GaussianNB with Wavelet filter | 0.883±0.107 | 0.873±0.050 | 0.778±0.059 | 0.969±0.026 | 0.913±0.059 | 0.832±0.067 | 0.875±0.061 |
