# Supplementary material for: Radiomics analysis for the early diagnosis of common sexually transmitted infections and skin lesions
Source: PLOS Digit Health. 2025 Jul 23;4(7):e0000926. doi: 10.1371/journal.pdig.0000926 (PMC12286352; doi:10.1371/journal.pdig.0000926)
Supplement: S6 Table — (DOCX) [file pdig.0000926.s009.docx]

S6 Table. AUC results of the classifiers with three infection body sites.

| **Body Site** | **Model Name** | **Herpes** | **Lichen Sclerosus** | **Molluscum Contagiosum** | **Early Syphilis** | **Tinea** | **Warts** | **Total Average** |
| --- | --- | --- | --- | --- | --- | --- | --- | --- |
| Genitals | LogisticRegression with Original filter | 0.474±0.081 | 0.696±0.027 | 0.548±0.140 | 0.539±0.084 | N/A | 0.539±0.038 | 0.559±0.074 |
| Genitals | LogisticRegression with LoG filter | 0.517±0.077 | 0.789±0.024 | 0.531±0.140 | 0.630±0.139 | N/A | 0.702±0.093 | 0.634±0.095 |
| Genitals | LogisticRegression with Gradient filter | 0.509±0.098 | 0.660±0.055 | 0.646±0.171 | 0.530±0.114 | N/A | 0.577±0.076 | 0.585±0.103 |
| Genitals | LogisticRegression with Square filter | 0.517±0.042 | 0.740±0.033 | 0.594±0.176 | 0.572±0.128 | N/A | 0.643±0.050 | 0.613±0.086 |
| Genitals | LogisticRegression with SquareRoot filter | 0.495±0.075 | 0.708±0.050 | 0.492±0.010 | 0.550±0.074 | N/A | 0.550±0.075 | 0.559±0.057 |
| Genitals | LogisticRegression with Logarithm filter | 0.483±0.042 | 0.673±0.053 | 0.483±0.013 | 0.528±0.090 | N/A | 0.566±0.095 | 0.546±0.059 |
| Genitals | LogisticRegression with Exponential filter | 0.550±0.091 | 0.705±0.077 | 0.598±0.172 | 0.623±0.080 | N/A | 0.709±0.072 | 0.637±0.098 |
| Genitals | LogisticRegression with LBP2D filter | 0.499±0.050 | 0.720±0.035 | 0.644±0.175 | 0.595±0.087 | N/A | 0.634±0.133 | 0.618±0.096 |
| Genitals | LogisticRegression with Wavelet filter | 0.588±0.079 | 0.773±0.041 | 0.494±0.006 | 0.584±0.078 | N/A | 0.673±0.100 | 0.623±0.061 |
| Genitals | GBDT with Original filter | 0.511±0.080 | 0.682±0.056 | 0.596±0.169 | 0.559±0.040 | N/A | 0.736±0.108 | 0.617±0.091 |
| Genitals | GBDT with LoG filter | 0.587±0.092 | 0.780±0.081 | 0.548±0.134 | 0.608±0.066 | N/A | 0.675±0.120 | 0.639±0.098 |
| Genitals | GBDT with Gradient filter | 0.536±0.080 | 0.738±0.104 | 0.498±0.005 | 0.557±0.092 | N/A | 0.675±0.068 | 0.601±0.070 |
| Genitals | GBDT with Square filter | 0.452±0.030 | 0.730±0.059 | 0.539±0.140 | 0.606±0.090 | N/A | 0.752±0.074 | 0.616±0.079 |
| Genitals | GBDT with SquareRoot filter | 0.509±0.083 | 0.716±0.044 | 0.498±0.005 | 0.502±0.062 | N/A | 0.707±0.129 | 0.586±0.065 |
| Genitals | GBDT with Logarithm filter | 0.558±0.057 | 0.704±0.044 | 0.496±0.006 | 0.584±0.109 | N/A | 0.693±0.082 | 0.607±0.060 |
| Genitals | GBDT with Exponential filter | 0.471±0.052 | 0.720±0.040 | 0.592±0.179 | 0.601±0.066 | N/A | 0.757±0.134 | 0.628±0.094 |
| Genitals | GBDT with LBP2D filter | 0.527±0.038 | 0.704±0.024 | 0.594±0.166 | 0.559±0.031 | N/A | 0.677±0.086 | 0.612±0.069 |
| Genitals | GBDT with Wavelet filter | 0.573±0.130 | 0.752±0.018 | 0.496±0.006 | 0.637±0.091 | N/A | 0.684±0.081 | 0.628±0.065 |
| Genitals | RidgeClassifier with Original filter | 0.479±0.030 | 0.650±0.042 | 0.496±0.010 | 0.503±0.056 | N/A | 0.520±0.072 | 0.530±0.042 |
| Genitals | RidgeClassifier with LoG filter | 0.495±0.048 | 0.726±0.046 | 0.492±0.010 | 0.597±0.116 | N/A | 0.659±0.123 | 0.594±0.069 |
| Genitals | RidgeClassifier with Gradient filter | 0.501±0.054 | 0.659±0.057 | 0.594±0.171 | 0.485±0.049 | N/A | 0.577±0.087 | 0.563±0.083 |
| Genitals | RidgeClassifier with Square filter | 0.508±0.039 | 0.697±0.051 | 0.541±0.145 | 0.521±0.052 | N/A | 0.636±0.065 | 0.580±0.071 |
| Genitals | RidgeClassifier with SquareRoot filter | 0.498±0.034 | 0.650±0.031 | 0.541±0.139 | 0.538±0.075 | N/A | 0.552±0.064 | 0.556±0.069 |
| Genitals | RidgeClassifier with Logarithm filter | 0.479±0.020 | 0.646±0.030 | 0.539±0.141 | 0.525±0.056 | N/A | 0.564±0.078 | 0.551±0.065 |
| Genitals | RidgeClassifier with Exponential filter | 0.557±0.065 | 0.680±0.054 | 0.598±0.172 | 0.563±0.063 | N/A | 0.707±0.081 | 0.621±0.087 |
| Genitals | RidgeClassifier with LBP2D filter | 0.477±0.028 | 0.686±0.029 | 0.592±0.168 | 0.565±0.054 | N/A | 0.661±0.115 | 0.596±0.079 |
| Genitals | RidgeClassifier with Wavelet filter | 0.505±0.061 | 0.752±0.040 | 0.541±0.139 | 0.577±0.095 | N/A | 0.664±0.081 | 0.608±0.083 |
| Genitals | SVM with Original filter | 0.514±0.040 | 0.663±0.028 | 0.500±0.000 | 0.532±0.030 | N/A | 0.732±0.061 | 0.588±0.032 |
| Genitals | SVM with LoG filter | 0.559±0.086 | 0.766±0.054 | 0.500±0.000 | 0.659±0.138 | N/A | 0.709±0.138 | 0.639±0.083 |
| Genitals | SVM with Gradient filter | 0.489±0.001 | 0.604±0.025 | 0.500±0.000 | 0.491±0.018 | N/A | 0.636±0.060 | 0.544±0.021 |
| Genitals | SVM with Square filter | 0.500±0.000 | 0.671±0.041 | 0.500±0.000 | 0.514±0.081 | N/A | 0.693±0.036 | 0.576±0.032 |
| Genitals | SVM with SquareRoot filter | 0.524±0.042 | 0.666±0.034 | 0.500±0.000 | 0.521±0.065 | N/A | 0.766±0.076 | 0.595±0.044 |
| Genitals | SVM with Logarithm filter | 0.498±0.006 | 0.642±0.026 | 0.500±0.000 | 0.505±0.029 | N/A | 0.745±0.115 | 0.578±0.035 |
| Genitals | SVM with Exponential filter | 0.529±0.049 | 0.648±0.033 | 0.500±0.000 | 0.496±0.016 | N/A | 0.677±0.063 | 0.570±0.032 |
| Genitals | SVM with LBP2D filter | 0.514±0.056 | 0.710±0.032 | 0.500±0.000 | 0.588±0.099 | N/A | 0.707±0.105 | 0.604±0.058 |
| Genitals | SVM with Wavelet filter | 0.496±0.007 | 0.709±0.048 | 0.500±0.000 | 0.643±0.070 | N/A | 0.632±0.090 | 0.596±0.043 |
| Genitals | KNN with Original filter | 0.594±0.076 | 0.728±0.020 | 0.498±0.005 | 0.505±0.045 | N/A | 0.743±0.055 | 0.614±0.040 |
| Genitals | KNN with LoG filter | 0.608±0.084 | 0.761±0.061 | 0.500±0.000 | 0.512±0.036 | N/A | 0.723±0.096 | 0.621±0.055 |
| Genitals | KNN with Gradient filter | 0.606±0.108 | 0.700±0.033 | 0.500±0.000 | 0.555±0.036 | N/A | 0.643±0.043 | 0.601±0.044 |
| Genitals | KNN with Square filter | 0.568±0.094 | 0.772±0.056 | 0.494±0.010 | 0.530±0.080 | N/A | 0.689±0.060 | 0.610±0.060 |
| Genitals | KNN with SquareRoot filter | 0.496±0.079 | 0.670±0.038 | 0.498±0.005 | 0.529±0.063 | N/A | 0.705±0.101 | 0.580±0.057 |
| Genitals | KNN with Logarithm filter | 0.547±0.062 | 0.730±0.030 | 0.496±0.006 | 0.543±0.113 | N/A | 0.723±0.083 | 0.608±0.059 |
| Genitals | KNN with Exponential filter | 0.490±0.115 | 0.689±0.088 | 0.498±0.005 | 0.572±0.057 | N/A | 0.680±0.043 | 0.586±0.062 |
| Genitals | KNN with LBP2D filter | 0.602±0.121 | 0.735±0.063 | 0.496±0.006 | 0.539±0.078 | N/A | 0.630±0.070 | 0.600±0.068 |
| Genitals | KNN with Wavelet filter | 0.665±0.132 | 0.776±0.055 | 0.496±0.006 | 0.527±0.064 | N/A | 0.641±0.076 | 0.621±0.067 |
| Genitals | GaussianProcessClassifier with Original filter | 0.500±0.000 | 0.647±0.056 | 0.500±0.000 | 0.500±0.000 | N/A | 0.759±0.091 | 0.581±0.029 |
| Genitals | GaussianProcessClassifier with LoG filter | 0.491±0.029 | 0.750±0.054 | 0.496±0.010 | 0.545±0.122 | N/A | 0.700±0.140 | 0.596±0.071 |
| Genitals | GaussianProcessClassifier with Gradient filter | 0.483±0.034 | 0.612±0.086 | 0.500±0.000 | 0.500±0.000 | N/A | 0.625±0.115 | 0.544±0.047 |
| Genitals | GaussianProcessClassifier with Square filter | 0.500±0.000 | 0.611±0.054 | 0.498±0.005 | 0.500±0.000 | N/A | 0.718±0.055 | 0.566±0.023 |
| Genitals | GaussianProcessClassifier with SquareRoot filter | 0.496±0.012 | 0.645±0.059 | 0.498±0.005 | 0.500±0.000 | N/A | 0.748±0.134 | 0.577±0.042 |
| Genitals | GaussianProcessClassifier with Logarithm filter | 0.500±0.000 | 0.646±0.047 | 0.487±0.006 | 0.496±0.012 | N/A | 0.755±0.134 | 0.577±0.040 |
| Genitals | GaussianProcessClassifier with Exponential filter | 0.500±0.000 | 0.655±0.051 | 0.498±0.005 | 0.518±0.037 | N/A | 0.714±0.074 | 0.577±0.034 |
| Genitals | GaussianProcessClassifier with LBP2D filter | 0.483±0.015 | 0.700±0.044 | 0.648±0.168 | 0.518±0.044 | N/A | 0.677±0.107 | 0.605±0.075 |
| Genitals | GaussianProcessClassifier with Wavelet filter | 0.502±0.035 | 0.689±0.069 | 0.500±0.000 | 0.558±0.089 | N/A | 0.630±0.093 | 0.576±0.057 |
| Genitals | DecisionTreeClassifier with Original filter | 0.548±0.098 | 0.682±0.026 | 0.539±0.147 | 0.553±0.088 | N/A | 0.643±0.088 | 0.593±0.089 |
| Genitals | DecisionTreeClassifier with LoG filter | 0.643±0.083 | 0.742±0.047 | 0.491±0.012 | 0.601±0.131 | N/A | 0.652±0.104 | 0.626±0.075 |
| Genitals | DecisionTreeClassifier with Gradient filter | 0.492±0.067 | 0.656±0.036 | 0.500±0.000 | 0.502±0.138 | N/A | 0.623±0.106 | 0.555±0.069 |
| Genitals | DecisionTreeClassifier with Square filter | 0.523±0.051 | 0.761±0.070 | 0.577±0.160 | 0.497±0.050 | N/A | 0.650±0.054 | 0.602±0.077 |
| Genitals | DecisionTreeClassifier with SquareRoot filter | 0.562±0.129 | 0.703±0.100 | 0.542±0.125 | 0.501±0.070 | N/A | 0.641±0.099 | 0.590±0.104 |
| Genitals | DecisionTreeClassifier with Logarithm filter | 0.560±0.092 | 0.735±0.051 | 0.485±0.016 | 0.579±0.134 | N/A | 0.693±0.123 | 0.610±0.083 |
| Genitals | DecisionTreeClassifier with Exponential filter | 0.499±0.089 | 0.702±0.039 | 0.592±0.168 | 0.594±0.096 | N/A | 0.648±0.036 | 0.607±0.086 |
| Genitals | DecisionTreeClassifier with LBP2D filter | 0.564±0.047 | 0.703±0.066 | 0.496±0.006 | 0.588±0.051 | N/A | 0.650±0.091 | 0.600±0.052 |
| Genitals | DecisionTreeClassifier with Wavelet filter | 0.560±0.128 | 0.718±0.083 | 0.491±0.026 | 0.573±0.125 | N/A | 0.559±0.034 | 0.580±0.079 |
| Genitals | RandomForestClassifier with Original filter | 0.512±0.044 | 0.666±0.073 | 0.500±0.000 | 0.558±0.070 | N/A | 0.675±0.042 | 0.582±0.046 |
| Genitals | RandomForestClassifier with LoG filter | 0.499±0.071 | 0.701±0.014 | 0.500±0.000 | 0.532±0.056 | N/A | 0.664±0.104 | 0.579±0.049 |
| Genitals | RandomForestClassifier with Gradient filter | 0.518±0.068 | 0.717±0.049 | 0.500±0.000 | 0.550±0.033 | N/A | 0.648±0.094 | 0.586±0.049 |
| Genitals | RandomForestClassifier with Square filter | 0.528±0.081 | 0.689±0.043 | 0.548±0.140 | 0.558±0.082 | N/A | 0.707±0.072 | 0.606±0.084 |
| Genitals | RandomForestClassifier with SquareRoot filter | 0.494±0.012 | 0.675±0.058 | 0.500±0.000 | 0.518±0.074 | N/A | 0.716±0.156 | 0.581±0.060 |
| Genitals | RandomForestClassifier with Logarithm filter | 0.526±0.041 | 0.656±0.039 | 0.500±0.000 | 0.503±0.047 | N/A | 0.655±0.086 | 0.568±0.043 |
| Genitals | RandomForestClassifier with Exponential filter | 0.491±0.055 | 0.660±0.064 | 0.500±0.000 | 0.545±0.063 | N/A | 0.668±0.031 | 0.573±0.043 |
| Genitals | RandomForestClassifier with LBP2D filter | 0.559±0.075 | 0.691±0.049 | 0.548±0.140 | 0.592±0.056 | N/A | 0.702±0.105 | 0.618±0.085 |
| Genitals | RandomForestClassifier with Wavelet filter | 0.534±0.071 | 0.678±0.032 | 0.500±0.000 | 0.565±0.120 | N/A | 0.639±0.088 | 0.583±0.062 |
| Genitals | MLPClassifier with Original filter | 0.521±0.096 | 0.737±0.091 | 0.639±0.168 | 0.515±0.115 | N/A | 0.784±0.126 | 0.639±0.119 |
| Genitals | MLPClassifier with LoG filter | 0.607±0.125 | 0.820±0.086 | 0.492±0.010 | 0.586±0.126 | N/A | 0.686±0.139 | 0.638±0.097 |
| Genitals | MLPClassifier with Gradient filter | 0.631±0.087 | 0.684±0.043 | 0.648±0.168 | 0.540±0.105 | N/A | 0.641±0.084 | 0.629±0.097 |
| Genitals | MLPClassifier with Square filter | 0.464±0.049 | 0.767±0.054 | 0.596±0.169 | 0.579±0.092 | N/A | 0.734±0.101 | 0.628±0.093 |
| Genitals | MLPClassifier with SquareRoot filter | 0.494±0.119 | 0.727±0.072 | 0.537±0.148 | 0.535±0.073 | N/A | 0.784±0.091 | 0.615±0.101 |
| Genitals | MLPClassifier with Logarithm filter | 0.525±0.051 | 0.692±0.069 | 0.492±0.010 | 0.531±0.074 | N/A | 0.757±0.041 | 0.599±0.049 |
| Genitals | MLPClassifier with Exponential filter | 0.532±0.040 | 0.774±0.030 | 0.592±0.173 | 0.597±0.100 | N/A | 0.739±0.126 | 0.647±0.094 |
| Genitals | MLPClassifier with LBP2D filter | 0.556±0.098 | 0.694±0.059 | 0.592±0.168 | 0.493±0.084 | N/A | 0.611±0.129 | 0.589±0.107 |
| Genitals | MLPClassifier with Wavelet filter | 0.595±0.049 | 0.749±0.037 | 0.589±0.167 | 0.598±0.071 | N/A | 0.705±0.090 | 0.647±0.083 |
| Genitals | AdaBoostClassifier with Original filter | 0.515±0.069 | 0.678±0.065 | 0.541±0.145 | 0.515±0.047 | N/A | 0.591±0.068 | 0.568±0.079 |
| Genitals | AdaBoostClassifier with LoG filter | 0.552±0.104 | 0.655±0.069 | 0.481±0.034 | 0.609±0.066 | N/A | 0.539±0.097 | 0.567±0.074 |
| Genitals | AdaBoostClassifier with Gradient filter | 0.445±0.044 | 0.658±0.110 | 0.494±0.010 | 0.531±0.059 | N/A | 0.573±0.099 | 0.540±0.065 |
| Genitals | AdaBoostClassifier with Square filter | 0.462±0.063 | 0.626±0.057 | 0.546±0.142 | 0.542±0.114 | N/A | 0.541±0.079 | 0.544±0.091 |
| Genitals | AdaBoostClassifier with SquareRoot filter | 0.542±0.156 | 0.640±0.154 | 0.533±0.145 | 0.505±0.067 | N/A | 0.570±0.090 | 0.558±0.122 |
| Genitals | AdaBoostClassifier with Logarithm filter | 0.521±0.044 | 0.625±0.097 | 0.489±0.010 | 0.515±0.105 | N/A | 0.586±0.047 | 0.547±0.061 |
| Genitals | AdaBoostClassifier with Exponential filter | 0.478±0.102 | 0.605±0.032 | 0.494±0.010 | 0.488±0.060 | N/A | 0.539±0.091 | 0.521±0.059 |
| Genitals | AdaBoostClassifier with LBP2D filter | 0.515±0.029 | 0.704±0.089 | 0.544±0.130 | 0.519±0.078 | N/A | 0.618±0.125 | 0.580±0.090 |
| Genitals | AdaBoostClassifier with Wavelet filter | 0.507±0.096 | 0.691±0.027 | 0.494±0.010 | 0.539±0.089 | N/A | 0.650±0.093 | 0.576±0.063 |
| Genitals | GaussianNB with Original filter | 0.504±0.068 | 0.729±0.069 | 0.697±0.010 | 0.539±0.062 | N/A | 0.543±0.100 | 0.603±0.062 |
| Genitals | GaussianNB with LoG filter | 0.495±0.077 | 0.751±0.033 | 0.519±0.178 | 0.576±0.093 | N/A | 0.584±0.053 | 0.585±0.087 |
| Genitals | GaussianNB with Gradient filter | 0.499±0.055 | 0.512±0.021 | 0.499±0.169 | 0.579±0.100 | N/A | 0.505±0.029 | 0.518±0.075 |
| Genitals | GaussianNB with Square filter | 0.524±0.081 | 0.655±0.062 | 0.739±0.136 | 0.464±0.069 | N/A | 0.534±0.037 | 0.583±0.077 |
| Genitals | GaussianNB with SquareRoot filter | 0.528±0.100 | 0.760±0.034 | 0.716±0.013 | 0.521±0.041 | N/A | 0.495±0.051 | 0.604±0.048 |
| Genitals | GaussianNB with Logarithm filter | 0.465±0.056 | 0.720±0.053 | 0.474±0.015 | 0.561±0.062 | N/A | 0.518±0.064 | 0.548±0.050 |
| Genitals | GaussianNB with Exponential filter | 0.541±0.073 | 0.655±0.023 | 0.651±0.109 | 0.517±0.085 | N/A | 0.539±0.059 | 0.581±0.070 |
| Genitals | GaussianNB with LBP2D filter | 0.532±0.084 | 0.641±0.058 | 0.619±0.135 | 0.543±0.028 | N/A | 0.509±0.035 | 0.569±0.068 |
| Genitals | GaussianNB with Wavelet filter | 0.478±0.087 | 0.732±0.078 | 0.573±0.173 | 0.627±0.056 | N/A | 0.516±0.065 | 0.585±0.092 |
| Other skin | LogisticRegression with Original filter | 0.798±0.168 | 0.512±0.066 | 0.696±0.130 | 0.628±0.048 | 0.700±0.058 | 0.678±0.023 | 0.669±0.082 |
| Other skin | LogisticRegression with LoG filter | 0.715±0.072 | 0.755±0.176 | 0.727±0.111 | 0.644±0.098 | 0.729±0.033 | 0.671±0.107 | 0.707±0.099 |
| Other skin | LogisticRegression with Gradient filter | 0.635±0.148 | 0.639±0.134 | 0.704±0.071 | 0.600±0.051 | 0.675±0.068 | 0.613±0.037 | 0.644±0.085 |
| Other skin | LogisticRegression with Square filter | 0.753±0.156 | 0.489±0.005 | 0.693±0.095 | 0.624±0.058 | 0.721±0.106 | 0.677±0.107 | 0.660±0.088 |
| Other skin | LogisticRegression with SquareRoot filter | 0.836±0.070 | 0.507±0.071 | 0.676±0.081 | 0.603±0.078 | 0.723±0.094 | 0.666±0.056 | 0.669±0.075 |
| Other skin | LogisticRegression with Logarithm filter | 0.709±0.152 | 0.487±0.010 | 0.633±0.138 | 0.557±0.072 | 0.692±0.115 | 0.681±0.089 | 0.627±0.096 |
| Other skin | LogisticRegression with Exponential filter | 0.655±0.168 | 0.512±0.079 | 0.627±0.097 | 0.554±0.067 | 0.715±0.144 | 0.631±0.094 | 0.616±0.108 |
| Other skin | LogisticRegression with LBP2D filter | 0.631±0.181 | 0.520±0.067 | 0.653±0.068 | 0.681±0.098 | 0.758±0.136 | 0.605±0.020 | 0.641±0.095 |
| Other skin | LogisticRegression with Wavelet filter | 0.736±0.079 | 0.546±0.083 | 0.708±0.078 | 0.693±0.072 | 0.742±0.089 | 0.706±0.095 | 0.688±0.083 |
| Other skin | GBDT with Original filter | 0.742±0.118 | 0.534±0.083 | 0.693±0.117 | 0.628±0.066 | 0.715±0.027 | 0.681±0.106 | 0.666±0.086 |
| Other skin | GBDT with LoG filter | 0.700±0.051 | 0.629±0.208 | 0.763±0.159 | 0.689±0.056 | 0.710±0.097 | 0.741±0.096 | 0.705±0.111 |
| Other skin | GBDT with Gradient filter | 0.696±0.151 | 0.561±0.075 | 0.706±0.078 | 0.660±0.065 | 0.671±0.078 | 0.656±0.102 | 0.658±0.092 |
| Other skin | GBDT with Square filter | 0.682±0.143 | 0.537±0.074 | 0.692±0.063 | 0.600±0.057 | 0.644±0.083 | 0.654±0.085 | 0.635±0.084 |
| Other skin | GBDT with SquareRoot filter | 0.684±0.094 | 0.557±0.142 | 0.667±0.033 | 0.631±0.117 | 0.696±0.071 | 0.680±0.063 | 0.652±0.087 |
| Other skin | GBDT with Logarithm filter | 0.735±0.150 | 0.486±0.013 | 0.645±0.106 | 0.599±0.100 | 0.677±0.113 | 0.683±0.080 | 0.637±0.094 |
| Other skin | GBDT with Exponential filter | 0.711±0.136 | 0.509±0.077 | 0.678±0.064 | 0.591±0.042 | 0.690±0.055 | 0.684±0.078 | 0.644±0.075 |
| Other skin | GBDT with LBP2D filter | 0.700±0.109 | 0.516±0.063 | 0.667±0.127 | 0.660±0.068 | 0.721±0.111 | 0.586±0.118 | 0.642±0.099 |
| Other skin | GBDT with Wavelet filter | 0.705±0.140 | 0.584±0.065 | 0.700±0.121 | 0.686±0.056 | 0.735±0.086 | 0.642±0.069 | 0.675±0.090 |
| Other skin | RidgeClassifier with Original filter | 0.624±0.142 | 0.495±0.006 | 0.712±0.136 | 0.630±0.082 | 0.694±0.034 | 0.624±0.056 | 0.629±0.076 |
| Other skin | RidgeClassifier with LoG filter | 0.707±0.106 | 0.566±0.085 | 0.804±0.054 | 0.690±0.069 | 0.754±0.044 | 0.691±0.094 | 0.702±0.076 |
| Other skin | RidgeClassifier with Gradient filter | 0.596±0.107 | 0.525±0.069 | 0.638±0.081 | 0.573±0.061 | 0.696±0.100 | 0.579±0.066 | 0.601±0.081 |
| Other skin | RidgeClassifier with Square filter | 0.649±0.131 | 0.498±0.005 | 0.713±0.112 | 0.595±0.090 | 0.644±0.029 | 0.636±0.131 | 0.623±0.083 |
| Other skin | RidgeClassifier with SquareRoot filter | 0.620±0.117 | 0.486±0.015 | 0.697±0.057 | 0.636±0.074 | 0.679±0.090 | 0.641±0.047 | 0.626±0.066 |
| Other skin | RidgeClassifier with Logarithm filter | 0.655±0.204 | 0.491±0.008 | 0.666±0.091 | 0.570±0.052 | 0.660±0.092 | 0.673±0.044 | 0.619±0.082 |
| Other skin | RidgeClassifier with Exponential filter | 0.647±0.193 | 0.523±0.071 | 0.651±0.115 | 0.583±0.034 | 0.744±0.089 | 0.618±0.079 | 0.628±0.097 |
| Other skin | RidgeClassifier with LBP2D filter | 0.642±0.188 | 0.500±0.000 | 0.697±0.066 | 0.659±0.084 | 0.752±0.106 | 0.574±0.096 | 0.637±0.090 |
| Other skin | RidgeClassifier with Wavelet filter | 0.707±0.106 | 0.591±0.133 | 0.728±0.118 | 0.688±0.069 | 0.729±0.099 | 0.679±0.089 | 0.687±0.102 |
| Other skin | SVM with Original filter | 0.687±0.123 | 0.521±0.072 | 0.605±0.090 | 0.632±0.048 | 0.771±0.067 | 0.665±0.121 | 0.647±0.087 |
| Other skin | SVM with LoG filter | 0.651±0.149 | 0.580±0.076 | 0.739±0.111 | 0.739±0.083 | 0.756±0.092 | 0.704±0.097 | 0.695±0.101 |
| Other skin | SVM with Gradient filter | 0.578±0.089 | 0.500±0.000 | 0.654±0.114 | 0.665±0.046 | 0.700±0.068 | 0.592±0.055 | 0.615±0.062 |
| Other skin | SVM with Square filter | 0.671±0.183 | 0.500±0.000 | 0.608±0.123 | 0.628±0.095 | 0.735±0.091 | 0.586±0.108 | 0.621±0.100 |
| Other skin | SVM with SquareRoot filter | 0.629±0.076 | 0.520±0.073 | 0.639±0.087 | 0.597±0.081 | 0.700±0.064 | 0.672±0.127 | 0.626±0.085 |
| Other skin | SVM with Logarithm filter | 0.604±0.105 | 0.500±0.000 | 0.611±0.046 | 0.575±0.072 | 0.679±0.063 | 0.683±0.131 | 0.608±0.069 |
| Other skin | SVM with Exponential filter | 0.660±0.163 | 0.500±0.000 | 0.550±0.106 | 0.621±0.083 | 0.727±0.083 | 0.591±0.046 | 0.608±0.080 |
| Other skin | SVM with LBP2D filter | 0.633±0.166 | 0.500±0.000 | 0.704±0.147 | 0.715±0.086 | 0.744±0.124 | 0.647±0.064 | 0.657±0.098 |
| Other skin | SVM with Wavelet filter | 0.655±0.144 | 0.496±0.006 | 0.651±0.115 | 0.642±0.101 | 0.719±0.075 | 0.669±0.077 | 0.639±0.086 |
| Other skin | KNN with Original filter | 0.809±0.161 | 0.552±0.121 | 0.634±0.070 | 0.653±0.086 | 0.658±0.075 | 0.676±0.067 | 0.664±0.097 |
| Other skin | KNN with LoG filter | 0.575±0.063 | 0.654±0.120 | 0.613±0.090 | 0.623±0.074 | 0.631±0.052 | 0.640±0.083 | 0.623±0.080 |
| Other skin | KNN with Gradient filter | 0.618±0.163 | 0.523±0.083 | 0.697±0.116 | 0.594±0.065 | 0.619±0.035 | 0.538±0.077 | 0.598±0.090 |
| Other skin | KNN with Square filter | 0.765±0.074 | 0.577±0.131 | 0.649±0.121 | 0.615±0.062 | 0.679±0.060 | 0.549±0.054 | 0.639±0.084 |
| Other skin | KNN with SquareRoot filter | 0.642±0.097 | 0.584±0.114 | 0.563±0.073 | 0.622±0.068 | 0.637±0.051 | 0.655±0.093 | 0.617±0.083 |
| Other skin | KNN with Logarithm filter | 0.644±0.072 | 0.502±0.080 | 0.681±0.075 | 0.565±0.057 | 0.658±0.062 | 0.571±0.069 | 0.604±0.069 |
| Other skin | KNN with Exponential filter | 0.745±0.157 | 0.487±0.068 | 0.590±0.096 | 0.588±0.137 | 0.640±0.066 | 0.630±0.076 | 0.613±0.100 |
| Other skin | KNN with LBP2D filter | 0.605±0.199 | 0.521±0.082 | 0.609±0.131 | 0.622±0.038 | 0.665±0.113 | 0.575±0.074 | 0.600±0.106 |
| Other skin | KNN with Wavelet filter | 0.800±0.105 | 0.609±0.139 | 0.570±0.081 | 0.676±0.037 | 0.675±0.140 | 0.630±0.093 | 0.660±0.099 |
| Other skin | GaussianProcessClassifier with Original filter | 0.795±0.164 | 0.495±0.006 | 0.729±0.103 | 0.644±0.057 | 0.748±0.096 | 0.669±0.042 | 0.680±0.078 |
| Other skin | GaussianProcessClassifier with LoG filter | 0.613±0.151 | 0.568±0.078 | 0.661±0.184 | 0.612±0.140 | 0.640±0.170 | 0.627±0.155 | 0.620±0.146 |
| Other skin | GaussianProcessClassifier with Gradient filter | 0.538±0.056 | 0.500±0.000 | 0.668±0.083 | 0.620±0.062 | 0.698±0.099 | 0.615±0.063 | 0.607±0.061 |
| Other skin | GaussianProcessClassifier with Square filter | 0.720±0.063 | 0.500±0.000 | 0.689±0.137 | 0.619±0.044 | 0.746±0.096 | 0.605±0.095 | 0.646±0.072 |
| Other skin | GaussianProcessClassifier with SquareRoot filter | 0.735±0.113 | 0.495±0.006 | 0.659±0.060 | 0.588±0.072 | 0.700±0.098 | 0.678±0.098 | 0.642±0.075 |
| Other skin | GaussianProcessClassifier with Logarithm filter | 0.656±0.107 | 0.500±0.000 | 0.654±0.071 | 0.569±0.038 | 0.723±0.047 | 0.654±0.082 | 0.626±0.058 |
| Other skin | GaussianProcessClassifier with Exponential filter | 0.667±0.123 | 0.500±0.000 | 0.633±0.083 | 0.587±0.068 | 0.727±0.113 | 0.553±0.076 | 0.611±0.077 |
| Other skin | GaussianProcessClassifier with LBP2D filter | 0.685±0.173 | 0.500±0.000 | 0.719±0.124 | 0.671±0.070 | 0.750±0.077 | 0.524±0.049 | 0.642±0.082 |
| Other skin | GaussianProcessClassifier with Wavelet filter | 0.764±0.147 | 0.500±0.000 | 0.706±0.089 | 0.660±0.075 | 0.742±0.075 | 0.700±0.066 | 0.679±0.075 |
| Other skin | DecisionTreeClassifier with Original filter | 0.602±0.194 | 0.487±0.017 | 0.583±0.114 | 0.586±0.080 | 0.673±0.108 | 0.575±0.027 | 0.584±0.090 |
| Other skin | DecisionTreeClassifier with LoG filter | 0.624±0.147 | 0.561±0.139 | 0.684±0.050 | 0.622±0.097 | 0.727±0.102 | 0.620±0.124 | 0.640±0.110 |
| Other skin | DecisionTreeClassifier with Gradient filter | 0.602±0.164 | 0.512±0.081 | 0.685±0.106 | 0.611±0.091 | 0.633±0.090 | 0.571±0.089 | 0.602±0.104 |
| Other skin | DecisionTreeClassifier with Square filter | 0.509±0.096 | 0.534±0.120 | 0.647±0.153 | 0.563±0.104 | 0.675±0.104 | 0.572±0.071 | 0.583±0.108 |
| Other skin | DecisionTreeClassifier with SquareRoot filter | 0.522±0.061 | 0.566±0.075 | 0.613±0.157 | 0.545±0.064 | 0.654±0.121 | 0.596±0.079 | 0.583±0.093 |
| Other skin | DecisionTreeClassifier with Logarithm filter | 0.584±0.102 | 0.489±0.012 | 0.604±0.102 | 0.520±0.096 | 0.588±0.060 | 0.577±0.081 | 0.560±0.075 |
| Other skin | DecisionTreeClassifier with Exponential filter | 0.493±0.084 | 0.496±0.006 | 0.634±0.095 | 0.592±0.086 | 0.671±0.069 | 0.620±0.083 | 0.584±0.070 |
| Other skin | DecisionTreeClassifier with LBP2D filter | 0.569±0.165 | 0.491±0.016 | 0.675±0.118 | 0.589±0.091 | 0.665±0.071 | 0.565±0.127 | 0.592±0.098 |
| Other skin | DecisionTreeClassifier with Wavelet filter | 0.638±0.139 | 0.518±0.057 | 0.653±0.095 | 0.592±0.107 | 0.673±0.124 | 0.562±0.076 | 0.606±0.100 |
| Other skin | RandomForestClassifier with Original filter | 0.649±0.167 | 0.525±0.069 | 0.667±0.097 | 0.630±0.065 | 0.700±0.090 | 0.615±0.073 | 0.631±0.093 |
| Other skin | RandomForestClassifier with LoG filter | 0.551±0.105 | 0.520±0.073 | 0.701±0.082 | 0.662±0.057 | 0.733±0.031 | 0.660±0.126 | 0.638±0.079 |
| Other skin | RandomForestClassifier with Gradient filter | 0.593±0.125 | 0.523±0.071 | 0.641±0.063 | 0.598±0.038 | 0.679±0.058 | 0.637±0.122 | 0.612±0.080 |
| Other skin | RandomForestClassifier with Square filter | 0.544±0.072 | 0.498±0.005 | 0.586±0.073 | 0.590±0.034 | 0.694±0.094 | 0.633±0.108 | 0.591±0.064 |
| Other skin | RandomForestClassifier with SquareRoot filter | 0.635±0.070 | 0.496±0.006 | 0.607±0.023 | 0.636±0.080 | 0.700±0.054 | 0.666±0.086 | 0.624±0.053 |
| Other skin | RandomForestClassifier with Logarithm filter | 0.591±0.122 | 0.498±0.005 | 0.607±0.035 | 0.599±0.080 | 0.665±0.091 | 0.627±0.143 | 0.598±0.079 |
| Other skin | RandomForestClassifier with Exponential filter | 0.573±0.108 | 0.500±0.000 | 0.607±0.071 | 0.546±0.082 | 0.660±0.035 | 0.554±0.053 | 0.573±0.058 |
| Other skin | RandomForestClassifier with LBP2D filter | 0.655±0.168 | 0.500±0.000 | 0.644±0.088 | 0.658±0.078 | 0.756±0.088 | 0.606±0.126 | 0.636±0.092 |
| Other skin | RandomForestClassifier with Wavelet filter | 0.645±0.176 | 0.496±0.006 | 0.640±0.039 | 0.639±0.054 | 0.746±0.072 | 0.685±0.073 | 0.642±0.070 |
| Other skin | MLPClassifier with Original filter | 0.736±0.109 | 0.502±0.062 | 0.718±0.122 | 0.634±0.053 | 0.725±0.072 | 0.682±0.072 | 0.666±0.082 |
| Other skin | MLPClassifier with LoG filter | 0.676±0.101 | 0.707±0.207 | 0.681±0.078 | 0.668±0.073 | 0.742±0.060 | 0.691±0.080 | 0.694±0.100 |
| Other skin | MLPClassifier with Gradient filter | 0.720±0.212 | 0.520±0.136 | 0.706±0.079 | 0.564±0.109 | 0.652±0.103 | 0.589±0.073 | 0.625±0.119 |
| Other skin | MLPClassifier with Square filter | 0.689±0.161 | 0.479±0.013 | 0.685±0.113 | 0.618±0.046 | 0.731±0.130 | 0.694±0.143 | 0.649±0.101 |
| Other skin | MLPClassifier with SquareRoot filter | 0.804±0.077 | 0.530±0.088 | 0.683±0.054 | 0.621±0.076 | 0.706±0.094 | 0.722±0.047 | 0.678±0.072 |
| Other skin | MLPClassifier with Logarithm filter | 0.615±0.167 | 0.482±0.021 | 0.626±0.075 | 0.614±0.099 | 0.785±0.077 | 0.639±0.119 | 0.627±0.093 |
| Other skin | MLPClassifier with Exponential filter | 0.735±0.141 | 0.518±0.144 | 0.670±0.090 | 0.569±0.069 | 0.644±0.128 | 0.661±0.133 | 0.633±0.118 |
| Other skin | MLPClassifier with LBP2D filter | 0.644±0.265 | 0.479±0.019 | 0.612±0.081 | 0.620±0.093 | 0.723±0.100 | 0.564±0.083 | 0.607±0.107 |
| Other skin | MLPClassifier with Wavelet filter | 0.804±0.104 | 0.545±0.092 | 0.635±0.068 | 0.682±0.023 | 0.719±0.115 | 0.706±0.080 | 0.682±0.080 |
| Other skin | AdaBoostClassifier with Original filter | 0.585±0.083 | 0.505±0.116 | 0.600±0.130 | 0.523±0.124 | 0.615±0.107 | 0.639±0.079 | 0.578±0.107 |
| Other skin | AdaBoostClassifier with LoG filter | 0.560±0.076 | 0.552±0.091 | 0.635±0.119 | 0.563±0.086 | 0.585±0.190 | 0.540±0.131 | 0.572±0.116 |
| Other skin | AdaBoostClassifier with Gradient filter | 0.580±0.114 | 0.600±0.084 | 0.642±0.160 | 0.466±0.042 | 0.681±0.083 | 0.488±0.069 | 0.576±0.092 |
| Other skin | AdaBoostClassifier with Square filter | 0.542±0.090 | 0.504±0.079 | 0.661±0.117 | 0.582±0.094 | 0.696±0.080 | 0.571±0.056 | 0.593±0.086 |
| Other skin | AdaBoostClassifier with SquareRoot filter | 0.664±0.153 | 0.548±0.141 | 0.588±0.161 | 0.505±0.083 | 0.648±0.137 | 0.557±0.091 | 0.585±0.128 |
| Other skin | AdaBoostClassifier with Logarithm filter | 0.727±0.132 | 0.482±0.018 | 0.608±0.194 | 0.536±0.113 | 0.596±0.143 | 0.523±0.065 | 0.579±0.111 |
| Other skin | AdaBoostClassifier with Exponential filter | 0.600±0.187 | 0.525±0.080 | 0.589±0.140 | 0.521±0.096 | 0.596±0.096 | 0.560±0.082 | 0.565±0.113 |
| Other skin | AdaBoostClassifier with LBP2D filter | 0.542±0.108 | 0.539±0.107 | 0.603±0.101 | 0.557±0.047 | 0.660±0.183 | 0.527±0.043 | 0.571±0.098 |
| Other skin | AdaBoostClassifier with Wavelet filter | 0.567±0.125 | 0.504±0.045 | 0.675±0.152 | 0.594±0.081 | 0.717±0.119 | 0.619±0.030 | 0.613±0.092 |
| Other skin | GaussianNB with Original filter | 0.796±0.131 | 0.711±0.126 | 0.681±0.145 | 0.598±0.026 | 0.738±0.058 | 0.648±0.065 | 0.695±0.092 |
| Other skin | GaussianNB with LoG filter | 0.793±0.217 | 0.771±0.198 | 0.707±0.078 | 0.555±0.022 | 0.700±0.074 | 0.686±0.154 | 0.702±0.124 |
| Other skin | GaussianNB with Gradient filter | 0.756±0.160 | 0.688±0.120 | 0.771±0.141 | 0.581±0.042 | 0.690±0.091 | 0.596±0.087 | 0.680±0.107 |
| Other skin | GaussianNB with Square filter | 0.753±0.170 | 0.682±0.119 | 0.735±0.101 | 0.608±0.054 | 0.554±0.041 | 0.607±0.071 | 0.657±0.093 |
| Other skin | GaussianNB with SquareRoot filter | 0.780±0.153 | 0.768±0.088 | 0.682±0.144 | 0.617±0.042 | 0.721±0.090 | 0.646±0.031 | 0.702±0.091 |
| Other skin | GaussianNB with Logarithm filter | 0.798±0.135 | 0.632±0.124 | 0.651±0.081 | 0.592±0.058 | 0.723±0.095 | 0.577±0.041 | 0.662±0.089 |
| Other skin | GaussianNB with Exponential filter | 0.735±0.162 | 0.525±0.081 | 0.592±0.065 | 0.565±0.058 | 0.669±0.057 | 0.610±0.055 | 0.616±0.080 |
| Other skin | GaussianNB with LBP2D filter | 0.809±0.122 | 0.543±0.121 | 0.705±0.059 | 0.594±0.032 | 0.658±0.069 | 0.515±0.049 | 0.637±0.075 |
| Other skin | GaussianNB with Wavelet filter | 0.687±0.172 | 0.729±0.129 | 0.729±0.189 | 0.597±0.043 | 0.696±0.117 | 0.660±0.073 | 0.683±0.120 |
| Anus | LogisticRegression with Original filter | N/A | N/A | N/A | 0.733±0.236 | N/A | 0.733±0.236 | 0.733±0.236 |
| Anus | LogisticRegression with LoG filter | N/A | N/A | N/A | 0.567±0.376 | N/A | 0.567±0.376 | 0.567±0.376 |
| Anus | LogisticRegression with Gradient filter | N/A | N/A | N/A | 0.700±0.370 | N/A | 0.700±0.370 | 0.700±0.370 |
| Anus | LogisticRegression with Square filter | N/A | N/A | N/A | 0.600±0.236 | N/A | 0.600±0.236 | 0.600±0.236 |
| Anus | LogisticRegression with SquareRoot filter | N/A | N/A | N/A | 0.733±0.185 | N/A | 0.733±0.185 | 0.733±0.185 |
| Anus | LogisticRegression with Logarithm filter | N/A | N/A | N/A | 0.700±0.173 | N/A | 0.700±0.173 | 0.700±0.173 |
| Anus | LogisticRegression with Exponential filter | N/A | N/A | N/A | 0.500±0.358 | N/A | 0.500±0.358 | 0.500±0.358 |
| Anus | LogisticRegression with LBP2D filter | N/A | N/A | N/A | 0.533±0.307 | N/A | 0.533±0.307 | 0.533±0.307 |
| Anus | LogisticRegression with Wavelet filter | N/A | N/A | N/A | 0.700±0.307 | N/A | 0.700±0.307 | 0.700±0.307 |
| Anus | GBDT with Original filter | N/A | N/A | N/A | 0.567±0.236 | N/A | 0.567±0.236 | 0.567±0.236 |
| Anus | GBDT with LoG filter | N/A | N/A | N/A | 0.700±0.227 | N/A | 0.700±0.227 | 0.700±0.227 |
| Anus | GBDT with Gradient filter | N/A | N/A | N/A | 0.600±0.314 | N/A | 0.600±0.314 | 0.600±0.314 |
| Anus | GBDT with Square filter | N/A | N/A | N/A | 0.633±0.340 | N/A | 0.633±0.340 | 0.633±0.340 |
| Anus | GBDT with SquareRoot filter | N/A | N/A | N/A | 0.667±0.293 | N/A | 0.667±0.293 | 0.667±0.293 |
| Anus | GBDT with Logarithm filter | N/A | N/A | N/A | 0.567±0.236 | N/A | 0.567±0.236 | 0.567±0.236 |
| Anus | GBDT with Exponential filter | N/A | N/A | N/A | 0.667±0.293 | N/A | 0.667±0.293 | 0.667±0.293 |
| Anus | GBDT with LBP2D filter | N/A | N/A | N/A | 0.600±0.185 | N/A | 0.600±0.185 | 0.600±0.185 |
| Anus | GBDT with Wavelet filter | N/A | N/A | N/A | 0.667±0.207 | N/A | 0.667±0.207 | 0.667±0.207 |
| Anus | RidgeClassifier with Original filter | N/A | N/A | N/A | 0.733±0.236 | N/A | 0.733±0.236 | 0.733±0.236 |
| Anus | RidgeClassifier with LoG filter | N/A | N/A | N/A | 0.533±0.370 | N/A | 0.533±0.370 | 0.533±0.370 |
| Anus | RidgeClassifier with Gradient filter | N/A | N/A | N/A | 0.700±0.370 | N/A | 0.700±0.370 | 0.700±0.370 |
| Anus | RidgeClassifier with Square filter | N/A | N/A | N/A | 0.600±0.314 | N/A | 0.600±0.314 | 0.600±0.314 |
| Anus | RidgeClassifier with SquareRoot filter | N/A | N/A | N/A | 0.800±0.173 | N/A | 0.800±0.173 | 0.800±0.173 |
| Anus | RidgeClassifier with Logarithm filter | N/A | N/A | N/A | 0.700±0.173 | N/A | 0.700±0.173 | 0.700±0.173 |
| Anus | RidgeClassifier with Exponential filter | N/A | N/A | N/A | 0.567±0.278 | N/A | 0.567±0.278 | 0.567±0.278 |
| Anus | RidgeClassifier with LBP2D filter | N/A | N/A | N/A | 0.500±0.327 | N/A | 0.500±0.327 | 0.500±0.327 |
| Anus | RidgeClassifier with Wavelet filter | N/A | N/A | N/A | 0.700±0.307 | N/A | 0.700±0.307 | 0.700±0.307 |
| Anus | SVM with Original filter | N/A | N/A | N/A | 0.700±0.093 | N/A | 0.700±0.093 | 0.700±0.093 |
| Anus | SVM with LoG filter | N/A | N/A | N/A | 0.600±0.314 | N/A | 0.600±0.314 | 0.600±0.314 |
| Anus | SVM with Gradient filter | N/A | N/A | N/A | 0.567±0.429 | N/A | 0.567±0.429 | 0.567±0.429 |
| Anus | SVM with Square filter | N/A | N/A | N/A | 0.400±0.278 | N/A | 0.400±0.278 | 0.400±0.278 |
| Anus | SVM with SquareRoot filter | N/A | N/A | N/A | 0.700±0.173 | N/A | 0.700±0.173 | 0.700±0.173 |
| Anus | SVM with Logarithm filter | N/A | N/A | N/A | 0.700±0.173 | N/A | 0.700±0.173 | 0.700±0.173 |
| Anus | SVM with Exponential filter | N/A | N/A | N/A | 0.433±0.113 | N/A | 0.433±0.113 | 0.433±0.113 |
| Anus | SVM with LBP2D filter | N/A | N/A | N/A | 0.467±0.270 | N/A | 0.467±0.270 | 0.467±0.270 |
| Anus | SVM with Wavelet filter | N/A | N/A | N/A | 0.500±0.253 | N/A | 0.500±0.253 | 0.500±0.253 |
| Anus | KNN with Original filter | N/A | N/A | N/A | 0.700±0.093 | N/A | 0.700±0.093 | 0.700±0.093 |
| Anus | KNN with LoG filter | N/A | N/A | N/A | 0.600±0.236 | N/A | 0.600±0.236 | 0.600±0.236 |
| Anus | KNN with Gradient filter | N/A | N/A | N/A | 0.533±0.449 | N/A | 0.533±0.449 | 0.533±0.449 |
| Anus | KNN with Square filter | N/A | N/A | N/A | 0.500±0.253 | N/A | 0.500±0.253 | 0.500±0.253 |
| Anus | KNN with SquareRoot filter | N/A | N/A | N/A | 0.600±0.185 | N/A | 0.600±0.185 | 0.600±0.185 |
| Anus | KNN with Logarithm filter | N/A | N/A | N/A | 0.800±0.173 | N/A | 0.800±0.173 | 0.800±0.173 |
| Anus | KNN with Exponential filter | N/A | N/A | N/A | 0.467±0.270 | N/A | 0.467±0.270 | 0.467±0.270 |
| Anus | KNN with LBP2D filter | N/A | N/A | N/A | 0.500±0.253 | N/A | 0.500±0.253 | 0.500±0.253 |
| Anus | KNN with Wavelet filter | N/A | N/A | N/A | 0.567±0.346 | N/A | 0.567±0.346 | 0.567±0.346 |
| Anus | GaussianProcessClassifier with Original filter | N/A | N/A | N/A | 0.633±0.227 | N/A | 0.633±0.227 | 0.633±0.227 |
| Anus | GaussianProcessClassifier with LoG filter | N/A | N/A | N/A | 0.533±0.398 | N/A | 0.533±0.398 | 0.533±0.398 |
| Anus | GaussianProcessClassifier with Gradient filter | N/A | N/A | N/A | 0.600±0.453 | N/A | 0.600±0.453 | 0.600±0.453 |
| Anus | GaussianProcessClassifier with Square filter | N/A | N/A | N/A | 0.467±0.340 | N/A | 0.467±0.340 | 0.467±0.340 |
| Anus | GaussianProcessClassifier with SquareRoot filter | N/A | N/A | N/A | 0.600±0.185 | N/A | 0.600±0.185 | 0.600±0.185 |
| Anus | GaussianProcessClassifier with Logarithm filter | N/A | N/A | N/A | 0.667±0.146 | N/A | 0.667±0.146 | 0.667±0.146 |
| Anus | GaussianProcessClassifier with Exponential filter | N/A | N/A | N/A | 0.400±0.113 | N/A | 0.400±0.113 | 0.400±0.113 |
| Anus | GaussianProcessClassifier with LBP2D filter | N/A | N/A | N/A | 0.467±0.093 | N/A | 0.467±0.093 | 0.467±0.093 |
| Anus | GaussianProcessClassifier with Wavelet filter | N/A | N/A | N/A | 0.467±0.370 | N/A | 0.467±0.370 | 0.467±0.370 |
| Anus | DecisionTreeClassifier with Original filter | N/A | N/A | N/A | 0.633±0.173 | N/A | 0.633±0.173 | 0.633±0.173 |
| Anus | DecisionTreeClassifier with LoG filter | N/A | N/A | N/A | 0.533±0.340 | N/A | 0.533±0.340 | 0.533±0.340 |
| Anus | DecisionTreeClassifier with Gradient filter | N/A | N/A | N/A | 0.667±0.253 | N/A | 0.667±0.253 | 0.667±0.253 |
| Anus | DecisionTreeClassifier with Square filter | N/A | N/A | N/A | 0.533±0.173 | N/A | 0.533±0.173 | 0.533±0.173 |
| Anus | DecisionTreeClassifier with SquareRoot filter | N/A | N/A | N/A | 0.633±0.340 | N/A | 0.633±0.340 | 0.633±0.340 |
| Anus | DecisionTreeClassifier with Logarithm filter | N/A | N/A | N/A | 0.567±0.346 | N/A | 0.567±0.346 | 0.567±0.346 |
| Anus | DecisionTreeClassifier with Exponential filter | N/A | N/A | N/A | 0.733±0.278 | N/A | 0.733±0.278 | 0.733±0.278 |
| Anus | DecisionTreeClassifier with LBP2D filter | N/A | N/A | N/A | 0.667±0.293 | N/A | 0.667±0.293 | 0.667±0.293 |
| Anus | DecisionTreeClassifier with Wavelet filter | N/A | N/A | N/A | 0.733±0.113 | N/A | 0.733±0.113 | 0.733±0.113 |
| Anus | RandomForestClassifier with Original filter | N/A | N/A | N/A | 0.667±0.146 | N/A | 0.667±0.146 | 0.667±0.146 |
| Anus | RandomForestClassifier with LoG filter | N/A | N/A | N/A | 0.633±0.173 | N/A | 0.633±0.173 | 0.633±0.173 |
| Anus | RandomForestClassifier with Gradient filter | N/A | N/A | N/A | 0.500±0.293 | N/A | 0.500±0.293 | 0.500±0.293 |
| Anus | RandomForestClassifier with Square filter | N/A | N/A | N/A | 0.533±0.270 | N/A | 0.533±0.270 | 0.533±0.270 |
| Anus | RandomForestClassifier with SquareRoot filter | N/A | N/A | N/A | 0.667±0.207 | N/A | 0.667±0.207 | 0.667±0.207 |
| Anus | RandomForestClassifier with Logarithm filter | N/A | N/A | N/A | 0.667±0.253 | N/A | 0.667±0.253 | 0.667±0.253 |
| Anus | RandomForestClassifier with Exponential filter | N/A | N/A | N/A | 0.567±0.498 | N/A | 0.567±0.498 | 0.567±0.498 |
| Anus | RandomForestClassifier with LBP2D filter | N/A | N/A | N/A | 0.733±0.185 | N/A | 0.733±0.185 | 0.733±0.185 |
| Anus | RandomForestClassifier with Wavelet filter | N/A | N/A | N/A | 0.567±0.113 | N/A | 0.567±0.113 | 0.567±0.113 |
| Anus | MLPClassifier with Original filter | N/A | N/A | N/A | 0.767±0.185 | N/A | 0.767±0.185 | 0.767±0.185 |
| Anus | MLPClassifier with LoG filter | N/A | N/A | N/A | 0.600±0.314 | N/A | 0.600±0.314 | 0.600±0.314 |
| Anus | MLPClassifier with Gradient filter | N/A | N/A | N/A | 0.633±0.340 | N/A | 0.633±0.340 | 0.633±0.340 |
| Anus | MLPClassifier with Square filter | N/A | N/A | N/A | 0.533±0.173 | N/A | 0.533±0.173 | 0.533±0.173 |
| Anus | MLPClassifier with SquareRoot filter | N/A | N/A | N/A | 0.733±0.185 | N/A | 0.733±0.185 | 0.733±0.185 |
| Anus | MLPClassifier with Logarithm filter | N/A | N/A | N/A | 0.700±0.173 | N/A | 0.700±0.173 | 0.700±0.173 |
| Anus | MLPClassifier with Exponential filter | N/A | N/A | N/A | 0.467±0.270 | N/A | 0.467±0.270 | 0.467±0.270 |
| Anus | MLPClassifier with LBP2D filter | N/A | N/A | N/A | 0.467±0.227 | N/A | 0.467±0.227 | 0.467±0.227 |
| Anus | MLPClassifier with Wavelet filter | N/A | N/A | N/A | 0.567±0.314 | N/A | 0.567±0.314 | 0.567±0.314 |
| Anus | AdaBoostClassifier with Original filter | N/A | N/A | N/A | 0.533±0.270 | N/A | 0.533±0.270 | 0.533±0.270 |
| Anus | AdaBoostClassifier with LoG filter | N/A | N/A | N/A | 0.767±0.236 | N/A | 0.767±0.236 | 0.767±0.236 |
| Anus | AdaBoostClassifier with Gradient filter | N/A | N/A | N/A | 0.567±0.236 | N/A | 0.567±0.236 | 0.567±0.236 |
| Anus | AdaBoostClassifier with Square filter | N/A | N/A | N/A | 0.733±0.278 | N/A | 0.733±0.278 | 0.733±0.278 |
| Anus | AdaBoostClassifier with SquareRoot filter | N/A | N/A | N/A | 0.767±0.185 | N/A | 0.767±0.185 | 0.767±0.185 |
| Anus | AdaBoostClassifier with Logarithm filter | N/A | N/A | N/A | 0.533±0.340 | N/A | 0.533±0.340 | 0.533±0.340 |
| Anus | AdaBoostClassifier with Exponential filter | N/A | N/A | N/A | 0.633±0.173 | N/A | 0.633±0.173 | 0.633±0.173 |
| Anus | AdaBoostClassifier with LBP2D filter | N/A | N/A | N/A | 0.633±0.307 | N/A | 0.633±0.307 | 0.633±0.307 |
| Anus | AdaBoostClassifier with Wavelet filter | N/A | N/A | N/A | 0.500±0.253 | N/A | 0.500±0.253 | 0.500±0.253 |
| Anus | GaussianNB with Original filter | N/A | N/A | N/A | 0.600±0.185 | N/A | 0.600±0.185 | 0.600±0.185 |
| Anus | GaussianNB with LoG filter | N/A | N/A | N/A | 0.567±0.236 | N/A | 0.567±0.236 | 0.567±0.236 |
| Anus | GaussianNB with Gradient filter | N/A | N/A | N/A | 0.567±0.236 | N/A | 0.567±0.236 | 0.567±0.236 |
| Anus | GaussianNB with Square filter | N/A | N/A | N/A | 0.533±0.173 | N/A | 0.533±0.173 | 0.533±0.173 |
| Anus | GaussianNB with SquareRoot filter | N/A | N/A | N/A | 0.833±0.146 | N/A | 0.833±0.146 | 0.833±0.146 |
| Anus | GaussianNB with Logarithm filter | N/A | N/A | N/A | 0.600±0.113 | N/A | 0.600±0.113 | 0.600±0.113 |
| Anus | GaussianNB with Exponential filter | N/A | N/A | N/A | 0.567±0.236 | N/A | 0.567±0.236 | 0.567±0.236 |
| Anus | GaussianNB with LBP2D filter | N/A | N/A | N/A | 0.633±0.227 | N/A | 0.633±0.227 | 0.633±0.227 |
| Anus | GaussianNB with Wavelet filter | N/A | N/A | N/A | 0.500±0.253 | N/A | 0.500±0.253 | 0.500±0.253 |
