# Supplementary material for: Radiomics analysis for the early diagnosis of common sexually transmitted infections and skin lesions
Source: PLOS Digit Health. 2025 Jul 23;4(7):e0000926. doi: 10.1371/journal.pdig.0000926 (PMC12286352; doi:10.1371/journal.pdig.0000926)
Supplement: S8 Table — (DOCX) [file pdig.0000926.s011.docx]

S8 Table. Specificity results of the classifiers with three infection body sites.

| **Body Site** | **Model Name** | **Herpes** | **Lichen Sclerosus** | **Molluscum Contagiosum** | **Early Syphilis** | **Tinea** | **Warts** | **Total Average** |
| --- | --- | --- | --- | --- | --- | --- | --- | --- |
| Genitals | LogisticRegression with Original filter | 0.863±0.062 | 0.600±0.049 | 0.996±0.010 | 0.857±0.073 | N/A | 0.895±0.032 | 0.842±0.045 |
| Genitals | LogisticRegression with LoG filter | 0.892±0.076 | 0.793±0.068 | 0.962±0.029 | 0.861±0.059 | N/A | 0.895±0.051 | 0.881±0.057 |
| Genitals | LogisticRegression with Gradient filter | 0.875±0.048 | 0.559±0.088 | 0.992±0.013 | 0.861±0.053 | N/A | 0.918±0.047 | 0.841±0.050 |
| Genitals | LogisticRegression with Square filter | 0.892±0.087 | 0.634±0.094 | 0.989±0.013 | 0.900±0.073 | N/A | 0.905±0.064 | 0.864±0.066 |
| Genitals | LogisticRegression with SquareRoot filter | 0.875±0.052 | 0.593±0.093 | 0.985±0.020 | 0.878±0.109 | N/A | 0.900±0.043 | 0.846±0.063 |
| Genitals | LogisticRegression with Logarithm filter | 0.908±0.023 | 0.538±0.083 | 0.966±0.026 | 0.922±0.031 | N/A | 0.859±0.054 | 0.839±0.043 |
| Genitals | LogisticRegression with Exponential filter | 0.929±0.060 | 0.572±0.094 | 0.996±0.010 | 0.913±0.027 | N/A | 0.927±0.024 | 0.868±0.043 |
| Genitals | LogisticRegression with LBP2D filter | 0.912±0.078 | 0.655±0.030 | 0.989±0.021 | 0.878±0.024 | N/A | 0.868±0.050 | 0.861±0.041 |
| Genitals | LogisticRegression with Wavelet filter | 0.863±0.062 | 0.800±0.064 | 0.989±0.013 | 0.835±0.036 | N/A | 0.891±0.073 | 0.875±0.050 |
| Genitals | GBDT with Original filter | 0.908±0.047 | 0.572±0.099 | 0.992±0.013 | 0.896±0.080 | N/A | 0.927±0.061 | 0.859±0.060 |
| Genitals | GBDT with LoG filter | 0.887±0.087 | 0.752±0.070 | 0.996±0.010 | 0.883±0.075 | N/A | 0.877±0.068 | 0.879±0.062 |
| Genitals | GBDT with Gradient filter | 0.900±0.067 | 0.683±0.143 | 0.996±0.010 | 0.848±0.057 | N/A | 0.895±0.065 | 0.864±0.069 |
| Genitals | GBDT with Square filter | 0.904±0.060 | 0.676±0.089 | 0.977±0.010 | 0.900±0.056 | N/A | 0.886±0.072 | 0.869±0.057 |
| Genitals | GBDT with SquareRoot filter | 0.904±0.050 | 0.655±0.052 | 0.996±0.010 | 0.848±0.076 | N/A | 0.886±0.045 | 0.858±0.047 |
| Genitals | GBDT with Logarithm filter | 0.917±0.078 | 0.669±0.072 | 0.992±0.013 | 0.857±0.056 | N/A | 0.877±0.059 | 0.862±0.055 |
| Genitals | GBDT with Exponential filter | 0.912±0.043 | 0.655±0.068 | 0.985±0.020 | 0.913±0.043 | N/A | 0.877±0.043 | 0.869±0.043 |
| Genitals | GBDT with LBP2D filter | 0.883±0.023 | 0.669±0.065 | 0.989±0.021 | 0.874±0.023 | N/A | 0.882±0.037 | 0.859±0.034 |
| Genitals | GBDT with Wavelet filter | 0.917±0.048 | 0.697±0.056 | 0.992±0.013 | 0.874±0.075 | N/A | 0.895±0.043 | 0.875±0.047 |
| Genitals | RidgeClassifier with Original filter | 0.929±0.029 | 0.407±0.056 | 0.992±0.021 | 0.939±0.044 | N/A | 0.877±0.051 | 0.829±0.040 |
| Genitals | RidgeClassifier with LoG filter | 0.904±0.085 | 0.614±0.077 | 0.985±0.020 | 0.904±0.049 | N/A | 0.900±0.084 | 0.861±0.063 |
| Genitals | RidgeClassifier with Gradient filter | 0.917±0.066 | 0.448±0.142 | 0.989±0.013 | 0.948±0.056 | N/A | 0.882±0.078 | 0.837±0.071 |
| Genitals | RidgeClassifier with Square filter | 0.958±0.037 | 0.455±0.082 | 0.981±0.017 | 0.930±0.035 | N/A | 0.927±0.046 | 0.850±0.043 |
| Genitals | RidgeClassifier with SquareRoot filter | 0.967±0.039 | 0.400±0.057 | 0.981±0.017 | 0.943±0.045 | N/A | 0.886±0.080 | 0.836±0.048 |
| Genitals | RidgeClassifier with Logarithm filter | 0.958±0.041 | 0.400±0.049 | 0.977±0.031 | 0.961±0.023 | N/A | 0.873±0.074 | 0.834±0.043 |
| Genitals | RidgeClassifier with Exponential filter | 0.971±0.035 | 0.476±0.102 | 0.996±0.010 | 0.948±0.045 | N/A | 0.905±0.076 | 0.859±0.054 |
| Genitals | RidgeClassifier with LBP2D filter | 0.954±0.056 | 0.503±0.049 | 0.985±0.020 | 0.952±0.035 | N/A | 0.868±0.024 | 0.853±0.037 |
| Genitals | RidgeClassifier with Wavelet filter | 0.896±0.045 | 0.697±0.064 | 0.981±0.017 | 0.887±0.035 | N/A | 0.873±0.032 | 0.867±0.038 |
| Genitals | SVM with Original filter | 1.000±0.000 | 0.448±0.074 | 1.000±0.000 | 0.930±0.035 | N/A | 0.882±0.031 | 0.852±0.028 |
| Genitals | SVM with LoG filter | 0.975±0.056 | 0.662±0.119 | 1.000±0.000 | 0.874±0.059 | N/A | 0.891±0.070 | 0.880±0.061 |
| Genitals | SVM with Gradient filter | 0.950±0.081 | 0.324±0.127 | 1.000±0.000 | 0.983±0.035 | N/A | 0.891±0.064 | 0.830±0.062 |
| Genitals | SVM with Square filter | 1.000±0.000 | 0.428±0.089 | 1.000±0.000 | 0.939±0.035 | N/A | 0.877±0.032 | 0.849±0.031 |
| Genitals | SVM with SquareRoot filter | 0.992±0.023 | 0.455±0.088 | 1.000±0.000 | 0.952±0.048 | N/A | 0.877±0.025 | 0.855±0.037 |
| Genitals | SVM with Logarithm filter | 0.996±0.012 | 0.400±0.057 | 1.000±0.000 | 0.965±0.031 | N/A | 0.873±0.059 | 0.847±0.032 |
| Genitals | SVM with Exponential filter | 1.000±0.000 | 0.366±0.065 | 1.000±0.000 | 0.970±0.045 | N/A | 0.882±0.042 | 0.843±0.030 |
| Genitals | SVM with LBP2D filter | 0.971±0.035 | 0.566±0.083 | 1.000±0.000 | 0.909±0.044 | N/A | 0.868±0.050 | 0.863±0.043 |
| Genitals | SVM with Wavelet filter | 0.992±0.014 | 0.517±0.086 | 1.000±0.000 | 0.887±0.035 | N/A | 0.900±0.047 | 0.859±0.036 |
| Genitals | KNN with Original filter | 0.846±0.077 | 0.648±0.077 | 0.996±0.010 | 0.943±0.031 | N/A | 0.905±0.037 | 0.868±0.046 |
| Genitals | KNN with LoG filter | 0.758±0.146 | 0.738±0.120 | 1.000±0.000 | 0.935±0.038 | N/A | 0.936±0.024 | 0.873±0.065 |
| Genitals | KNN with Gradient filter | 0.812±0.052 | 0.669±0.089 | 1.000±0.000 | 0.887±0.035 | N/A | 0.923±0.032 | 0.858±0.042 |
| Genitals | KNN with Square filter | 0.821±0.050 | 0.683±0.036 | 0.989±0.021 | 0.926±0.049 | N/A | 0.941±0.038 | 0.872±0.039 |
| Genitals | KNN with SquareRoot filter | 0.792±0.071 | 0.579±0.077 | 0.996±0.010 | 0.948±0.031 | N/A | 0.936±0.024 | 0.850±0.042 |
| Genitals | KNN with Logarithm filter | 0.808±0.094 | 0.607±0.072 | 0.992±0.013 | 0.952±0.072 | N/A | 0.973±0.024 | 0.867±0.055 |
| Genitals | KNN with Exponential filter | 0.779±0.047 | 0.593±0.064 | 0.996±0.010 | 0.943±0.031 | N/A | 0.959±0.031 | 0.854±0.037 |
| Genitals | KNN with LBP2D filter | 0.775±0.043 | 0.731±0.123 | 0.992±0.013 | 0.900±0.015 | N/A | 0.914±0.031 | 0.862±0.045 |
| Genitals | KNN with Wavelet filter | 0.787±0.072 | 0.690±0.109 | 0.992±0.013 | 0.943±0.031 | N/A | 0.955±0.045 | 0.874±0.054 |
| Genitals | GaussianProcessClassifier with Original filter | 1.000±0.000 | 0.324±0.112 | 1.000±0.000 | 1.000±0.000 | N/A | 0.918±0.062 | 0.848±0.035 |
| Genitals | GaussianProcessClassifier with LoG filter | 0.954±0.059 | 0.607±0.107 | 0.992±0.021 | 0.957±0.076 | N/A | 0.818±0.113 | 0.866±0.075 |
| Genitals | GaussianProcessClassifier with Gradient filter | 0.967±0.067 | 0.517±0.341 | 0.800±0.555 | 1.000±0.000 | N/A | 0.868±0.097 | 0.830±0.212 |
| Genitals | GaussianProcessClassifier with Square filter | 1.000±0.000 | 0.269±0.111 | 0.996±0.010 | 1.000±0.000 | N/A | 0.927±0.050 | 0.838±0.034 |
| Genitals | GaussianProcessClassifier with SquareRoot filter | 0.992±0.023 | 0.352±0.093 | 0.996±0.010 | 1.000±0.000 | N/A | 0.895±0.055 | 0.847±0.036 |
| Genitals | GaussianProcessClassifier with Logarithm filter | 1.000±0.000 | 0.400±0.103 | 0.974±0.013 | 0.991±0.024 | N/A | 0.873±0.059 | 0.848±0.040 |
| Genitals | GaussianProcessClassifier with Exponential filter | 1.000±0.000 | 0.386±0.102 | 0.996±0.010 | 0.991±0.015 | N/A | 0.864±0.045 | 0.847±0.034 |
| Genitals | GaussianProcessClassifier with LBP2D filter | 0.967±0.029 | 0.531±0.089 | 0.996±0.010 | 0.970±0.041 | N/A | 0.809±0.032 | 0.855±0.040 |
| Genitals | GaussianProcessClassifier with Wavelet filter | 0.975±0.022 | 0.455±0.140 | 1.000±0.000 | 0.961±0.059 | N/A | 0.859±0.058 | 0.850±0.056 |
| Genitals | DecisionTreeClassifier with Original filter | 0.896±0.097 | 0.641±0.116 | 0.977±0.020 | 0.861±0.091 | N/A | 0.886±0.082 | 0.852±0.081 |
| Genitals | DecisionTreeClassifier with LoG filter | 0.858±0.102 | 0.745±0.123 | 0.981±0.023 | 0.870±0.087 | N/A | 0.905±0.092 | 0.872±0.086 |
| Genitals | DecisionTreeClassifier with Gradient filter | 0.842±0.087 | 0.628±0.111 | 1.000±0.000 | 0.826±0.057 | N/A | 0.900±0.038 | 0.839±0.059 |
| Genitals | DecisionTreeClassifier with Square filter | 0.846±0.149 | 0.807±0.072 | 0.955±0.049 | 0.883±0.136 | N/A | 0.827±0.133 | 0.863±0.108 |
| Genitals | DecisionTreeClassifier with SquareRoot filter | 0.867±0.014 | 0.621±0.259 | 0.985±0.031 | 0.913±0.079 | N/A | 0.882±0.078 | 0.853±0.092 |
| Genitals | DecisionTreeClassifier with Logarithm filter | 0.892±0.122 | 0.731±0.093 | 0.970±0.031 | 0.870±0.105 | N/A | 0.877±0.043 | 0.868±0.079 |
| Genitals | DecisionTreeClassifier with Exponential filter | 0.883±0.128 | 0.697±0.082 | 0.985±0.010 | 0.900±0.065 | N/A | 0.823±0.154 | 0.858±0.088 |
| Genitals | DecisionTreeClassifier with LBP2D filter | 0.871±0.072 | 0.690±0.105 | 0.992±0.013 | 0.843±0.052 | N/A | 0.900±0.065 | 0.859±0.061 |
| Genitals | DecisionTreeClassifier with Wavelet filter | 0.833±0.143 | 0.759±0.163 | 0.981±0.052 | 0.813±0.106 | N/A | 0.882±0.067 | 0.854±0.106 |
| Genitals | RandomForestClassifier with Original filter | 0.967±0.014 | 0.448±0.208 | 1.000±0.000 | 0.939±0.059 | N/A | 0.895±0.062 | 0.850±0.068 |
| Genitals | RandomForestClassifier with LoG filter | 0.942±0.050 | 0.517±0.086 | 1.000±0.000 | 0.952±0.035 | N/A | 0.855±0.074 | 0.853±0.049 |
| Genitals | RandomForestClassifier with Gradient filter | 0.921±0.028 | 0.566±0.049 | 1.000±0.000 | 0.922±0.053 | N/A | 0.877±0.062 | 0.857±0.038 |
| Genitals | RandomForestClassifier with Square filter | 0.971±0.029 | 0.462±0.099 | 0.996±0.010 | 0.939±0.035 | N/A | 0.923±0.051 | 0.858±0.045 |
| Genitals | RandomForestClassifier with SquareRoot filter | 0.988±0.023 | 0.434±0.120 | 1.000±0.000 | 0.948±0.041 | N/A | 0.886±0.069 | 0.851±0.051 |
| Genitals | RandomForestClassifier with Logarithm filter | 0.967±0.047 | 0.428±0.078 | 1.000±0.000 | 0.896±0.035 | N/A | 0.927±0.037 | 0.843±0.039 |
| Genitals | RandomForestClassifier with Exponential filter | 0.954±0.056 | 0.421±0.098 | 1.000±0.000 | 0.957±0.027 | N/A | 0.900±0.043 | 0.846±0.045 |
| Genitals | RandomForestClassifier with LBP2D filter | 0.946±0.035 | 0.552±0.113 | 0.996±0.010 | 0.939±0.040 | N/A | 0.877±0.101 | 0.862±0.060 |
| Genitals | RandomForestClassifier with Wavelet filter | 0.954±0.028 | 0.455±0.133 | 1.000±0.000 | 0.930±0.059 | N/A | 0.914±0.046 | 0.851±0.053 |
| Genitals | MLPClassifier with Original filter | 0.871±0.090 | 0.766±0.123 | 0.977±0.026 | 0.830±0.067 | N/A | 0.914±0.064 | 0.872±0.074 |
| Genitals | MLPClassifier with LoG filter | 0.871±0.087 | 0.855±0.123 | 0.985±0.020 | 0.839±0.070 | N/A | 0.882±0.037 | 0.886±0.067 |
| Genitals | MLPClassifier with Gradient filter | 0.833±0.100 | 0.745±0.120 | 0.996±0.010 | 0.813±0.041 | N/A | 0.900±0.032 | 0.857±0.061 |
| Genitals | MLPClassifier with Square filter | 0.842±0.087 | 0.779±0.049 | 0.992±0.013 | 0.870±0.085 | N/A | 0.886±0.056 | 0.874±0.058 |
| Genitals | MLPClassifier with SquareRoot filter | 0.846±0.083 | 0.738±0.127 | 0.974±0.027 | 0.870±0.043 | N/A | 0.914±0.046 | 0.868±0.065 |
| Genitals | MLPClassifier with Logarithm filter | 0.850±0.069 | 0.731±0.093 | 0.985±0.020 | 0.839±0.056 | N/A | 0.895±0.043 | 0.860±0.056 |
| Genitals | MLPClassifier with Exponential filter | 0.892±0.043 | 0.786±0.133 | 0.985±0.020 | 0.861±0.062 | N/A | 0.877±0.062 | 0.880±0.064 |
| Genitals | MLPClassifier with LBP2D filter | 0.854±0.026 | 0.703±0.072 | 0.985±0.010 | 0.809±0.052 | N/A | 0.895±0.051 | 0.849±0.042 |
| Genitals | MLPClassifier with Wavelet filter | 0.875±0.103 | 0.814±0.072 | 0.977±0.031 | 0.796±0.089 | N/A | 0.918±0.065 | 0.876±0.072 |
| Genitals | AdaBoostClassifier with Original filter | 0.829±0.127 | 0.641±0.144 | 0.981±0.017 | 0.874±0.062 | N/A | 0.891±0.092 | 0.843±0.088 |
| Genitals | AdaBoostClassifier with LoG filter | 0.904±0.087 | 0.655±0.135 | 0.962±0.068 | 0.774±0.087 | N/A | 0.914±0.037 | 0.842±0.083 |
| Genitals | AdaBoostClassifier with Gradient filter | 0.833±0.055 | 0.662±0.119 | 0.989±0.021 | 0.817±0.153 | N/A | 0.873±0.095 | 0.835±0.089 |
| Genitals | AdaBoostClassifier with Square filter | 0.896±0.071 | 0.614±0.098 | 0.992±0.013 | 0.796±0.107 | N/A | 0.845±0.097 | 0.829±0.077 |
| Genitals | AdaBoostClassifier with SquareRoot filter | 0.883±0.098 | 0.572±0.232 | 0.966±0.038 | 0.922±0.075 | N/A | 0.832±0.091 | 0.835±0.107 |
| Genitals | AdaBoostClassifier with Logarithm filter | 0.900±0.059 | 0.572±0.188 | 0.977±0.020 | 0.830±0.120 | N/A | 0.882±0.054 | 0.832±0.088 |
| Genitals | AdaBoostClassifier with Exponential filter | 0.842±0.118 | 0.572±0.173 | 0.989±0.021 | 0.843±0.124 | N/A | 0.859±0.160 | 0.821±0.119 |
| Genitals | AdaBoostClassifier with LBP2D filter | 0.917±0.063 | 0.724±0.157 | 0.989±0.021 | 0.861±0.186 | N/A | 0.764±0.109 | 0.851±0.107 |
| Genitals | AdaBoostClassifier with Wavelet filter | 0.929±0.081 | 0.690±0.086 | 0.989±0.021 | 0.857±0.109 | N/A | 0.791±0.110 | 0.851±0.081 |
| Genitals | GaussianNB with Original filter | 0.838±0.081 | 0.690±0.109 | 0.894±0.021 | 0.900±0.049 | N/A | 0.941±0.055 | 0.852±0.063 |
| Genitals | GaussianNB with LoG filter | 0.904±0.035 | 0.910±0.057 | 0.838±0.054 | 0.730±0.056 | N/A | 0.914±0.083 | 0.859±0.057 |
| Genitals | GaussianNB with Gradient filter | 0.854±0.073 | 1.000±0.000 | 0.698±0.117 | 0.513±0.102 | N/A | 0.973±0.024 | 0.808±0.063 |
| Genitals | GaussianNB with Square filter | 0.792±0.084 | 0.779±0.049 | 0.777±0.073 | 0.883±0.078 | N/A | 0.941±0.038 | 0.834±0.064 |
| Genitals | GaussianNB with SquareRoot filter | 0.771±0.095 | 0.766±0.093 | 0.932±0.027 | 0.887±0.114 | N/A | 0.918±0.032 | 0.855±0.072 |
| Genitals | GaussianNB with Logarithm filter | 0.817±0.102 | 0.655±0.109 | 0.947±0.031 | 0.878±0.130 | N/A | 0.927±0.046 | 0.845±0.084 |
| Genitals | GaussianNB with Exponential filter | 0.854±0.111 | 0.841±0.083 | 0.702±0.090 | 0.878±0.062 | N/A | 0.914±0.037 | 0.838±0.077 |
| Genitals | GaussianNB with LBP2D filter | 0.921±0.034 | 0.759±0.043 | 0.638±0.045 | 0.887±0.072 | N/A | 0.964±0.038 | 0.834±0.046 |
| Genitals | GaussianNB with Wavelet filter | 0.871±0.081 | 0.841±0.123 | 0.845±0.147 | 0.787±0.084 | N/A | 0.923±0.032 | 0.853±0.093 |
| Other skin | LogisticRegression with Original filter | 0.956±0.041 | 0.975±0.020 | 0.902±0.055 | 0.809±0.107 | 0.833±0.100 | 0.865±0.061 | 0.890±0.064 |
| Other skin | LogisticRegression with LoG filter | 0.949±0.019 | 0.961±0.029 | 0.890±0.038 | 0.842±0.043 | 0.842±0.047 | 0.906±0.073 | 0.898±0.042 |
| Other skin | LogisticRegression with Gradient filter | 0.949±0.033 | 0.979±0.019 | 0.882±0.055 | 0.800±0.086 | 0.867±0.047 | 0.808±0.096 | 0.881±0.056 |
| Other skin | LogisticRegression with Square filter | 0.945±0.036 | 0.979±0.010 | 0.878±0.025 | 0.777±0.081 | 0.875±0.084 | 0.882±0.077 | 0.889±0.052 |
| Other skin | LogisticRegression with SquareRoot filter | 0.953±0.041 | 0.964±0.027 | 0.861±0.042 | 0.758±0.095 | 0.879±0.069 | 0.914±0.011 | 0.888±0.048 |
| Other skin | LogisticRegression with Logarithm filter | 0.938±0.047 | 0.975±0.020 | 0.865±0.034 | 0.702±0.072 | 0.850±0.059 | 0.927±0.038 | 0.876±0.045 |
| Other skin | LogisticRegression with Exponential filter | 0.949±0.043 | 0.975±0.034 | 0.800±0.085 | 0.791±0.110 | 0.863±0.047 | 0.861±0.038 | 0.873±0.059 |
| Other skin | LogisticRegression with LBP2D filter | 0.942±0.033 | 0.989±0.012 | 0.869±0.029 | 0.809±0.075 | 0.850±0.046 | 0.865±0.089 | 0.888±0.047 |
| Other skin | LogisticRegression with Wavelet filter | 0.953±0.034 | 0.943±0.019 | 0.906±0.049 | 0.833±0.063 | 0.867±0.067 | 0.902±0.042 | 0.900±0.046 |
| Other skin | GBDT with Original filter | 0.964±0.023 | 0.968±0.019 | 0.878±0.031 | 0.763±0.083 | 0.879±0.043 | 0.890±0.046 | 0.890±0.041 |
| Other skin | GBDT with LoG filter | 0.960±0.019 | 0.957±0.020 | 0.927±0.042 | 0.814±0.114 | 0.887±0.050 | 0.882±0.033 | 0.904±0.046 |
| Other skin | GBDT with Gradient filter | 0.953±0.038 | 0.971±0.030 | 0.902±0.042 | 0.791±0.102 | 0.858±0.028 | 0.857±0.086 | 0.889±0.054 |
| Other skin | GBDT with Square filter | 0.964±0.032 | 0.975±0.025 | 0.894±0.028 | 0.753±0.033 | 0.838±0.043 | 0.853±0.058 | 0.879±0.036 |
| Other skin | GBDT with SquareRoot filter | 0.967±0.043 | 0.964±0.022 | 0.861±0.038 | 0.744±0.058 | 0.875±0.058 | 0.906±0.081 | 0.886±0.050 |
| Other skin | GBDT with Logarithm filter | 0.949±0.029 | 0.971±0.025 | 0.890±0.038 | 0.693±0.024 | 0.887±0.060 | 0.894±0.066 | 0.881±0.040 |
| Other skin | GBDT with Exponential filter | 0.942±0.061 | 0.968±0.043 | 0.829±0.105 | 0.781±0.026 | 0.863±0.062 | 0.914±0.066 | 0.883±0.060 |
| Other skin | GBDT with LBP2D filter | 0.960±0.025 | 0.982±0.016 | 0.861±0.042 | 0.767±0.041 | 0.892±0.038 | 0.845±0.056 | 0.885±0.036 |
| Other skin | GBDT with Wavelet filter | 0.971±0.020 | 0.968±0.024 | 0.873±0.033 | 0.819±0.105 | 0.838±0.056 | 0.902±0.033 | 0.895±0.045 |
| Other skin | RidgeClassifier with Original filter | 0.967±0.046 | 0.989±0.012 | 0.914±0.055 | 0.753±0.117 | 0.804±0.067 | 0.865±0.064 | 0.882±0.060 |
| Other skin | RidgeClassifier with LoG filter | 0.975±0.020 | 0.982±0.016 | 0.898±0.036 | 0.828±0.078 | 0.842±0.035 | 0.910±0.066 | 0.906±0.042 |
| Other skin | RidgeClassifier with Gradient filter | 0.953±0.026 | 1.000±0.000 | 0.857±0.051 | 0.735±0.090 | 0.825±0.023 | 0.849±0.087 | 0.870±0.046 |
| Other skin | RidgeClassifier with Square filter | 0.978±0.029 | 0.996±0.010 | 0.845±0.042 | 0.767±0.079 | 0.787±0.059 | 0.890±0.096 | 0.877±0.053 |
| Other skin | RidgeClassifier with SquareRoot filter | 0.960±0.033 | 0.971±0.030 | 0.886±0.029 | 0.730±0.105 | 0.825±0.075 | 0.918±0.031 | 0.882±0.050 |
| Other skin | RidgeClassifier with Logarithm filter | 0.949±0.043 | 0.982±0.016 | 0.878±0.040 | 0.693±0.047 | 0.804±0.043 | 0.947±0.038 | 0.875±0.038 |
| Other skin | RidgeClassifier with Exponential filter | 0.975±0.038 | 0.996±0.010 | 0.776±0.088 | 0.814±0.104 | 0.821±0.060 | 0.890±0.075 | 0.879±0.062 |
| Other skin | RidgeClassifier with LBP2D filter | 0.964±0.016 | 1.000±0.000 | 0.886±0.053 | 0.730±0.075 | 0.804±0.039 | 0.931±0.058 | 0.886±0.040 |
| Other skin | RidgeClassifier with Wavelet filter | 0.975±0.030 | 0.982±0.016 | 0.910±0.029 | 0.800±0.044 | 0.842±0.070 | 0.886±0.029 | 0.899±0.036 |
| Other skin | SVM with Original filter | 0.975±0.044 | 0.993±0.012 | 0.918±0.062 | 0.628±0.068 | 0.908±0.060 | 0.894±0.021 | 0.886±0.044 |
| Other skin | SVM with LoG filter | 0.982±0.016 | 0.961±0.029 | 0.914±0.021 | 0.819±0.094 | 0.863±0.039 | 0.898±0.031 | 0.906±0.038 |
| Other skin | SVM with Gradient filter | 0.956±0.030 | 1.000±0.000 | 0.890±0.066 | 0.777±0.066 | 0.833±0.084 | 0.820±0.066 | 0.879±0.052 |
| Other skin | SVM with Square filter | 0.982±0.032 | 1.000±0.000 | 0.906±0.075 | 0.609±0.063 | 0.887±0.047 | 0.882±0.094 | 0.878±0.052 |
| Other skin | SVM with SquareRoot filter | 0.978±0.029 | 0.989±0.012 | 0.914±0.055 | 0.581±0.106 | 0.883±0.054 | 0.927±0.038 | 0.879±0.049 |
| Other skin | SVM with Logarithm filter | 0.967±0.037 | 1.000±0.000 | 0.931±0.042 | 0.502±0.090 | 0.892±0.072 | 0.947±0.029 | 0.873±0.045 |
| Other skin | SVM with Exponential filter | 0.960±0.043 | 1.000±0.000 | 0.808±0.085 | 0.688±0.078 | 0.871±0.056 | 0.910±0.058 | 0.873±0.054 |
| Other skin | SVM with LBP2D filter | 0.985±0.029 | 1.000±0.000 | 0.882±0.028 | 0.772±0.085 | 0.838±0.050 | 0.894±0.092 | 0.895±0.047 |
| Other skin | SVM with Wavelet filter | 0.989±0.012 | 0.993±0.012 | 0.902±0.049 | 0.637±0.107 | 0.887±0.039 | 0.902±0.070 | 0.885±0.048 |
| Other skin | KNN with Original filter | 0.858±0.033 | 0.904±0.056 | 0.886±0.066 | 0.823±0.072 | 0.917±0.063 | 0.935±0.021 | 0.887±0.052 |
| Other skin | KNN with LoG filter | 0.829±0.047 | 0.907±0.048 | 0.845±0.066 | 0.870±0.033 | 0.863±0.039 | 0.935±0.011 | 0.875±0.041 |
| Other skin | KNN with Gradient filter | 0.876±0.037 | 0.896±0.029 | 0.849±0.053 | 0.823±0.066 | 0.871±0.050 | 0.894±0.055 | 0.868±0.048 |
| Other skin | KNN with Square filter | 0.851±0.049 | 0.954±0.012 | 0.898±0.059 | 0.772±0.047 | 0.892±0.050 | 0.898±0.054 | 0.877±0.045 |
| Other skin | KNN with SquareRoot filter | 0.844±0.038 | 0.918±0.012 | 0.890±0.061 | 0.786±0.069 | 0.892±0.062 | 0.910±0.034 | 0.873±0.046 |
| Other skin | KNN with Logarithm filter | 0.847±0.041 | 0.904±0.025 | 0.853±0.033 | 0.753±0.088 | 0.917±0.032 | 0.943±0.038 | 0.869±0.043 |
| Other skin | KNN with Exponential filter | 0.851±0.068 | 0.925±0.043 | 0.816±0.031 | 0.823±0.088 | 0.896±0.048 | 0.914±0.011 | 0.871±0.048 |
| Other skin | KNN with LBP2D filter | 0.891±0.023 | 0.893±0.035 | 0.837±0.067 | 0.809±0.056 | 0.879±0.034 | 0.914±0.038 | 0.871±0.042 |
| Other skin | KNN with Wavelet filter | 0.840±0.010 | 0.918±0.034 | 0.849±0.061 | 0.870±0.026 | 0.917±0.075 | 0.914±0.049 | 0.885±0.042 |
| Other skin | GaussianProcessClassifier with Original filter | 0.949±0.058 | 0.989±0.012 | 0.894±0.049 | 0.758±0.063 | 0.846±0.075 | 0.939±0.057 | 0.896±0.052 |
| Other skin | GaussianProcessClassifier with LoG filter | 0.785±0.546 | 0.786±0.546 | 0.922±0.095 | 0.884±0.132 | 0.912±0.101 | 0.963±0.055 | 0.876±0.246 |
| Other skin | GaussianProcessClassifier with Gradient filter | 0.956±0.041 | 1.000±0.000 | 0.882±0.049 | 0.758±0.078 | 0.812±0.045 | 0.849±0.075 | 0.876±0.048 |
| Other skin | GaussianProcessClassifier with Square filter | 0.960±0.040 | 1.000±0.000 | 0.869±0.073 | 0.721±0.084 | 0.842±0.047 | 0.918±0.062 | 0.885±0.051 |
| Other skin | GaussianProcessClassifier with SquareRoot filter | 0.949±0.029 | 0.989±0.012 | 0.882±0.033 | 0.716±0.075 | 0.817±0.059 | 0.939±0.031 | 0.882±0.040 |
| Other skin | GaussianProcessClassifier with Logarithm filter | 0.953±0.041 | 1.000±0.000 | 0.890±0.053 | 0.633±0.056 | 0.829±0.081 | 0.963±0.038 | 0.878±0.045 |
| Other skin | GaussianProcessClassifier with Exponential filter | 0.975±0.038 | 1.000±0.000 | 0.776±0.059 | 0.740±0.024 | 0.804±0.087 | 0.943±0.055 | 0.873±0.044 |
| Other skin | GaussianProcessClassifier with LBP2D filter | 0.971±0.041 | 1.000±0.000 | 0.857±0.074 | 0.730±0.090 | 0.783±0.039 | 0.976±0.033 | 0.886±0.046 |
| Other skin | GaussianProcessClassifier with Wavelet filter | 0.967±0.029 | 1.000±0.000 | 0.886±0.068 | 0.767±0.065 | 0.833±0.052 | 0.927±0.042 | 0.897±0.043 |
| Other skin | DecisionTreeClassifier with Original filter | 0.964±0.032 | 0.975±0.034 | 0.820±0.128 | 0.726±0.103 | 0.829±0.081 | 0.878±0.090 | 0.865±0.078 |
| Other skin | DecisionTreeClassifier with LoG filter | 0.927±0.058 | 0.971±0.034 | 0.914±0.049 | 0.809±0.080 | 0.838±0.038 | 0.841±0.094 | 0.883±0.059 |
| Other skin | DecisionTreeClassifier with Gradient filter | 0.964±0.016 | 0.975±0.046 | 0.861±0.108 | 0.763±0.090 | 0.833±0.048 | 0.833±0.045 | 0.871±0.059 |
| Other skin | DecisionTreeClassifier with Square filter | 0.938±0.049 | 0.968±0.048 | 0.894±0.077 | 0.726±0.230 | 0.850±0.076 | 0.816±0.130 | 0.865±0.102 |
| Other skin | DecisionTreeClassifier with SquareRoot filter | 0.964±0.042 | 0.982±0.022 | 0.935±0.058 | 0.572±0.240 | 0.858±0.115 | 0.865±0.114 | 0.863±0.098 |
| Other skin | DecisionTreeClassifier with Logarithm filter | 0.927±0.097 | 0.979±0.024 | 0.935±0.033 | 0.581±0.071 | 0.858±0.146 | 0.845±0.181 | 0.854±0.092 |
| Other skin | DecisionTreeClassifier with Exponential filter | 0.905±0.077 | 0.993±0.012 | 0.796±0.176 | 0.819±0.128 | 0.858±0.067 | 0.841±0.144 | 0.869±0.101 |
| Other skin | DecisionTreeClassifier with LBP2D filter | 0.978±0.037 | 0.982±0.031 | 0.841±0.125 | 0.753±0.172 | 0.929±0.062 | 0.731±0.089 | 0.869±0.086 |
| Other skin | DecisionTreeClassifier with Wavelet filter | 0.956±0.041 | 0.936±0.064 | 0.869±0.066 | 0.795±0.056 | 0.762±0.115 | 0.906±0.061 | 0.871±0.067 |
| Other skin | RandomForestClassifier with Original filter | 0.978±0.037 | 1.000±0.000 | 0.824±0.079 | 0.730±0.083 | 0.850±0.064 | 0.902±0.021 | 0.881±0.048 |
| Other skin | RandomForestClassifier with LoG filter | 0.982±0.016 | 0.989±0.012 | 0.894±0.045 | 0.712±0.072 | 0.850±0.012 | 0.902±0.097 | 0.888±0.042 |
| Other skin | RandomForestClassifier with Gradient filter | 0.985±0.019 | 0.996±0.010 | 0.882±0.011 | 0.702±0.088 | 0.808±0.064 | 0.873±0.033 | 0.875±0.038 |
| Other skin | RandomForestClassifier with Square filter | 0.967±0.029 | 0.996±0.010 | 0.882±0.143 | 0.651±0.071 | 0.854±0.063 | 0.865±0.075 | 0.869±0.065 |
| Other skin | RandomForestClassifier with SquareRoot filter | 0.989±0.020 | 0.993±0.012 | 0.869±0.081 | 0.614±0.026 | 0.900±0.050 | 0.914±0.045 | 0.880±0.039 |
| Other skin | RandomForestClassifier with Logarithm filter | 0.982±0.032 | 0.996±0.010 | 0.869±0.085 | 0.586±0.043 | 0.863±0.029 | 0.927±0.073 | 0.870±0.045 |
| Other skin | RandomForestClassifier with Exponential filter | 0.985±0.019 | 1.000±0.000 | 0.833±0.094 | 0.633±0.128 | 0.821±0.047 | 0.890±0.092 | 0.860±0.063 |
| Other skin | RandomForestClassifier with LBP2D filter | 0.989±0.012 | 1.000±0.000 | 0.869±0.038 | 0.740±0.133 | 0.863±0.057 | 0.849±0.102 | 0.885±0.057 |
| Other skin | RandomForestClassifier with Wavelet filter | 0.971±0.052 | 0.993±0.012 | 0.898±0.040 | 0.702±0.105 | 0.858±0.028 | 0.898±0.054 | 0.887±0.049 |
| Other skin | MLPClassifier with Original filter | 0.953±0.041 | 0.954±0.030 | 0.890±0.038 | 0.763±0.038 | 0.883±0.047 | 0.910±0.038 | 0.892±0.039 |
| Other skin | MLPClassifier with LoG filter | 0.953±0.041 | 0.964±0.016 | 0.890±0.068 | 0.819±0.056 | 0.850±0.028 | 0.910±0.046 | 0.898±0.043 |
| Other skin | MLPClassifier with Gradient filter | 0.960±0.033 | 0.939±0.030 | 0.886±0.023 | 0.740±0.085 | 0.887±0.060 | 0.833±0.033 | 0.874±0.044 |
| Other skin | MLPClassifier with Square filter | 0.938±0.054 | 0.957±0.025 | 0.861±0.038 | 0.777±0.060 | 0.896±0.071 | 0.898±0.040 | 0.888±0.048 |
| Other skin | MLPClassifier with SquareRoot filter | 0.927±0.048 | 0.961±0.019 | 0.894±0.049 | 0.795±0.043 | 0.879±0.022 | 0.898±0.031 | 0.892±0.035 |
| Other skin | MLPClassifier with Logarithm filter | 0.909±0.062 | 0.964±0.041 | 0.906±0.046 | 0.781±0.039 | 0.854±0.078 | 0.878±0.054 | 0.882±0.053 |
| Other skin | MLPClassifier with Exponential filter | 0.949±0.037 | 0.936±0.046 | 0.886±0.029 | 0.786±0.103 | 0.854±0.068 | 0.849±0.046 | 0.877±0.055 |
| Other skin | MLPClassifier with LBP2D filter | 0.927±0.045 | 0.957±0.037 | 0.878±0.018 | 0.781±0.103 | 0.863±0.070 | 0.837±0.125 | 0.874±0.066 |
| Other skin | MLPClassifier with Wavelet filter | 0.967±0.019 | 0.939±0.034 | 0.869±0.046 | 0.823±0.044 | 0.871±0.034 | 0.902±0.055 | 0.895±0.039 |
| Other skin | AdaBoostClassifier with Original filter | 0.971±0.047 | 0.911±0.065 | 0.873±0.122 | 0.740±0.277 | 0.879±0.064 | 0.788±0.181 | 0.860±0.126 |
| Other skin | AdaBoostClassifier with LoG filter | 0.880±0.109 | 0.954±0.040 | 0.869±0.075 | 0.749±0.235 | 0.837±0.059 | 0.861±0.128 | 0.858±0.108 |
| Other skin | AdaBoostClassifier with Gradient filter | 0.920±0.044 | 0.850±0.084 | 0.829±0.105 | 0.814±0.141 | 0.829±0.081 | 0.886±0.099 | 0.855±0.092 |
| Other skin | AdaBoostClassifier with Square filter | 0.964±0.050 | 0.957±0.087 | 0.849±0.125 | 0.823±0.173 | 0.792±0.140 | 0.833±0.191 | 0.870±0.128 |
| Other skin | AdaBoostClassifier with SquareRoot filter | 0.887±0.128 | 0.896±0.089 | 0.849±0.064 | 0.809±0.133 | 0.829±0.050 | 0.878±0.125 | 0.858±0.098 |
| Other skin | AdaBoostClassifier with Logarithm filter | 0.895±0.063 | 0.964±0.035 | 0.853±0.123 | 0.707±0.314 | 0.842±0.145 | 0.882±0.097 | 0.857±0.130 |
| Other skin | AdaBoostClassifier with Exponential filter | 0.920±0.044 | 0.950±0.043 | 0.796±0.065 | 0.819±0.047 | 0.775±0.085 | 0.865±0.073 | 0.854±0.059 |
| Other skin | AdaBoostClassifier with LBP2D filter | 0.924±0.065 | 0.929±0.078 | 0.861±0.112 | 0.772±0.149 | 0.871±0.088 | 0.800±0.105 | 0.859±0.100 |
| Other skin | AdaBoostClassifier with Wavelet filter | 0.975±0.034 | 0.957±0.068 | 0.841±0.105 | 0.847±0.127 | 0.783±0.118 | 0.857±0.074 | 0.877±0.088 |
| Other skin | GaussianNB with Original filter | 0.833±0.058 | 0.871±0.048 | 0.890±0.038 | 0.949±0.097 | 0.892±0.038 | 0.914±0.045 | 0.891±0.054 |
| Other skin | GaussianNB with LoG filter | 0.865±0.044 | 0.893±0.035 | 0.833±0.042 | 0.981±0.024 | 0.850±0.076 | 0.918±0.040 | 0.890±0.044 |
| Other skin | GaussianNB with Gradient filter | 0.873±0.028 | 0.925±0.065 | 0.743±0.042 | 0.986±0.026 | 0.896±0.091 | 0.902±0.083 | 0.887±0.056 |
| Other skin | GaussianNB with Square filter | 0.865±0.034 | 0.864±0.034 | 0.869±0.023 | 0.958±0.043 | 0.958±0.026 | 0.759±0.068 | 0.879±0.038 |
| Other skin | GaussianNB with SquareRoot filter | 0.800±0.050 | 0.886±0.037 | 0.837±0.047 | 0.963±0.044 | 0.925±0.039 | 0.947±0.029 | 0.893±0.041 |
| Other skin | GaussianNB with Logarithm filter | 0.796±0.051 | 0.864±0.043 | 0.865±0.105 | 0.949±0.077 | 0.846±0.050 | 0.971±0.014 | 0.882±0.057 |
| Other skin | GaussianNB with Exponential filter | 0.829±0.067 | 0.900±0.124 | 0.947±0.023 | 0.930±0.054 | 0.904±0.067 | 0.710±0.073 | 0.870±0.068 |
| Other skin | GaussianNB with LBP2D filter | 0.778±0.068 | 0.836±0.057 | 0.829±0.077 | 0.977±0.029 | 0.933±0.056 | 0.902±0.038 | 0.876±0.054 |
| Other skin | GaussianNB with Wavelet filter | 0.935±0.059 | 0.907±0.040 | 0.767±0.038 | 0.958±0.038 | 0.942±0.028 | 0.829±0.113 | 0.890±0.053 |
| Anus | LogisticRegression with Original filter | N/A | N/A | N/A | 0.733±0.185 | N/A | 0.733±0.346 | 0.733±0.266 |
| Anus | LogisticRegression with LoG filter | N/A | N/A | N/A | 0.533±0.227 | N/A | 0.600±0.540 | 0.567±0.383 |
| Anus | LogisticRegression with Gradient filter | N/A | N/A | N/A | 0.733±0.346 | N/A | 0.667±0.507 | 0.700±0.427 |
| Anus | LogisticRegression with Square filter | N/A | N/A | N/A | 0.533±0.227 | N/A | 0.667±0.414 | 0.600±0.320 |
| Anus | LogisticRegression with SquareRoot filter | N/A | N/A | N/A | 0.800±0.370 | N/A | 0.667±0.293 | 0.733±0.331 |
| Anus | LogisticRegression with Logarithm filter | N/A | N/A | N/A | 0.800±0.370 | N/A | 0.600±0.346 | 0.700±0.358 |
| Anus | LogisticRegression with Exponential filter | N/A | N/A | N/A | 0.400±0.346 | N/A | 0.600±0.540 | 0.500±0.443 |
| Anus | LogisticRegression with LBP2D filter | N/A | N/A | N/A | 0.533±0.370 | N/A | 0.533±0.370 | 0.533±0.370 |
| Anus | LogisticRegression with Wavelet filter | N/A | N/A | N/A | 0.800±0.227 | N/A | 0.600±0.453 | 0.700±0.340 |
| Anus | GBDT with Original filter | N/A | N/A | N/A | 0.667±0.293 | N/A | 0.467±0.472 | 0.567±0.382 |
| Anus | GBDT with LoG filter | N/A | N/A | N/A | 0.667±0.293 | N/A | 0.733±0.346 | 0.700±0.319 |
| Anus | GBDT with Gradient filter | N/A | N/A | N/A | 0.733±0.346 | N/A | 0.467±0.472 | 0.600±0.409 |
| Anus | GBDT with Square filter | N/A | N/A | N/A | 0.667±0.507 | N/A | 0.600±0.540 | 0.633±0.523 |
| Anus | GBDT with SquareRoot filter | N/A | N/A | N/A | 0.867±0.227 | N/A | 0.467±0.628 | 0.667±0.427 |
| Anus | GBDT with Logarithm filter | N/A | N/A | N/A | 0.733±0.346 | N/A | 0.400±0.540 | 0.567±0.443 |
| Anus | GBDT with Exponential filter | N/A | N/A | N/A | 0.733±0.346 | N/A | 0.600±0.453 | 0.667±0.400 |
| Anus | GBDT with LBP2D filter | N/A | N/A | N/A | 0.667±0.293 | N/A | 0.533±0.227 | 0.600±0.260 |
| Anus | GBDT with Wavelet filter | N/A | N/A | N/A | 0.867±0.227 | N/A | 0.467±0.370 | 0.667±0.298 |
| Anus | RidgeClassifier with Original filter | N/A | N/A | N/A | 0.800±0.227 | N/A | 0.667±0.414 | 0.733±0.320 |
| Anus | RidgeClassifier with LoG filter | N/A | N/A | N/A | 0.467±0.227 | N/A | 0.600±0.540 | 0.533±0.383 |
| Anus | RidgeClassifier with Gradient filter | N/A | N/A | N/A | 0.733±0.346 | N/A | 0.667±0.507 | 0.700±0.427 |
| Anus | RidgeClassifier with Square filter | N/A | N/A | N/A | 0.533±0.227 | N/A | 0.667±0.507 | 0.600±0.367 |
| Anus | RidgeClassifier with SquareRoot filter | N/A | N/A | N/A | 0.867±0.370 | N/A | 0.733±0.346 | 0.800±0.358 |
| Anus | RidgeClassifier with Logarithm filter | N/A | N/A | N/A | 0.800±0.370 | N/A | 0.600±0.346 | 0.700±0.358 |
| Anus | RidgeClassifier with Exponential filter | N/A | N/A | N/A | 0.533±0.370 | N/A | 0.600±0.540 | 0.567±0.455 |
| Anus | RidgeClassifier with LBP2D filter | N/A | N/A | N/A | 0.533±0.370 | N/A | 0.467±0.472 | 0.500±0.421 |
| Anus | RidgeClassifier with Wavelet filter | N/A | N/A | N/A | 0.800±0.227 | N/A | 0.600±0.453 | 0.700±0.340 |
| Anus | SVM with Original filter | N/A | N/A | N/A | 0.733±0.185 | N/A | 0.667±0.293 | 0.700±0.239 |
| Anus | SVM with LoG filter | N/A | N/A | N/A | 0.600±0.346 | N/A | 0.600±0.540 | 0.600±0.443 |
| Anus | SVM with Gradient filter | N/A | N/A | N/A | 0.533±0.370 | N/A | 0.600±0.540 | 0.567±0.455 |
| Anus | SVM with Square filter | N/A | N/A | N/A | 0.600±0.540 | N/A | 0.200±0.227 | 0.400±0.383 |
| Anus | SVM with SquareRoot filter | N/A | N/A | N/A | 0.867±0.227 | N/A | 0.533±0.472 | 0.700±0.349 |
| Anus | SVM with Logarithm filter | N/A | N/A | N/A | 0.867±0.227 | N/A | 0.533±0.227 | 0.700±0.227 |
| Anus | SVM with Exponential filter | N/A | N/A | N/A | 0.667±0.507 | N/A | 0.200±0.370 | 0.433±0.439 |
| Anus | SVM with LBP2D filter | N/A | N/A | N/A | 0.600±0.453 | N/A | 0.333±0.293 | 0.467±0.373 |
| Anus | SVM with Wavelet filter | N/A | N/A | N/A | 0.800±0.555 | N/A | 0.200±0.227 | 0.500±0.391 |
| Anus | KNN with Original filter | N/A | N/A | N/A | 0.600±0.185 | N/A | 0.800±0.227 | 0.700±0.206 |
| Anus | KNN with LoG filter | N/A | N/A | N/A | 0.533±0.227 | N/A | 0.667±0.414 | 0.600±0.320 |
| Anus | KNN with Gradient filter | N/A | N/A | N/A | 0.533±0.472 | N/A | 0.533±0.472 | 0.533±0.472 |
| Anus | KNN with Square filter | N/A | N/A | N/A | 0.533±0.227 | N/A | 0.467±0.472 | 0.500±0.349 |
| Anus | KNN with SquareRoot filter | N/A | N/A | N/A | 0.733±0.346 | N/A | 0.467±0.370 | 0.600±0.358 |
| Anus | KNN with Logarithm filter | N/A | N/A | N/A | 0.867±0.227 | N/A | 0.733±0.185 | 0.800±0.206 |
| Anus | KNN with Exponential filter | N/A | N/A | N/A | 0.533±0.227 | N/A | 0.400±0.453 | 0.467±0.340 |
| Anus | KNN with LBP2D filter | N/A | N/A | N/A | 0.533±0.370 | N/A | 0.467±0.227 | 0.500±0.298 |
| Anus | KNN with Wavelet filter | N/A | N/A | N/A | 0.467±0.370 | N/A | 0.667±0.414 | 0.567±0.392 |
| Anus | GaussianProcessClassifier with Original filter | N/A | N/A | N/A | 0.533±0.472 | N/A | 0.733±0.346 | 0.633±0.409 |
| Anus | GaussianProcessClassifier with LoG filter | N/A | N/A | N/A | 0.467±0.370 | N/A | 0.600±0.540 | 0.533±0.455 |
| Anus | GaussianProcessClassifier with Gradient filter | N/A | N/A | N/A | 0.467±0.555 | N/A | 0.733±0.540 | 0.600±0.547 |
| Anus | GaussianProcessClassifier with Square filter | N/A | N/A | N/A | 0.533±0.370 | N/A | 0.400±0.346 | 0.467±0.358 |
| Anus | GaussianProcessClassifier with SquareRoot filter | N/A | N/A | N/A | 0.467±0.555 | N/A | 0.733±0.346 | 0.600±0.451 |
| Anus | GaussianProcessClassifier with Logarithm filter | N/A | N/A | N/A | 0.667±0.507 | N/A | 0.667±0.293 | 0.667±0.400 |
| Anus | GaussianProcessClassifier with Exponential filter | N/A | N/A | N/A | 0.267±0.346 | N/A | 0.533±0.472 | 0.400±0.409 |
| Anus | GaussianProcessClassifier with LBP2D filter | N/A | N/A | N/A | 0.267±0.346 | N/A | 0.667±0.414 | 0.467±0.380 |
| Anus | GaussianProcessClassifier with Wavelet filter | N/A | N/A | N/A | 0.200±0.555 | N/A | 0.733±0.540 | 0.467±0.547 |
| Anus | DecisionTreeClassifier with Original filter | N/A | N/A | N/A | 0.733±0.346 | N/A | 0.533±0.472 | 0.633±0.409 |
| Anus | DecisionTreeClassifier with LoG filter | N/A | N/A | N/A | 0.533±0.370 | N/A | 0.533±0.555 | 0.533±0.463 |
| Anus | DecisionTreeClassifier with Gradient filter | N/A | N/A | N/A | 0.800±0.227 | N/A | 0.533±0.472 | 0.667±0.349 |
| Anus | DecisionTreeClassifier with Square filter | N/A | N/A | N/A | 0.533±0.370 | N/A | 0.533±0.472 | 0.533±0.421 |
| Anus | DecisionTreeClassifier with SquareRoot filter | N/A | N/A | N/A | 0.800±0.227 | N/A | 0.467±0.628 | 0.633±0.427 |
| Anus | DecisionTreeClassifier with Logarithm filter | N/A | N/A | N/A | 0.733±0.346 | N/A | 0.400±0.540 | 0.567±0.443 |
| Anus | DecisionTreeClassifier with Exponential filter | N/A | N/A | N/A | 0.733±0.346 | N/A | 0.733±0.346 | 0.733±0.346 |
| Anus | DecisionTreeClassifier with LBP2D filter | N/A | N/A | N/A | 0.733±0.346 | N/A | 0.600±0.346 | 0.667±0.346 |
| Anus | DecisionTreeClassifier with Wavelet filter | N/A | N/A | N/A | 0.867±0.227 | N/A | 0.600±0.185 | 0.733±0.206 |
| Anus | RandomForestClassifier with Original filter | N/A | N/A | N/A | 0.667±0.414 | N/A | 0.667±0.293 | 0.667±0.353 |
| Anus | RandomForestClassifier with LoG filter | N/A | N/A | N/A | 0.667±0.293 | N/A | 0.600±0.453 | 0.633±0.373 |
| Anus | RandomForestClassifier with Gradient filter | N/A | N/A | N/A | 0.600±0.346 | N/A | 0.400±0.453 | 0.500±0.400 |
| Anus | RandomForestClassifier with Square filter | N/A | N/A | N/A | 0.667±0.293 | N/A | 0.400±0.453 | 0.533±0.373 |
| Anus | RandomForestClassifier with SquareRoot filter | N/A | N/A | N/A | 0.667±0.293 | N/A | 0.667±0.293 | 0.667±0.293 |
| Anus | RandomForestClassifier with Logarithm filter | N/A | N/A | N/A | 0.600±0.185 | N/A | 0.733±0.346 | 0.667±0.266 |
| Anus | RandomForestClassifier with Exponential filter | N/A | N/A | N/A | 0.533±0.555 | N/A | 0.600±0.540 | 0.567±0.547 |
| Anus | RandomForestClassifier with LBP2D filter | N/A | N/A | N/A | 0.667±0.293 | N/A | 0.800±0.227 | 0.733±0.260 |
| Anus | RandomForestClassifier with Wavelet filter | N/A | N/A | N/A | 0.533±0.227 | N/A | 0.600±0.346 | 0.567±0.286 |
| Anus | MLPClassifier with Original filter | N/A | N/A | N/A | 0.733±0.185 | N/A | 0.800±0.227 | 0.767±0.206 |
| Anus | MLPClassifier with LoG filter | N/A | N/A | N/A | 0.600±0.346 | N/A | 0.600±0.540 | 0.600±0.443 |
| Anus | MLPClassifier with Gradient filter | N/A | N/A | N/A | 0.733±0.346 | N/A | 0.533±0.472 | 0.633±0.409 |
| Anus | MLPClassifier with Square filter | N/A | N/A | N/A | 0.467±0.227 | N/A | 0.600±0.346 | 0.533±0.286 |
| Anus | MLPClassifier with SquareRoot filter | N/A | N/A | N/A | 0.800±0.227 | N/A | 0.667±0.293 | 0.733±0.260 |
| Anus | MLPClassifier with Logarithm filter | N/A | N/A | N/A | 0.800±0.227 | N/A | 0.600±0.185 | 0.700±0.206 |
| Anus | MLPClassifier with Exponential filter | N/A | N/A | N/A | 0.400±0.346 | N/A | 0.533±0.472 | 0.467±0.409 |
| Anus | MLPClassifier with LBP2D filter | N/A | N/A | N/A | 0.467±0.370 | N/A | 0.467±0.227 | 0.467±0.298 |
| Anus | MLPClassifier with Wavelet filter | N/A | N/A | N/A | 0.533±0.472 | N/A | 0.600±0.346 | 0.567±0.409 |
| Anus | AdaBoostClassifier with Original filter | N/A | N/A | N/A | 0.533±0.227 | N/A | 0.533±0.472 | 0.533±0.349 |
| Anus | AdaBoostClassifier with LoG filter | N/A | N/A | N/A | 0.867±0.227 | N/A | 0.667±0.507 | 0.767±0.367 |
| Anus | AdaBoostClassifier with Gradient filter | N/A | N/A | N/A | 0.533±0.227 | N/A | 0.600±0.346 | 0.567±0.286 |
| Anus | AdaBoostClassifier with Square filter | N/A | N/A | N/A | 0.733±0.540 | N/A | 0.733±0.346 | 0.733±0.443 |
| Anus | AdaBoostClassifier with SquareRoot filter | N/A | N/A | N/A | 0.867±0.227 | N/A | 0.667±0.507 | 0.767±0.367 |
| Anus | AdaBoostClassifier with Logarithm filter | N/A | N/A | N/A | 0.667±0.414 | N/A | 0.400±0.540 | 0.533±0.477 |
| Anus | AdaBoostClassifier with Exponential filter | N/A | N/A | N/A | 0.733±0.346 | N/A | 0.533±0.472 | 0.633±0.409 |
| Anus | AdaBoostClassifier with LBP2D filter | N/A | N/A | N/A | 0.800±0.227 | N/A | 0.467±0.472 | 0.633±0.349 |
| Anus | AdaBoostClassifier with Wavelet filter | N/A | N/A | N/A | 0.600±0.346 | N/A | 0.400±0.453 | 0.500±0.400 |
| Anus | GaussianNB with Original filter | N/A | N/A | N/A | 0.533±0.227 | N/A | 0.667±0.293 | 0.600±0.260 |
| Anus | GaussianNB with LoG filter | N/A | N/A | N/A | 0.667±0.293 | N/A | 0.467±0.472 | 0.567±0.382 |
| Anus | GaussianNB with Gradient filter | N/A | N/A | N/A | 0.533±0.227 | N/A | 0.600±0.346 | 0.567±0.286 |
| Anus | GaussianNB with Square filter | N/A | N/A | N/A | 0.467±0.227 | N/A | 0.600±0.346 | 0.533±0.286 |
| Anus | GaussianNB with SquareRoot filter | N/A | N/A | N/A | 0.933±0.185 | N/A | 0.733±0.346 | 0.833±0.266 |
| Anus | GaussianNB with Logarithm filter | N/A | N/A | N/A | 0.800±0.227 | N/A | 0.400±0.185 | 0.600±0.206 |
| Anus | GaussianNB with Exponential filter | N/A | N/A | N/A | 0.533±0.370 | N/A | 0.600±0.346 | 0.567±0.358 |
| Anus | GaussianNB with LBP2D filter | N/A | N/A | N/A | 0.533±0.227 | N/A | 0.733±0.346 | 0.633±0.286 |
| Anus | GaussianNB with Wavelet filter | N/A | N/A | N/A | 0.667±0.414 | N/A | 0.333±0.414 | 0.500±0.414 |
