# Supplementary material for: Radiomics analysis for the early diagnosis of common sexually transmitted infections and skin lesions
Source: PLOS Digit Health. 2025 Jul 23;4(7):e0000926. doi: 10.1371/journal.pdig.0000926 (PMC12286352; doi:10.1371/journal.pdig.0000926)
Supplement: S9 Table — (DOCX) [file pdig.0000926.s012.docx]

S9 Table. Sensitivity results of the classifiers with three infection body sites.

| **Body Site** | **Model Name** | **Herpes** | **Lichen Sclerosus** | **Molluscum Contagiosum** | **Early Syphilis** | **Tinea** | **Warts** | **Total Average** |
| --- | --- | --- | --- | --- | --- | --- | --- | --- |
| Genitals | LogisticRegression with Original filter | 0.086±0.159 | 0.792±0.043 | 0.100±0.278 | 0.222±0.169 | N/A | 0.182±0.080 | 0.276±0.146 |
| Genitals | LogisticRegression with LoG filter | 0.143±0.125 | 0.785±0.043 | 0.100±0.278 | 0.400±0.231 | N/A | 0.509±0.189 | 0.387±0.173 |
| Genitals | LogisticRegression with Gradient filter | 0.143±0.217 | 0.762±0.085 | 0.300±0.340 | 0.200±0.227 | N/A | 0.236±0.129 | 0.328±0.200 |
| Genitals | LogisticRegression with Square filter | 0.143±0.125 | 0.846±0.083 | 0.200±0.340 | 0.244±0.227 | N/A | 0.382±0.147 | 0.363±0.184 |
| Genitals | LogisticRegression with SquareRoot filter | 0.114±0.148 | 0.823±0.054 | 0.000±0.000 | 0.222±0.195 | N/A | 0.200±0.147 | 0.272±0.109 |
| Genitals | LogisticRegression with Logarithm filter | 0.057±0.097 | 0.808±0.058 | 0.000±0.000 | 0.133±0.180 | N/A | 0.273±0.178 | 0.254±0.103 |
| Genitals | LogisticRegression with Exponential filter | 0.171±0.148 | 0.838±0.114 | 0.200±0.340 | 0.333±0.169 | N/A | 0.491±0.129 | 0.407±0.180 |
| Genitals | LogisticRegression with LBP2D filter | 0.086±0.097 | 0.785±0.054 | 0.300±0.340 | 0.311±0.180 | N/A | 0.400±0.220 | 0.376±0.178 |
| Genitals | LogisticRegression with Wavelet filter | 0.314±0.194 | 0.746±0.072 | 0.000±0.000 | 0.333±0.169 | N/A | 0.455±0.160 | 0.370±0.119 |
| Genitals | GBDT with Original filter | 0.114±0.148 | 0.792±0.105 | 0.200±0.340 | 0.222±0.000 | N/A | 0.545±0.178 | 0.375±0.154 |
| Genitals | GBDT with LoG filter | 0.286±0.217 | 0.808±0.139 | 0.100±0.278 | 0.333±0.138 | N/A | 0.473±0.217 | 0.400±0.198 |
| Genitals | GBDT with Gradient filter | 0.171±0.194 | 0.792±0.129 | 0.000±0.000 | 0.267±0.157 | N/A | 0.455±0.138 | 0.337±0.124 |
| Genitals | GBDT with Square filter | 0.000±0.000 | 0.785±0.080 | 0.100±0.278 | 0.311±0.180 | N/A | 0.618±0.124 | 0.363±0.132 |
| Genitals | GBDT with SquareRoot filter | 0.114±0.194 | 0.777±0.071 | 0.000±0.000 | 0.156±0.076 | N/A | 0.527±0.257 | 0.315±0.120 |
| Genitals | GBDT with Logarithm filter | 0.200±0.097 | 0.738±0.078 | 0.000±0.000 | 0.311±0.205 | N/A | 0.509±0.129 | 0.352±0.102 |
| Genitals | GBDT with Exponential filter | 0.029±0.079 | 0.785±0.043 | 0.200±0.340 | 0.289±0.157 | N/A | 0.636±0.252 | 0.388±0.174 |
| Genitals | GBDT with LBP2D filter | 0.171±0.079 | 0.738±0.040 | 0.200±0.340 | 0.244±0.062 | N/A | 0.473±0.147 | 0.365±0.134 |
| Genitals | GBDT with Wavelet filter | 0.229±0.269 | 0.808±0.048 | 0.000±0.000 | 0.400±0.185 | N/A | 0.473±0.147 | 0.382±0.130 |
| Genitals | RidgeClassifier with Original filter | 0.029±0.079 | 0.892±0.040 | 0.000±0.000 | 0.067±0.123 | N/A | 0.164±0.094 | 0.230±0.067 |
| Genitals | RidgeClassifier with LoG filter | 0.086±0.097 | 0.838±0.062 | 0.000±0.000 | 0.289±0.209 | N/A | 0.418±0.234 | 0.326±0.121 |
| Genitals | RidgeClassifier with Gradient filter | 0.086±0.097 | 0.869±0.054 | 0.200±0.340 | 0.022±0.062 | N/A | 0.273±0.113 | 0.290±0.133 |
| Genitals | RidgeClassifier with Square filter | 0.057±0.097 | 0.938±0.054 | 0.100±0.278 | 0.111±0.098 | N/A | 0.345±0.124 | 0.310±0.130 |
| Genitals | RidgeClassifier with SquareRoot filter | 0.029±0.079 | 0.900±0.043 | 0.100±0.278 | 0.133±0.180 | N/A | 0.218±0.062 | 0.276±0.128 |
| Genitals | RidgeClassifier with Logarithm filter | 0.000±0.000 | 0.892±0.062 | 0.100±0.278 | 0.089±0.115 | N/A | 0.255±0.124 | 0.267±0.116 |
| Genitals | RidgeClassifier with Exponential filter | 0.143±0.125 | 0.885±0.058 | 0.200±0.340 | 0.178±0.123 | N/A | 0.509±0.101 | 0.383±0.150 |
| Genitals | RidgeClassifier with LBP2D filter | 0.000±0.000 | 0.869±0.026 | 0.200±0.340 | 0.178±0.076 | N/A | 0.455±0.211 | 0.340±0.131 |
| Genitals | RidgeClassifier with Wavelet filter | 0.114±0.148 | 0.808±0.068 | 0.100±0.278 | 0.267±0.185 | N/A | 0.455±0.138 | 0.349±0.163 |
| Genitals | SVM with Original filter | 0.029±0.079 | 0.877±0.062 | 0.000±0.000 | 0.133±0.062 | N/A | 0.582±0.129 | 0.324±0.066 |
| Genitals | SVM with LoG filter | 0.143±0.217 | 0.869±0.072 | 0.000±0.000 | 0.444±0.258 | N/A | 0.527±0.231 | 0.397±0.156 |
| Genitals | SVM with Gradient filter | 0.029±0.079 | 0.885±0.131 | 0.000±0.000 | 0.000±0.000 | N/A | 0.382±0.124 | 0.259±0.067 |
| Genitals | SVM with Square filter | 0.000±0.000 | 0.915±0.021 | 0.000±0.000 | 0.089±0.151 | N/A | 0.509±0.062 | 0.303±0.047 |
| Genitals | SVM with SquareRoot filter | 0.057±0.097 | 0.877±0.062 | 0.000±0.000 | 0.089±0.115 | N/A | 0.655±0.147 | 0.336±0.084 |
| Genitals | SVM with Logarithm filter | 0.000±0.000 | 0.885±0.068 | 0.000±0.000 | 0.044±0.076 | N/A | 0.618±0.185 | 0.309±0.066 |
| Genitals | SVM with Exponential filter | 0.057±0.097 | 0.931±0.062 | 0.000±0.000 | 0.022±0.062 | N/A | 0.473±0.094 | 0.297±0.063 |
| Genitals | SVM with LBP2D filter | 0.057±0.097 | 0.854±0.040 | 0.000±0.000 | 0.267±0.209 | N/A | 0.545±0.160 | 0.345±0.101 |
| Genitals | SVM with Wavelet filter | 0.000±0.000 | 0.900±0.054 | 0.000±0.000 | 0.400±0.157 | N/A | 0.364±0.138 | 0.333±0.070 |
| Genitals | KNN with Original filter | 0.343±0.202 | 0.808±0.089 | 0.000±0.000 | 0.067±0.076 | N/A | 0.582±0.101 | 0.360±0.094 |
| Genitals | KNN with LoG filter | 0.457±0.231 | 0.785±0.087 | 0.000±0.000 | 0.089±0.062 | N/A | 0.509±0.189 | 0.368±0.114 |
| Genitals | KNN with Gradient filter | 0.400±0.231 | 0.731±0.126 | 0.000±0.000 | 0.222±0.098 | N/A | 0.364±0.080 | 0.343±0.107 |
| Genitals | KNN with Square filter | 0.314±0.148 | 0.862±0.110 | 0.000±0.000 | 0.133±0.115 | N/A | 0.436±0.094 | 0.349±0.094 |
| Genitals | KNN with SquareRoot filter | 0.200±0.202 | 0.762±0.085 | 0.000±0.000 | 0.111±0.098 | N/A | 0.473±0.202 | 0.309±0.117 |
| Genitals | KNN with Logarithm filter | 0.286±0.125 | 0.854±0.085 | 0.000±0.000 | 0.133±0.180 | N/A | 0.473±0.147 | 0.349±0.108 |
| Genitals | KNN with Exponential filter | 0.200±0.202 | 0.785±0.138 | 0.000±0.000 | 0.200±0.115 | N/A | 0.400±0.062 | 0.317±0.103 |
| Genitals | KNN with LBP2D filter | 0.429±0.217 | 0.738±0.062 | 0.000±0.000 | 0.178±0.157 | N/A | 0.345±0.124 | 0.338±0.112 |
| Genitals | KNN with Wavelet filter | 0.543±0.231 | 0.862±0.072 | 0.000±0.000 | 0.111±0.138 | N/A | 0.327±0.129 | 0.369±0.114 |
| Genitals | GaussianProcessClassifier with Original filter | 0.000±0.000 | 0.969±0.021 | 0.000±0.000 | 0.000±0.000 | N/A | 0.600±0.171 | 0.314±0.039 |
| Genitals | GaussianProcessClassifier with LoG filter | 0.029±0.079 | 0.892±0.071 | 0.000±0.000 | 0.133±0.247 | N/A | 0.582±0.171 | 0.327±0.114 |
| Genitals | GaussianProcessClassifier with Gradient filter | 0.000±0.000 | 0.708±0.495 | 0.200±0.555 | 0.000±0.000 | N/A | 0.382±0.303 | 0.258±0.271 |
| Genitals | GaussianProcessClassifier with Square filter | 0.000±0.000 | 0.954±0.021 | 0.000±0.000 | 0.000±0.000 | N/A | 0.509±0.129 | 0.293±0.030 |
| Genitals | GaussianProcessClassifier with SquareRoot filter | 0.000±0.000 | 0.938±0.043 | 0.000±0.000 | 0.000±0.000 | N/A | 0.600±0.234 | 0.308±0.055 |
| Genitals | GaussianProcessClassifier with Logarithm filter | 0.000±0.000 | 0.892±0.062 | 0.000±0.000 | 0.000±0.000 | N/A | 0.636±0.252 | 0.306±0.063 |
| Genitals | GaussianProcessClassifier with Exponential filter | 0.000±0.000 | 0.923±0.000 | 0.000±0.000 | 0.044±0.076 | N/A | 0.564±0.147 | 0.306±0.045 |
| Genitals | GaussianProcessClassifier with LBP2D filter | 0.000±0.000 | 0.869±0.054 | 0.300±0.340 | 0.067±0.076 | N/A | 0.545±0.226 | 0.356±0.139 |
| Genitals | GaussianProcessClassifier with Wavelet filter | 0.029±0.079 | 0.923±0.068 | 0.000±0.000 | 0.156±0.231 | N/A | 0.400±0.151 | 0.301±0.106 |
| Genitals | DecisionTreeClassifier with Original filter | 0.200±0.269 | 0.723±0.124 | 0.100±0.278 | 0.244±0.247 | N/A | 0.400±0.151 | 0.334±0.214 |
| Genitals | DecisionTreeClassifier with LoG filter | 0.429±0.217 | 0.738±0.098 | 0.000±0.000 | 0.333±0.218 | N/A | 0.400±0.205 | 0.380±0.148 |
| Genitals | DecisionTreeClassifier with Gradient filter | 0.143±0.217 | 0.685±0.156 | 0.000±0.000 | 0.178±0.318 | N/A | 0.345±0.217 | 0.270±0.182 |
| Genitals | DecisionTreeClassifier with Square filter | 0.200±0.159 | 0.715±0.157 | 0.200±0.340 | 0.111±0.195 | N/A | 0.473±0.185 | 0.340±0.207 |
| Genitals | DecisionTreeClassifier with SquareRoot filter | 0.257±0.263 | 0.785±0.110 | 0.100±0.278 | 0.089±0.115 | N/A | 0.400±0.189 | 0.326±0.191 |
| Genitals | DecisionTreeClassifier with Logarithm filter | 0.229±0.202 | 0.738±0.078 | 0.000±0.000 | 0.289±0.251 | N/A | 0.509±0.205 | 0.353±0.147 |
| Genitals | DecisionTreeClassifier with Exponential filter | 0.114±0.194 | 0.708±0.043 | 0.200±0.340 | 0.289±0.251 | N/A | 0.473±0.202 | 0.357±0.206 |
| Genitals | DecisionTreeClassifier with LBP2D filter | 0.257±0.079 | 0.715±0.080 | 0.000±0.000 | 0.333±0.098 | N/A | 0.400±0.189 | 0.341±0.089 |
| Genitals | DecisionTreeClassifier with Wavelet filter | 0.286±0.376 | 0.677±0.064 | 0.000±0.000 | 0.333±0.195 | N/A | 0.236±0.062 | 0.306±0.139 |
| Genitals | RandomForestClassifier with Original filter | 0.057±0.097 | 0.885±0.089 | 0.000±0.000 | 0.178±0.185 | N/A | 0.455±0.113 | 0.315±0.097 |
| Genitals | RandomForestClassifier with LoG filter | 0.057±0.097 | 0.885±0.076 | 0.000±0.000 | 0.111±0.098 | N/A | 0.473±0.202 | 0.305±0.094 |
| Genitals | RandomForestClassifier with Gradient filter | 0.114±0.148 | 0.869±0.080 | 0.000±0.000 | 0.178±0.076 | N/A | 0.418±0.171 | 0.316±0.095 |
| Genitals | RandomForestClassifier with Square filter | 0.086±0.159 | 0.915±0.021 | 0.100±0.278 | 0.178±0.185 | N/A | 0.491±0.171 | 0.354±0.163 |
| Genitals | RandomForestClassifier with SquareRoot filter | 0.000±0.000 | 0.915±0.040 | 0.000±0.000 | 0.089±0.115 | N/A | 0.545±0.319 | 0.310±0.095 |
| Genitals | RandomForestClassifier with Logarithm filter | 0.086±0.097 | 0.885±0.096 | 0.000±0.000 | 0.111±0.098 | N/A | 0.382±0.147 | 0.293±0.087 |
| Genitals | RandomForestClassifier with Exponential filter | 0.029±0.079 | 0.900±0.072 | 0.000±0.000 | 0.133±0.115 | N/A | 0.436±0.050 | 0.300±0.064 |
| Genitals | RandomForestClassifier with LBP2D filter | 0.171±0.148 | 0.831±0.064 | 0.100±0.278 | 0.244±0.115 | N/A | 0.527±0.124 | 0.375±0.146 |
| Genitals | RandomForestClassifier with Wavelet filter | 0.114±0.148 | 0.900±0.072 | 0.000±0.000 | 0.200±0.205 | N/A | 0.364±0.211 | 0.316±0.127 |
| Genitals | MLPClassifier with Original filter | 0.171±0.194 | 0.708±0.087 | 0.300±0.340 | 0.200±0.227 | N/A | 0.655±0.217 | 0.407±0.213 |
| Genitals | MLPClassifier with LoG filter | 0.343±0.297 | 0.785±0.072 | 0.000±0.000 | 0.333±0.239 | N/A | 0.491±0.294 | 0.390±0.181 |
| Genitals | MLPClassifier with Gradient filter | 0.429±0.125 | 0.623±0.119 | 0.300±0.340 | 0.267±0.231 | N/A | 0.382±0.185 | 0.400±0.200 |
| Genitals | MLPClassifier with Square filter | 0.086±0.097 | 0.754±0.120 | 0.200±0.340 | 0.289±0.157 | N/A | 0.582±0.189 | 0.382±0.181 |
| Genitals | MLPClassifier with SquareRoot filter | 0.143±0.177 | 0.715±0.043 | 0.100±0.278 | 0.200±0.180 | N/A | 0.655±0.147 | 0.363±0.165 |
| Genitals | MLPClassifier with Logarithm filter | 0.200±0.097 | 0.654±0.058 | 0.000±0.000 | 0.222±0.098 | N/A | 0.618±0.094 | 0.339±0.070 |
| Genitals | MLPClassifier with Exponential filter | 0.171±0.079 | 0.762±0.092 | 0.200±0.340 | 0.333±0.218 | N/A | 0.600±0.205 | 0.413±0.187 |
| Genitals | MLPClassifier with LBP2D filter | 0.257±0.194 | 0.685±0.078 | 0.200±0.340 | 0.178±0.123 | N/A | 0.327±0.220 | 0.329±0.191 |
| Genitals | MLPClassifier with Wavelet filter | 0.314±0.194 | 0.685±0.052 | 0.200±0.340 | 0.400±0.209 | N/A | 0.491±0.171 | 0.418±0.193 |
| Genitals | AdaBoostClassifier with Original filter | 0.200±0.202 | 0.715±0.157 | 0.100±0.278 | 0.156±0.076 | N/A | 0.291±0.167 | 0.292±0.176 |
| Genitals | AdaBoostClassifier with LoG filter | 0.200±0.269 | 0.654±0.096 | 0.000±0.000 | 0.444±0.169 | N/A | 0.164±0.167 | 0.292±0.140 |
| Genitals | AdaBoostClassifier with Gradient filter | 0.057±0.097 | 0.654±0.221 | 0.000±0.000 | 0.244±0.180 | N/A | 0.273±0.211 | 0.246±0.142 |
| Genitals | AdaBoostClassifier with Square filter | 0.029±0.079 | 0.638±0.142 | 0.100±0.278 | 0.289±0.209 | N/A | 0.236±0.189 | 0.258±0.179 |
| Genitals | AdaBoostClassifier with SquareRoot filter | 0.200±0.368 | 0.708±0.129 | 0.100±0.278 | 0.089±0.151 | N/A | 0.309±0.220 | 0.281±0.229 |
| Genitals | AdaBoostClassifier with Logarithm filter | 0.143±0.125 | 0.677±0.181 | 0.000±0.000 | 0.200±0.265 | N/A | 0.291±0.094 | 0.262±0.133 |
| Genitals | AdaBoostClassifier with Exponential filter | 0.114±0.148 | 0.638±0.142 | 0.000±0.000 | 0.133±0.151 | N/A | 0.218±0.129 | 0.221±0.114 |
| Genitals | AdaBoostClassifier with LBP2D filter | 0.114±0.079 | 0.685±0.323 | 0.100±0.278 | 0.178±0.157 | N/A | 0.473±0.202 | 0.310±0.208 |
| Genitals | AdaBoostClassifier with Wavelet filter | 0.086±0.159 | 0.692±0.131 | 0.000±0.000 | 0.222±0.258 | N/A | 0.509±0.205 | 0.302±0.151 |
| Genitals | GaussianNB with Original filter | 0.171±0.194 | 0.769±0.101 | 0.500±0.000 | 0.178±0.123 | N/A | 0.145±0.189 | 0.353±0.122 |
| Genitals | GaussianNB with LoG filter | 0.086±0.159 | 0.592±0.105 | 0.200±0.340 | 0.422±0.205 | N/A | 0.255±0.124 | 0.311±0.186 |
| Genitals | GaussianNB with Gradient filter | 0.143±0.125 | 0.023±0.043 | 0.300±0.340 | 0.644±0.180 | N/A | 0.036±0.062 | 0.229±0.150 |
| Genitals | GaussianNB with Square filter | 0.257±0.231 | 0.531±0.160 | 0.700±0.340 | 0.044±0.076 | N/A | 0.127±0.101 | 0.332±0.182 |
| Genitals | GaussianNB with SquareRoot filter | 0.286±0.251 | 0.754±0.099 | 0.500±0.000 | 0.156±0.157 | N/A | 0.073±0.094 | 0.354±0.120 |
| Genitals | GaussianNB with Logarithm filter | 0.114±0.148 | 0.785±0.087 | 0.000±0.000 | 0.244±0.227 | N/A | 0.109±0.094 | 0.250±0.111 |
| Genitals | GaussianNB with Exponential filter | 0.229±0.202 | 0.469±0.109 | 0.600±0.278 | 0.156±0.157 | N/A | 0.164±0.147 | 0.323±0.179 |
| Genitals | GaussianNB with LBP2D filter | 0.143±0.177 | 0.523±0.099 | 0.600±0.278 | 0.200±0.062 | N/A | 0.055±0.062 | 0.304±0.136 |
| Genitals | GaussianNB with Wavelet filter | 0.086±0.238 | 0.623±0.201 | 0.300±0.340 | 0.467±0.115 | N/A | 0.109±0.124 | 0.317±0.204 |
| Other skin | LogisticRegression with Original filter | 0.640±0.324 | 0.050±0.139 | 0.491±0.220 | 0.447±0.122 | 0.567±0.113 | 0.491±0.062 | 0.448±0.163 |
| Other skin | LogisticRegression with LoG filter | 0.480±0.136 | 0.550±0.340 | 0.564±0.217 | 0.447±0.176 | 0.617±0.057 | 0.436±0.217 | 0.516±0.190 |
| Other skin | LogisticRegression with Gradient filter | 0.320±0.283 | 0.300±0.260 | 0.527±0.124 | 0.400±0.095 | 0.483±0.170 | 0.418±0.101 | 0.408±0.172 |
| Other skin | LogisticRegression with Square filter | 0.560±0.324 | 0.000±0.000 | 0.509±0.189 | 0.471±0.052 | 0.567±0.185 | 0.473±0.257 | 0.430±0.168 |
| Other skin | LogisticRegression with SquareRoot filter | 0.720±0.136 | 0.050±0.139 | 0.491±0.171 | 0.447±0.160 | 0.567±0.153 | 0.418±0.101 | 0.449±0.143 |
| Other skin | LogisticRegression with Logarithm filter | 0.480±0.283 | 0.000±0.000 | 0.400±0.260 | 0.412±0.115 | 0.533±0.238 | 0.436±0.167 | 0.377±0.177 |
| Other skin | LogisticRegression with Exponential filter | 0.360±0.324 | 0.050±0.139 | 0.455±0.211 | 0.318±0.151 | 0.567±0.278 | 0.400±0.189 | 0.358±0.215 |
| Other skin | LogisticRegression with LBP2D filter | 0.320±0.377 | 0.050±0.139 | 0.436±0.124 | 0.553±0.151 | 0.667±0.274 | 0.345±0.094 | 0.395±0.193 |
| Other skin | LogisticRegression with Wavelet filter | 0.520±0.136 | 0.150±0.170 | 0.509±0.129 | 0.553±0.160 | 0.617±0.202 | 0.509±0.205 | 0.476±0.167 |
| Other skin | GBDT with Original filter | 0.520±0.222 | 0.100±0.170 | 0.509±0.234 | 0.494±0.111 | 0.550±0.057 | 0.473±0.202 | 0.441±0.166 |
| Other skin | GBDT with LoG filter | 0.440±0.111 | 0.300±0.405 | 0.600±0.305 | 0.565±0.083 | 0.533±0.202 | 0.600±0.205 | 0.506±0.218 |
| Other skin | GBDT with Gradient filter | 0.440±0.272 | 0.150±0.170 | 0.509±0.129 | 0.529±0.103 | 0.483±0.135 | 0.455±0.211 | 0.428±0.170 |
| Other skin | GBDT with Square filter | 0.400±0.304 | 0.100±0.170 | 0.491±0.101 | 0.447±0.111 | 0.450±0.139 | 0.455±0.113 | 0.390±0.156 |
| Other skin | GBDT with SquareRoot filter | 0.400±0.176 | 0.150±0.278 | 0.473±0.094 | 0.518±0.227 | 0.517±0.185 | 0.455±0.080 | 0.419±0.173 |
| Other skin | GBDT with Logarithm filter | 0.520±0.283 | 0.000±0.000 | 0.400±0.234 | 0.506±0.197 | 0.467±0.215 | 0.473±0.167 | 0.394±0.183 |
| Other skin | GBDT with Exponential filter | 0.480±0.283 | 0.050±0.139 | 0.527±0.185 | 0.400±0.095 | 0.517±0.113 | 0.455±0.211 | 0.405±0.171 |
| Other skin | GBDT with LBP2D filter | 0.440±0.208 | 0.050±0.139 | 0.473±0.245 | 0.553±0.168 | 0.550±0.260 | 0.327±0.234 | 0.399±0.209 |
| Other skin | GBDT with Wavelet filter | 0.440±0.272 | 0.200±0.139 | 0.527±0.257 | 0.553±0.133 | 0.633±0.157 | 0.382±0.124 | 0.456±0.180 |
| Other skin | RidgeClassifier with Original filter | 0.280±0.283 | 0.000±0.000 | 0.509±0.260 | 0.506±0.133 | 0.583±0.073 | 0.382±0.094 | 0.377±0.141 |
| Other skin | RidgeClassifier with LoG filter | 0.440±0.208 | 0.150±0.170 | 0.709±0.094 | 0.553±0.111 | 0.667±0.103 | 0.473±0.231 | 0.499±0.153 |
| Other skin | RidgeClassifier with Gradient filter | 0.240±0.208 | 0.050±0.139 | 0.418±0.171 | 0.412±0.089 | 0.567±0.212 | 0.309±0.101 | 0.333±0.153 |
| Other skin | RidgeClassifier with Square filter | 0.320±0.283 | 0.000±0.000 | 0.582±0.205 | 0.424±0.108 | 0.500±0.000 | 0.382±0.281 | 0.368±0.146 |
| Other skin | RidgeClassifier with SquareRoot filter | 0.280±0.222 | 0.000±0.000 | 0.509±0.129 | 0.541±0.061 | 0.533±0.188 | 0.364±0.080 | 0.371±0.113 |
| Other skin | RidgeClassifier with Logarithm filter | 0.360±0.368 | 0.000±0.000 | 0.455±0.160 | 0.447±0.111 | 0.517±0.185 | 0.400±0.101 | 0.363±0.154 |
| Other skin | RidgeClassifier with Exponential filter | 0.320±0.377 | 0.050±0.139 | 0.527±0.202 | 0.353±0.073 | 0.667±0.127 | 0.345±0.217 | 0.377±0.189 |
| Other skin | RidgeClassifier with LBP2D filter | 0.320±0.377 | 0.000±0.000 | 0.509±0.129 | 0.588±0.146 | 0.700±0.215 | 0.218±0.205 | 0.389±0.179 |
| Other skin | RidgeClassifier with Wavelet filter | 0.440±0.208 | 0.200±0.260 | 0.545±0.226 | 0.576±0.120 | 0.617±0.188 | 0.473±0.185 | 0.475±0.198 |
| Other skin | SVM with Original filter | 0.400±0.248 | 0.050±0.139 | 0.291±0.185 | 0.635±0.061 | 0.633±0.157 | 0.436±0.245 | 0.408±0.173 |
| Other skin | SVM with LoG filter | 0.320±0.283 | 0.200±0.139 | 0.564±0.217 | 0.659±0.120 | 0.650±0.153 | 0.509±0.205 | 0.484±0.186 |
| Other skin | SVM with Gradient filter | 0.200±0.176 | 0.000±0.000 | 0.418±0.234 | 0.553±0.083 | 0.567±0.199 | 0.364±0.080 | 0.350±0.129 |
| Other skin | SVM with Square filter | 0.360±0.368 | 0.000±0.000 | 0.309±0.205 | 0.647±0.146 | 0.583±0.194 | 0.291±0.167 | 0.365±0.180 |
| Other skin | SVM with SquareRoot filter | 0.280±0.136 | 0.050±0.139 | 0.364±0.160 | 0.612±0.065 | 0.517±0.113 | 0.418±0.260 | 0.373±0.146 |
| Other skin | SVM with Logarithm filter | 0.240±0.208 | 0.000±0.000 | 0.291±0.094 | 0.647±0.137 | 0.467±0.139 | 0.418±0.272 | 0.344±0.142 |
| Other skin | SVM with Exponential filter | 0.360±0.324 | 0.000±0.000 | 0.291±0.147 | 0.553±0.111 | 0.583±0.179 | 0.273±0.080 | 0.343±0.140 |
| Other skin | SVM with LBP2D filter | 0.280±0.333 | 0.000±0.000 | 0.527±0.269 | 0.659±0.108 | 0.650±0.268 | 0.400±0.101 | 0.419±0.180 |
| Other skin | SVM with Wavelet filter | 0.320±0.283 | 0.000±0.000 | 0.400±0.220 | 0.647±0.103 | 0.550±0.118 | 0.436±0.217 | 0.392±0.157 |
| Other skin | KNN with Original filter | 0.760±0.324 | 0.200±0.260 | 0.382±0.147 | 0.482±0.131 | 0.400±0.087 | 0.418±0.129 | 0.440±0.179 |
| Other skin | KNN with LoG filter | 0.320±0.136 | 0.400±0.278 | 0.382±0.185 | 0.376±0.176 | 0.400±0.087 | 0.345±0.167 | 0.371±0.172 |
| Other skin | KNN with Gradient filter | 0.360±0.324 | 0.150±0.170 | 0.545±0.265 | 0.365±0.108 | 0.367±0.057 | 0.182±0.138 | 0.328±0.177 |
| Other skin | KNN with Square filter | 0.680±0.136 | 0.200±0.260 | 0.400±0.205 | 0.459±0.108 | 0.467±0.118 | 0.200±0.094 | 0.401±0.154 |
| Other skin | KNN with SquareRoot filter | 0.440±0.208 | 0.250±0.219 | 0.236±0.189 | 0.459±0.080 | 0.383±0.118 | 0.400±0.171 | 0.361±0.164 |
| Other skin | KNN with Logarithm filter | 0.440±0.111 | 0.100±0.170 | 0.509±0.171 | 0.376±0.083 | 0.400±0.135 | 0.200±0.124 | 0.338±0.132 |
| Other skin | KNN with Exponential filter | 0.640±0.272 | 0.050±0.139 | 0.364±0.178 | 0.353±0.186 | 0.383±0.118 | 0.345±0.147 | 0.356±0.173 |
| Other skin | KNN with LBP2D filter | 0.320±0.377 | 0.150±0.170 | 0.382±0.217 | 0.435±0.122 | 0.450±0.202 | 0.236±0.129 | 0.329±0.203 |
| Other skin | KNN with Wavelet filter | 0.760±0.208 | 0.300±0.260 | 0.291±0.147 | 0.482±0.080 | 0.433±0.212 | 0.345±0.217 | 0.435±0.187 |
| Other skin | GaussianProcessClassifier with Original filter | 0.640±0.324 | 0.000±0.000 | 0.564±0.167 | 0.529±0.089 | 0.650±0.170 | 0.400±0.062 | 0.464±0.135 |
| Other skin | GaussianProcessClassifier with LoG filter | 0.440±0.478 | 0.350±0.471 | 0.400±0.456 | 0.341±0.405 | 0.367±0.430 | 0.291±0.352 | 0.365±0.432 |
| Other skin | GaussianProcessClassifier with Gradient filter | 0.120±0.136 | 0.000±0.000 | 0.455±0.160 | 0.482±0.061 | 0.583±0.243 | 0.382±0.167 | 0.337±0.128 |
| Other skin | GaussianProcessClassifier with Square filter | 0.480±0.136 | 0.000±0.000 | 0.509±0.205 | 0.518±0.061 | 0.650±0.170 | 0.291±0.202 | 0.408±0.129 |
| Other skin | GaussianProcessClassifier with SquareRoot filter | 0.520±0.222 | 0.000±0.000 | 0.436±0.094 | 0.459±0.095 | 0.583±0.146 | 0.418±0.205 | 0.403±0.127 |
| Other skin | GaussianProcessClassifier with Logarithm filter | 0.360±0.208 | 0.000±0.000 | 0.418±0.101 | 0.506±0.098 | 0.617±0.118 | 0.345±0.167 | 0.374±0.115 |
| Other skin | GaussianProcessClassifier with Exponential filter | 0.360±0.272 | 0.000±0.000 | 0.491±0.129 | 0.435±0.133 | 0.650±0.185 | 0.164±0.124 | 0.350±0.140 |
| Other skin | GaussianProcessClassifier with LBP2D filter | 0.400±0.351 | 0.000±0.000 | 0.582±0.189 | 0.612±0.111 | 0.717±0.157 | 0.073±0.094 | 0.397±0.150 |
| Other skin | GaussianProcessClassifier with Wavelet filter | 0.560±0.272 | 0.000±0.000 | 0.527±0.124 | 0.553±0.122 | 0.650±0.135 | 0.473±0.147 | 0.460±0.133 |
| Other skin | DecisionTreeClassifier with Original filter | 0.240±0.408 | 0.000±0.000 | 0.345±0.313 | 0.447±0.083 | 0.517±0.224 | 0.273±0.080 | 0.304±0.185 |
| Other skin | DecisionTreeClassifier with LoG filter | 0.320±0.283 | 0.150±0.278 | 0.455±0.080 | 0.435±0.190 | 0.617±0.188 | 0.400±0.171 | 0.396±0.198 |
| Other skin | DecisionTreeClassifier with Gradient filter | 0.240±0.324 | 0.050±0.139 | 0.509±0.220 | 0.459±0.233 | 0.433±0.170 | 0.309±0.171 | 0.333±0.210 |
| Other skin | DecisionTreeClassifier with Square filter | 0.080±0.222 | 0.100±0.278 | 0.400±0.294 | 0.400±0.158 | 0.500±0.194 | 0.327±0.205 | 0.301±0.225 |
| Other skin | DecisionTreeClassifier with SquareRoot filter | 0.080±0.136 | 0.150±0.170 | 0.291±0.352 | 0.518±0.222 | 0.450±0.270 | 0.327±0.171 | 0.303±0.220 |
| Other skin | DecisionTreeClassifier with Logarithm filter | 0.240±0.272 | 0.000±0.000 | 0.273±0.211 | 0.459±0.167 | 0.317±0.113 | 0.309±0.234 | 0.266±0.166 |
| Other skin | DecisionTreeClassifier with Exponential filter | 0.080±0.222 | 0.000±0.000 | 0.473±0.292 | 0.365±0.182 | 0.483±0.199 | 0.400±0.260 | 0.300±0.193 |
| Other skin | DecisionTreeClassifier with LBP2D filter | 0.160±0.324 | 0.000±0.000 | 0.509±0.205 | 0.424±0.174 | 0.400±0.170 | 0.400±0.325 | 0.315±0.200 |
| Other skin | DecisionTreeClassifier with Wavelet filter | 0.320±0.283 | 0.100±0.170 | 0.436±0.202 | 0.388±0.234 | 0.583±0.164 | 0.218±0.171 | 0.341±0.204 |
| Other skin | RandomForestClassifier with Original filter | 0.320±0.333 | 0.050±0.139 | 0.509±0.260 | 0.529±0.146 | 0.550±0.139 | 0.327±0.151 | 0.381±0.195 |
| Other skin | RandomForestClassifier with LoG filter | 0.120±0.222 | 0.050±0.139 | 0.509±0.189 | 0.612±0.083 | 0.617±0.057 | 0.418±0.234 | 0.388±0.154 |
| Other skin | RandomForestClassifier with Gradient filter | 0.200±0.248 | 0.050±0.139 | 0.400±0.129 | 0.494±0.083 | 0.550±0.093 | 0.400±0.247 | 0.349±0.156 |
| Other skin | RandomForestClassifier with Square filter | 0.120±0.136 | 0.000±0.000 | 0.291±0.217 | 0.529±0.089 | 0.533±0.157 | 0.400±0.260 | 0.312±0.143 |
| Other skin | RandomForestClassifier with SquareRoot filter | 0.280±0.136 | 0.000±0.000 | 0.345±0.094 | 0.659±0.174 | 0.500±0.103 | 0.418±0.129 | 0.367±0.106 |
| Other skin | RandomForestClassifier with Logarithm filter | 0.200±0.248 | 0.000±0.000 | 0.345±0.050 | 0.612±0.183 | 0.467±0.188 | 0.327±0.220 | 0.325±0.148 |
| Other skin | RandomForestClassifier with Exponential filter | 0.160±0.208 | 0.000±0.000 | 0.382±0.167 | 0.459±0.158 | 0.500±0.103 | 0.218±0.101 | 0.286±0.123 |
| Other skin | RandomForestClassifier with LBP2D filter | 0.320±0.333 | 0.000±0.000 | 0.418±0.171 | 0.576±0.120 | 0.650±0.153 | 0.364±0.211 | 0.388±0.165 |
| Other skin | RandomForestClassifier with Wavelet filter | 0.320±0.333 | 0.000±0.000 | 0.382±0.094 | 0.576±0.061 | 0.633±0.139 | 0.473±0.124 | 0.397±0.125 |
| Other skin | MLPClassifier with Original filter | 0.520±0.222 | 0.050±0.139 | 0.545±0.226 | 0.506±0.083 | 0.567±0.170 | 0.455±0.138 | 0.440±0.163 |
| Other skin | MLPClassifier with LoG filter | 0.400±0.176 | 0.450±0.405 | 0.473±0.094 | 0.518±0.150 | 0.633±0.118 | 0.473±0.147 | 0.491±0.182 |
| Other skin | MLPClassifier with Gradient filter | 0.480±0.451 | 0.100±0.278 | 0.527±0.167 | 0.388±0.160 | 0.417±0.164 | 0.345±0.147 | 0.376±0.228 |
| Other skin | MLPClassifier with Square filter | 0.440±0.324 | 0.000±0.000 | 0.509±0.205 | 0.459±0.108 | 0.567±0.199 | 0.491±0.305 | 0.411±0.190 |
| Other skin | MLPClassifier with SquareRoot filter | 0.680±0.136 | 0.100±0.170 | 0.473±0.147 | 0.447±0.133 | 0.533±0.173 | 0.545±0.080 | 0.463±0.140 |
| Other skin | MLPClassifier with Logarithm filter | 0.320±0.283 | 0.000±0.000 | 0.345±0.124 | 0.447±0.197 | 0.717±0.118 | 0.400±0.205 | 0.372±0.155 |
| Other skin | MLPClassifier with Exponential filter | 0.520±0.283 | 0.100±0.278 | 0.455±0.178 | 0.353±0.103 | 0.433±0.199 | 0.473±0.269 | 0.389±0.219 |
| Other skin | MLPClassifier with LBP2D filter | 0.360±0.538 | 0.000±0.000 | 0.345±0.147 | 0.459±0.108 | 0.583±0.253 | 0.291±0.094 | 0.340±0.190 |
| Other skin | MLPClassifier with Wavelet filter | 0.640±0.208 | 0.150±0.170 | 0.400±0.129 | 0.541±0.061 | 0.567±0.212 | 0.509±0.171 | 0.468±0.158 |
| Other skin | AdaBoostClassifier with Original filter | 0.200±0.176 | 0.100±0.278 | 0.327±0.294 | 0.306±0.260 | 0.350±0.199 | 0.491±0.062 | 0.296±0.211 |
| Other skin | AdaBoostClassifier with LoG filter | 0.240±0.208 | 0.150±0.170 | 0.400±0.305 | 0.376±0.234 | 0.333±0.366 | 0.218±0.362 | 0.286±0.274 |
| Other skin | AdaBoostClassifier with Gradient filter | 0.240±0.208 | 0.350±0.170 | 0.455±0.366 | 0.118±0.089 | 0.533±0.118 | 0.091±0.138 | 0.298±0.182 |
| Other skin | AdaBoostClassifier with Square filter | 0.120±0.222 | 0.050±0.139 | 0.473±0.342 | 0.341±0.233 | 0.600±0.087 | 0.309±0.189 | 0.315±0.202 |
| Other skin | AdaBoostClassifier with SquareRoot filter | 0.440±0.324 | 0.200±0.260 | 0.327±0.362 | 0.200±0.197 | 0.467±0.270 | 0.236±0.305 | 0.312±0.286 |
| Other skin | AdaBoostClassifier with Logarithm filter | 0.560±0.272 | 0.000±0.000 | 0.364±0.444 | 0.365±0.457 | 0.350±0.383 | 0.164±0.185 | 0.300±0.290 |
| Other skin | AdaBoostClassifier with Exponential filter | 0.280±0.377 | 0.100±0.170 | 0.382±0.292 | 0.224±0.196 | 0.417±0.194 | 0.255±0.124 | 0.276±0.225 |
| Other skin | AdaBoostClassifier with LBP2D filter | 0.160±0.208 | 0.150±0.278 | 0.345±0.257 | 0.341±0.227 | 0.450±0.280 | 0.255±0.147 | 0.284±0.233 |
| Other skin | AdaBoostClassifier with Wavelet filter | 0.160±0.272 | 0.050±0.139 | 0.509±0.379 | 0.341±0.209 | 0.650±0.236 | 0.382±0.124 | 0.349±0.227 |
| Other skin | GaussianNB with Original filter | 0.760±0.208 | 0.550±0.260 | 0.473±0.281 | 0.247±0.095 | 0.583±0.127 | 0.382±0.094 | 0.499±0.177 |
| Other skin | GaussianNB with LoG filter | 0.720±0.416 | 0.650±0.416 | 0.582±0.171 | 0.129±0.061 | 0.550±0.139 | 0.455±0.288 | 0.514±0.248 |
| Other skin | GaussianNB with Gradient filter | 0.640±0.324 | 0.450±0.260 | 0.800±0.292 | 0.176±0.073 | 0.483±0.268 | 0.291±0.202 | 0.473±0.236 |
| Other skin | GaussianNB with Square filter | 0.640±0.324 | 0.500±0.219 | 0.600±0.189 | 0.259±0.133 | 0.150±0.087 | 0.455±0.113 | 0.434±0.177 |
| Other skin | GaussianNB with SquareRoot filter | 0.760±0.272 | 0.650±0.170 | 0.527±0.257 | 0.271±0.065 | 0.517±0.185 | 0.345±0.050 | 0.512±0.167 |
| Other skin | GaussianNB with Logarithm filter | 0.800±0.248 | 0.400±0.278 | 0.436±0.094 | 0.235±0.186 | 0.600±0.224 | 0.182±0.080 | 0.442±0.185 |
| Other skin | GaussianNB with Exponential filter | 0.640±0.324 | 0.150±0.278 | 0.236±0.129 | 0.200±0.122 | 0.433±0.170 | 0.509±0.151 | 0.361±0.196 |
| Other skin | GaussianNB with LBP2D filter | 0.840±0.208 | 0.250±0.219 | 0.582±0.062 | 0.212±0.065 | 0.383±0.173 | 0.127±0.101 | 0.399±0.138 |
| Other skin | GaussianNB with Wavelet filter | 0.440±0.324 | 0.550±0.260 | 0.691±0.344 | 0.235±0.052 | 0.450±0.238 | 0.491±0.101 | 0.476±0.220 |
| Anus | LogisticRegression with Original filter | N/A | N/A | N/A | 0.733±0.346 | N/A | 0.733±0.185 | 0.733±0.266 |
| Anus | LogisticRegression with LoG filter | N/A | N/A | N/A | 0.600±0.540 | N/A | 0.533±0.227 | 0.567±0.383 |
| Anus | LogisticRegression with Gradient filter | N/A | N/A | N/A | 0.667±0.507 | N/A | 0.733±0.346 | 0.700±0.427 |
| Anus | LogisticRegression with Square filter | N/A | N/A | N/A | 0.667±0.414 | N/A | 0.533±0.227 | 0.600±0.320 |
| Anus | LogisticRegression with SquareRoot filter | N/A | N/A | N/A | 0.667±0.293 | N/A | 0.800±0.370 | 0.733±0.331 |
| Anus | LogisticRegression with Logarithm filter | N/A | N/A | N/A | 0.600±0.346 | N/A | 0.800±0.370 | 0.700±0.358 |
| Anus | LogisticRegression with Exponential filter | N/A | N/A | N/A | 0.600±0.540 | N/A | 0.400±0.346 | 0.500±0.443 |
| Anus | LogisticRegression with LBP2D filter | N/A | N/A | N/A | 0.533±0.370 | N/A | 0.533±0.370 | 0.533±0.370 |
| Anus | LogisticRegression with Wavelet filter | N/A | N/A | N/A | 0.600±0.453 | N/A | 0.800±0.227 | 0.700±0.340 |
| Anus | GBDT with Original filter | N/A | N/A | N/A | 0.467±0.472 | N/A | 0.667±0.293 | 0.567±0.382 |
| Anus | GBDT with LoG filter | N/A | N/A | N/A | 0.733±0.346 | N/A | 0.667±0.293 | 0.700±0.319 |
| Anus | GBDT with Gradient filter | N/A | N/A | N/A | 0.467±0.472 | N/A | 0.733±0.346 | 0.600±0.409 |
| Anus | GBDT with Square filter | N/A | N/A | N/A | 0.600±0.540 | N/A | 0.667±0.507 | 0.633±0.523 |
| Anus | GBDT with SquareRoot filter | N/A | N/A | N/A | 0.467±0.628 | N/A | 0.867±0.227 | 0.667±0.427 |
| Anus | GBDT with Logarithm filter | N/A | N/A | N/A | 0.400±0.540 | N/A | 0.733±0.346 | 0.567±0.443 |
| Anus | GBDT with Exponential filter | N/A | N/A | N/A | 0.600±0.453 | N/A | 0.733±0.346 | 0.667±0.400 |
| Anus | GBDT with LBP2D filter | N/A | N/A | N/A | 0.533±0.227 | N/A | 0.667±0.293 | 0.600±0.260 |
| Anus | GBDT with Wavelet filter | N/A | N/A | N/A | 0.467±0.370 | N/A | 0.867±0.227 | 0.667±0.298 |
| Anus | RidgeClassifier with Original filter | N/A | N/A | N/A | 0.667±0.414 | N/A | 0.800±0.227 | 0.733±0.320 |
| Anus | RidgeClassifier with LoG filter | N/A | N/A | N/A | 0.600±0.540 | N/A | 0.467±0.227 | 0.533±0.383 |
| Anus | RidgeClassifier with Gradient filter | N/A | N/A | N/A | 0.667±0.507 | N/A | 0.733±0.346 | 0.700±0.427 |
| Anus | RidgeClassifier with Square filter | N/A | N/A | N/A | 0.667±0.507 | N/A | 0.533±0.227 | 0.600±0.367 |
| Anus | RidgeClassifier with SquareRoot filter | N/A | N/A | N/A | 0.733±0.346 | N/A | 0.867±0.370 | 0.800±0.358 |
| Anus | RidgeClassifier with Logarithm filter | N/A | N/A | N/A | 0.600±0.346 | N/A | 0.800±0.370 | 0.700±0.358 |
| Anus | RidgeClassifier with Exponential filter | N/A | N/A | N/A | 0.600±0.540 | N/A | 0.533±0.370 | 0.567±0.455 |
| Anus | RidgeClassifier with LBP2D filter | N/A | N/A | N/A | 0.467±0.472 | N/A | 0.533±0.370 | 0.500±0.421 |
| Anus | RidgeClassifier with Wavelet filter | N/A | N/A | N/A | 0.600±0.453 | N/A | 0.800±0.227 | 0.700±0.340 |
| Anus | SVM with Original filter | N/A | N/A | N/A | 0.667±0.293 | N/A | 0.733±0.185 | 0.700±0.239 |
| Anus | SVM with LoG filter | N/A | N/A | N/A | 0.600±0.540 | N/A | 0.600±0.346 | 0.600±0.443 |
| Anus | SVM with Gradient filter | N/A | N/A | N/A | 0.600±0.540 | N/A | 0.533±0.370 | 0.567±0.455 |
| Anus | SVM with Square filter | N/A | N/A | N/A | 0.200±0.227 | N/A | 0.600±0.540 | 0.400±0.383 |
| Anus | SVM with SquareRoot filter | N/A | N/A | N/A | 0.533±0.472 | N/A | 0.867±0.227 | 0.700±0.349 |
| Anus | SVM with Logarithm filter | N/A | N/A | N/A | 0.533±0.227 | N/A | 0.867±0.227 | 0.700±0.227 |
| Anus | SVM with Exponential filter | N/A | N/A | N/A | 0.200±0.370 | N/A | 0.667±0.507 | 0.433±0.439 |
| Anus | SVM with LBP2D filter | N/A | N/A | N/A | 0.333±0.293 | N/A | 0.600±0.453 | 0.467±0.373 |
| Anus | SVM with Wavelet filter | N/A | N/A | N/A | 0.200±0.227 | N/A | 0.800±0.555 | 0.500±0.391 |
| Anus | KNN with Original filter | N/A | N/A | N/A | 0.800±0.227 | N/A | 0.600±0.185 | 0.700±0.206 |
| Anus | KNN with LoG filter | N/A | N/A | N/A | 0.667±0.414 | N/A | 0.533±0.227 | 0.600±0.320 |
| Anus | KNN with Gradient filter | N/A | N/A | N/A | 0.533±0.472 | N/A | 0.533±0.472 | 0.533±0.472 |
| Anus | KNN with Square filter | N/A | N/A | N/A | 0.467±0.472 | N/A | 0.533±0.227 | 0.500±0.349 |
| Anus | KNN with SquareRoot filter | N/A | N/A | N/A | 0.467±0.370 | N/A | 0.733±0.346 | 0.600±0.358 |
| Anus | KNN with Logarithm filter | N/A | N/A | N/A | 0.733±0.185 | N/A | 0.867±0.227 | 0.800±0.206 |
| Anus | KNN with Exponential filter | N/A | N/A | N/A | 0.400±0.453 | N/A | 0.533±0.227 | 0.467±0.340 |
| Anus | KNN with LBP2D filter | N/A | N/A | N/A | 0.467±0.227 | N/A | 0.533±0.370 | 0.500±0.298 |
| Anus | KNN with Wavelet filter | N/A | N/A | N/A | 0.667±0.414 | N/A | 0.467±0.370 | 0.567±0.392 |
| Anus | GaussianProcessClassifier with Original filter | N/A | N/A | N/A | 0.733±0.346 | N/A | 0.533±0.472 | 0.633±0.409 |
| Anus | GaussianProcessClassifier with LoG filter | N/A | N/A | N/A | 0.600±0.540 | N/A | 0.467±0.370 | 0.533±0.455 |
| Anus | GaussianProcessClassifier with Gradient filter | N/A | N/A | N/A | 0.733±0.540 | N/A | 0.467±0.555 | 0.600±0.547 |
| Anus | GaussianProcessClassifier with Square filter | N/A | N/A | N/A | 0.400±0.346 | N/A | 0.533±0.370 | 0.467±0.358 |
| Anus | GaussianProcessClassifier with SquareRoot filter | N/A | N/A | N/A | 0.733±0.346 | N/A | 0.467±0.555 | 0.600±0.451 |
| Anus | GaussianProcessClassifier with Logarithm filter | N/A | N/A | N/A | 0.667±0.293 | N/A | 0.667±0.507 | 0.667±0.400 |
| Anus | GaussianProcessClassifier with Exponential filter | N/A | N/A | N/A | 0.533±0.472 | N/A | 0.267±0.346 | 0.400±0.409 |
| Anus | GaussianProcessClassifier with LBP2D filter | N/A | N/A | N/A | 0.667±0.414 | N/A | 0.267±0.346 | 0.467±0.380 |
| Anus | GaussianProcessClassifier with Wavelet filter | N/A | N/A | N/A | 0.733±0.540 | N/A | 0.200±0.555 | 0.467±0.547 |
| Anus | DecisionTreeClassifier with Original filter | N/A | N/A | N/A | 0.533±0.472 | N/A | 0.733±0.346 | 0.633±0.409 |
| Anus | DecisionTreeClassifier with LoG filter | N/A | N/A | N/A | 0.533±0.555 | N/A | 0.533±0.370 | 0.533±0.463 |
| Anus | DecisionTreeClassifier with Gradient filter | N/A | N/A | N/A | 0.533±0.472 | N/A | 0.800±0.227 | 0.667±0.349 |
| Anus | DecisionTreeClassifier with Square filter | N/A | N/A | N/A | 0.533±0.472 | N/A | 0.533±0.370 | 0.533±0.421 |
| Anus | DecisionTreeClassifier with SquareRoot filter | N/A | N/A | N/A | 0.467±0.628 | N/A | 0.800±0.227 | 0.633±0.427 |
| Anus | DecisionTreeClassifier with Logarithm filter | N/A | N/A | N/A | 0.400±0.540 | N/A | 0.733±0.346 | 0.567±0.443 |
| Anus | DecisionTreeClassifier with Exponential filter | N/A | N/A | N/A | 0.733±0.346 | N/A | 0.733±0.346 | 0.733±0.346 |
| Anus | DecisionTreeClassifier with LBP2D filter | N/A | N/A | N/A | 0.600±0.346 | N/A | 0.733±0.346 | 0.667±0.346 |
| Anus | DecisionTreeClassifier with Wavelet filter | N/A | N/A | N/A | 0.600±0.185 | N/A | 0.867±0.227 | 0.733±0.206 |
| Anus | RandomForestClassifier with Original filter | N/A | N/A | N/A | 0.667±0.293 | N/A | 0.667±0.414 | 0.667±0.353 |
| Anus | RandomForestClassifier with LoG filter | N/A | N/A | N/A | 0.600±0.453 | N/A | 0.667±0.293 | 0.633±0.373 |
| Anus | RandomForestClassifier with Gradient filter | N/A | N/A | N/A | 0.400±0.453 | N/A | 0.600±0.346 | 0.500±0.400 |
| Anus | RandomForestClassifier with Square filter | N/A | N/A | N/A | 0.400±0.453 | N/A | 0.667±0.293 | 0.533±0.373 |
| Anus | RandomForestClassifier with SquareRoot filter | N/A | N/A | N/A | 0.667±0.293 | N/A | 0.667±0.293 | 0.667±0.293 |
| Anus | RandomForestClassifier with Logarithm filter | N/A | N/A | N/A | 0.733±0.346 | N/A | 0.600±0.185 | 0.667±0.266 |
| Anus | RandomForestClassifier with Exponential filter | N/A | N/A | N/A | 0.600±0.540 | N/A | 0.533±0.555 | 0.567±0.547 |
| Anus | RandomForestClassifier with LBP2D filter | N/A | N/A | N/A | 0.800±0.227 | N/A | 0.667±0.293 | 0.733±0.260 |
| Anus | RandomForestClassifier with Wavelet filter | N/A | N/A | N/A | 0.600±0.346 | N/A | 0.533±0.227 | 0.567±0.286 |
| Anus | MLPClassifier with Original filter | N/A | N/A | N/A | 0.800±0.227 | N/A | 0.733±0.185 | 0.767±0.206 |
| Anus | MLPClassifier with LoG filter | N/A | N/A | N/A | 0.600±0.540 | N/A | 0.600±0.346 | 0.600±0.443 |
| Anus | MLPClassifier with Gradient filter | N/A | N/A | N/A | 0.533±0.472 | N/A | 0.733±0.346 | 0.633±0.409 |
| Anus | MLPClassifier with Square filter | N/A | N/A | N/A | 0.600±0.346 | N/A | 0.467±0.227 | 0.533±0.286 |
| Anus | MLPClassifier with SquareRoot filter | N/A | N/A | N/A | 0.667±0.293 | N/A | 0.800±0.227 | 0.733±0.260 |
| Anus | MLPClassifier with Logarithm filter | N/A | N/A | N/A | 0.600±0.185 | N/A | 0.800±0.227 | 0.700±0.206 |
| Anus | MLPClassifier with Exponential filter | N/A | N/A | N/A | 0.533±0.472 | N/A | 0.400±0.346 | 0.467±0.409 |
| Anus | MLPClassifier with LBP2D filter | N/A | N/A | N/A | 0.467±0.227 | N/A | 0.467±0.370 | 0.467±0.298 |
| Anus | MLPClassifier with Wavelet filter | N/A | N/A | N/A | 0.600±0.346 | N/A | 0.533±0.472 | 0.567±0.409 |
| Anus | AdaBoostClassifier with Original filter | N/A | N/A | N/A | 0.533±0.472 | N/A | 0.533±0.227 | 0.533±0.349 |
| Anus | AdaBoostClassifier with LoG filter | N/A | N/A | N/A | 0.667±0.507 | N/A | 0.867±0.227 | 0.767±0.367 |
| Anus | AdaBoostClassifier with Gradient filter | N/A | N/A | N/A | 0.600±0.346 | N/A | 0.533±0.227 | 0.567±0.286 |
| Anus | AdaBoostClassifier with Square filter | N/A | N/A | N/A | 0.733±0.346 | N/A | 0.733±0.540 | 0.733±0.443 |
| Anus | AdaBoostClassifier with SquareRoot filter | N/A | N/A | N/A | 0.667±0.507 | N/A | 0.867±0.227 | 0.767±0.367 |
| Anus | AdaBoostClassifier with Logarithm filter | N/A | N/A | N/A | 0.400±0.540 | N/A | 0.667±0.414 | 0.533±0.477 |
| Anus | AdaBoostClassifier with Exponential filter | N/A | N/A | N/A | 0.533±0.472 | N/A | 0.733±0.346 | 0.633±0.409 |
| Anus | AdaBoostClassifier with LBP2D filter | N/A | N/A | N/A | 0.467±0.472 | N/A | 0.800±0.227 | 0.633±0.349 |
| Anus | AdaBoostClassifier with Wavelet filter | N/A | N/A | N/A | 0.400±0.453 | N/A | 0.600±0.346 | 0.500±0.400 |
| Anus | GaussianNB with Original filter | N/A | N/A | N/A | 0.667±0.293 | N/A | 0.533±0.227 | 0.600±0.260 |
| Anus | GaussianNB with LoG filter | N/A | N/A | N/A | 0.467±0.472 | N/A | 0.667±0.293 | 0.567±0.382 |
| Anus | GaussianNB with Gradient filter | N/A | N/A | N/A | 0.600±0.346 | N/A | 0.533±0.227 | 0.567±0.286 |
| Anus | GaussianNB with Square filter | N/A | N/A | N/A | 0.600±0.346 | N/A | 0.467±0.227 | 0.533±0.286 |
| Anus | GaussianNB with SquareRoot filter | N/A | N/A | N/A | 0.733±0.346 | N/A | 0.933±0.185 | 0.833±0.266 |
| Anus | GaussianNB with Logarithm filter | N/A | N/A | N/A | 0.400±0.185 | N/A | 0.800±0.227 | 0.600±0.206 |
| Anus | GaussianNB with Exponential filter | N/A | N/A | N/A | 0.600±0.346 | N/A | 0.533±0.370 | 0.567±0.358 |
| Anus | GaussianNB with LBP2D filter | N/A | N/A | N/A | 0.733±0.346 | N/A | 0.533±0.227 | 0.633±0.286 |
| Anus | GaussianNB with Wavelet filter | N/A | N/A | N/A | 0.333±0.414 | N/A | 0.667±0.414 | 0.500±0.414 |
