# Supplementary material for: Radiomics analysis for the early diagnosis of common sexually transmitted infections and skin lesions
Source: PLOS Digit Health. 2025 Jul 23;4(7):e0000926. doi: 10.1371/journal.pdig.0000926 (PMC12286352; doi:10.1371/journal.pdig.0000926)
Supplement: Attachment — Submitted filename: 0.Point-by-Point Response to Reviewers.docx [file pdig.0000926.s014.docx]

**Point-by-Point Response to Reviewers**

We sincerely appreciate both reviewers' valuable comments and suggestions. These detailed reviews have significantly contributed to improving the clarity and accuracy of our manuscript. We have carefully reviewed all comments and addressed them point by point, as detailed below:

- Line 14-126 The authors asked specialists with extensive clinical experience to confirm the presence of an STI. It seems there is a lack of golden standard to determine STI. It is not sure how reliable about the subjective clinical judgment. The authors need to address this.

**Author response:** We appreciate the reviewer’s comment. The STI Atlas dataset we used includes cases that have undergone serological and molecular testing, which serves as the gold standard for STI diagnosis. We have clarified this in the revised manuscript.

**Changes: (Page 6)** *We developed models for the diagnosis of skin lesions from four common sexually transmitted infections and two skin lesions. We built these models by combining radiomics technology and deep learning classifiers. Before building models, we collected STI images from STI ATLAS (https://stiatlas.org/), which supplied skin images with infection types and body sites. All diagnoses for the cases in the STI Atlas dataset have been confirmed with serological and/or molecular testing, serving as the gold standard for STI diagnosis.*

- Line 114-115. The authors used two STI specialists to make manual segmentation to label the infection area. Is there any inter-observer and intra-observer studies to validate the reliability of the segmentation method? If there is any discrepancies among the specialists, what will be done to make the final decision?

**Author response:** To ensure the reliability of manual segmentation, we conducted both inter-observer and intra-observer agreement analyses. We have now clarified this in the revised manuscript.

**Changes: (Page 6)** *To ensure the reliability of manual segmentation, two STI specialists independently segmented these images, with inter-observer agreement evaluated by a third research member. Additionally, one specialist re-evaluated the same subset after a one-month interval to assess intra-observer consistency. In cases of discrepancies, the specialists discussed and reached a consensus to finalise the segmentation.*

- The study needs to use accuracy, specificity and sensitivity to illustrate the results. I am afraid that since there are imbalance data in each category of skin lesion, this will lead to result with high sensitivity and low specificity. The authors should consider this.

**Author response:** We appreciate the reviewer’s valuable feedback. We acknowledge the potential impact of data imbalance on the model’s sensitivity and specificity. To address this concern, we have used AUC (Area Under the Curve) as a more robust evaluation metric, along with the confusion matrix, which provides a more comprehensive representation of the model’s performance in the presence of class imbalance. Additionally, to further address the reviewer’s concerns, we have included the accuracy, specificity, and sensitivity results in the supplement and provided a discussion on data imbalance in our Supplementary Tables.

**Changes: (Supplements)** *Table S2-S9.*

**(Page 16)** *Our study also has several limitations. Firstly, the types of infections covered in our dataset are limited. We only focused on a small range of infection types commonly captured in our clinic.* *Secondly, there was an imbalance in the number of images representing each infection type within our dataset. We captured as many skin images as we could, but infections in some body sites are relatively rare. This imbalance contributed to the discrepancy between sensitivity and specificity in our model.*

**Reviewer 2**

- Line 111: 4 common STIs and 2 skin lesions - the lesions of concern are reported in 210-212 and perhaps this could have been made clearer in the Methods. The only common STI discussed in Line 48-49 that seems relevant in this study, is syphilis; whereas the author(s) states that the mots global prevalent STIs are trichomiasis, gonococcal and chlamydial infection. The title is mis-leading in that the study looked at infections of genital lesion / ulcers / genital dermatosis.

**Author response:** Thank you for your insightful comment. We acknowledge that our initial wording may have led to some misinterpretation regarding the scope of the study. To provide greater clarity, we have revised the Background and Methods sections to state our focus explicitly. Additionally, we have clarified our selection of four STIs (early syphilis, herpes, warts, and molluscum contagiosum) and two general skin lesions (lichen sclerosus and tinea) across three key body sites. These revisions ensure that our study’s scope aligns with the specific dermatological manifestations investigated.

**Changes:** **(Page 3)** *Sexually transmitted infections (STIs) pose a major public health challenge. According to the World Health Organization (WHO), an estimated 374 million new infections with trichomoniasis, chlamydia, gonorrhoea, and syphilis occurred globally in 2020 (1).*

**(Page 11)** *We focused on four STIs and two skin lesions across three key body sites: genitals, anus, and other skin areas. “Genitals” referred to external genitalia, including the penis, scrotum, vulva, and perineum. “Anus” specifically denoted the perianal region. “Other skin” referred to areas excluding the genitals and anus, covering other body regions. These prevalent conditions captured in our dataset included sexually transmitted infections that caused skin lesions, such as secondary syphilis (143 cases), herpes (128 cases), warts (127 cases), and molluscum contagiosum (66 cases), as well as general skin lesions, including lichen sclerosus (145 cases) and tinea (61 cases).*

- Line 136: LabelMe tool - there is no reference as to what this 'tool' is - was it developed as part of this study; and if so, has it been validated / standardised. Need more information / reference to this 'tool'.

**Author response:** LabelMe is an open-source tool for image annotation and was not developed as part of this study. We have now included a reference to its official repository to provide more details about the tool.

**Changes:** **(Page 7)** *The accuracy of the labelled regions has a crucial impact on the model accuracy. We utilised the LabelMe tool (23) for ROI labelling. To ensure consistency and reliability, JJS initially trained JT and CKF on the use of the LabelMe tool, emphasising the importance of expanding segmentation areas beyond the immediate infection to capture subtle changes like colour variations.*

**(Page 20)** *23.Wada K. Labelme: Image Polygonal Annotation with Python [cited 2025 March 20]. Available from: https://github.com/wkentaro/labelme.*

- Line 97 - 101 and 323: Has syphilis been diagnostically 'misclassified' as 'secondary syphilis'?

Was radiomics analysis able to define between a primary syphilitic chancre (particularly in the genital / anus) to secondary manifestations (such as Condylomata lata, mucous patches) with / without a 'skin' rash? If radiomics analysis was unable to distinguish these, I suggest using the term "Early Syphilis" versus "Secondary syphilis"

**Author response:** In this study, we focused on secondary syphilis, as the dataset included images of its characteristic manifestations, such as condylomata lata and mucous patches, rather than primary chancres. Radiomics analysis was not specifically designed to differentiate between primary and secondary syphilis lesions. However, we acknowledge the potential for misclassification in clinical settings and agree that “**Early Syphilis**” may be a more encompassing term. We have revised the entire manuscript to reflect this.

- Line 214 / 218 - site of photograph; Table S1, S2, S3: The sites of the photographs need better clarity - what sites are 'genitals' ? (name sites) as well as "Skin" - e.g. keratinised skin versus 'non-keratinised"

**Author response:** We acknowledge the need for clearer definitions of anatomical sites. In our dataset, “Genitals” refers to external genitalia, including the penis, scrotum, vulva, and perineum. “Anus” specifically denotes the perianal region. “Skin” refers to areas excludes the genitals and anus, covering other body regions. We have revised our manuscript and tables to clarify these definitions explicitly.

**Changes: (Supplements)** *Supplement Table 1. General information of STIs images dataset.*

| **Infection class** | **Infection type** | **Infection site** | **Images num** |
| --- | --- | --- | --- |
| Sexually transmitted infections caused skin lesions | Herpes | Genitals | 36 |
|  | (n=61) | Other skin | 25 |
|  | Molluscum Contagiosum | Genitals | 12 |
|  | (n=66) | Other skin | 54 |
|  | Secondary Syphilis | Genitals | 45 |
|  | (n=143) | Anus | 12 |
|  |  | Other skin | 86 |
|  | Warts | Genitals | 54 |
|  | (n=121) | Anus | 15 |
|  |  | Other skin | 52 |
| General skin lesions | Lichen Sclerosus | Genitals | 127 |
|  | (n=145) | Other skin | 18 |
|  | Tinea (n=61) | Other skin | 61 |

- Line 366 and 377. Reference 1 and Reference 6 need to be clearly written per acaedemic standards. Was the source the World Health Organisation (WHO); and it would be preferably followed by a link to a website and when this was last accessed.

**Author response:** We appreciate the reviewer’s comment and have revised References 1 and 6 to align with academic citation standards. We have explicitly mentioned the World Health Organization (WHO) as the source and included the corresponding URLs along with the date of last access.

**Changes: (Page 19)** *1. World Health Organization. Sexually transmitted infections (STIs) [cited 2025 March 20]. Available from:* [*https://www.who.int/news-room/fact-sheets/detail/sexually-transmitted-infections-(stis)*](https://www.who.int/news-room/fact-sheets/detail/sexually-transmitted-infections-(stis))

*6. World Health Organization. Global health sector strategies on, respectively, HIV, viral hepatitis and sexually transmitted infections for the period 2022-2030 2022 [cited 2025 March 20]. Available from:* [*https://www.who.int/publications/i/item/9789240053779*](https://www.who.int/publications/i/item/9789240053779)*.*

- Line 420: Reference 22 - this reference is not sufficiently provided as is. Can this reference be linked to a webpage and last accessed please?

**Author response:** We have revised Reference 22 to include a direct link to the STI ATLAS webpage and have provided the date it was last accessed.

**Changes: (Page 20)** *22. Morton A, Bradshaw C, Fairley C, Lee D, Henzell H, Williams H, et al. STIATLAS [cited 2025 March 20]. Available from:* [*https://stiatlas.org/*](https://stiatlas.org/) *.*

- Figure 1: This figure took me some time to decipher and is another way of showing AUCs; perhaps could be clearer in terms of graphics for readers.

**Author response:** We fully understand your concerns regarding the clarity of the figure. To enhance readers’ comprehension and interpretation of the results, we have completely redesigned Figure 1.

**Changes: (Figure 1)** *Models’ AUC (×10²) with nine images filter and eleven classifiers on (a) unspecific sites; (b) genitals; (c) anus; (d) other skin.*

**ABC: Adaptive Boosting Classifier, DTC: Decision Tree Classifier, GBDT: Gradient Boosted Decision Trees, GNB: Gaussian Naive Bayes, GPC: Gaussian Process Classifier, KNN: K-nearest Neighbors, LR: Logistic Regression, MLPC: Multi-layer Perceptron Classifier, RC: RidgeClassifier, RFC: Random Forest Classifier, SVM: Support Vector Machine.*
